# Global, regional, and national burden of periodontal diseases from 1990 to 2021 and predictions to 2040: an analysis of the global burden of disease study 2021

**DOI:** 10.3389/froh.2025.1627746

**Published:** 2025-07-24

**Authors:** Meiling Hu, Ruibin Zhang, Ren Wang, Yan Wang, Jincai Guo

**Affiliations:** ^1^ Department of Pharmacy, Changsha Stomatological Hospital, Changsha, China; ^2^ School of Stomatology, Hunan University of Chinese Medicine, Changsha, China; ^3^ School of Pharmacy, Hunan University of Chinese Medicine, Changsha, China

**Keywords:** periodontal diseases, periodontitis, oral health, epidemiology, global burden

## Abstract

**Background:**

Periodontal diseases are one of the most prevalent oral diseases globally, with increasing incidence rates in recent years. This study aims to analyze the global burden and epidemiological trends of periodontal diseases from 1990 to 2021, and to forecast trends through 2040.

**Methods:**

Data on incidence, prevalence, and disability-adjusted life-years (DALYs) of periodontal diseases were extracted from the Global Burden of Disease study 2021. The analysis evaluated the global, regional, and national burden of periodontal diseases, conducted a decomposition analysis based on population growth, aging, and epidemiological changes, and examined the association between the sociodemographic index (SDI) and disease burden, and projected trends through 2040.

**Results:**

In 2021, global estimates indicated 89,613,534 (95% uncertainty interval (UI): 79,069,091–101,005,642) incident cases, 1,066,953,744 (95% UI: 896,546,186–1,234,839,287) prevalent cases, and 6,903,284 (95% UI: 2,772,284–14,106,182) DALYs due to periodontal diseases. South Asia demonstrated the highest number and age-standardized rates (ASR) of incidence, prevalence, and DALYs. Between 1990 and 2021, the ASR of prevalence and DALYs increased slightly, while the ASR of incidence remained stable worldwide. Population growth and aging were the primary contributors to changes in disease burden. A significant negative correlation was observed between the SDI and the periodontal diseases burden. Projections from 2022 to 2040 indicate annual increases in both the number and ASR of incidence, prevalence, and DALYs for periodontal diseases.

**Conclusions:**

Periodontal diseases remain a substantial global health challenge, characterized by rising incidence, prevalence, and DALYs. These findings underscore the urgent need to implement effective prevention strategies and integrate oral health services into primary care, particularly in regions with low to middle SDI, to reduce the escalating burden.

## 1 Introduction

Periodontal diseases are chronic inflammatory conditions that affect the gingiva and surrounding tissues, causing irreversible damage to the alveolar bone and periodontal ligament (1). This damage may result in tooth loss and considerably compromise oral health (2). These conditions are strongly associated with systemic diseases, including diabetes (3), cardiovascular diseases (4), and Alzheimer's disease (5). Periodontal diseases present a substantial public health challenge as one of the most prevalent oral diseases globally (6). According to the Global Burden of Disease Study (GBD) 2019, periodontal diseases affected over 1 billion individuals worldwide (7), imposing a considerable economic burden (8). In 2018, these conditions incurred direct costs of $3.49 billion in the United States and €2.52 billion in Europe, with indirect costs reaching $150.57 and €156.12 billion, respectively (8).

The quantification of periodontal diseases burden facilitates evaluating their impact on public health and society, informing evidence-based policy development. Previous research using earlier GBD editions and recent analyses of GBD 2021 data have enhanced the understanding of the disease burden (9–17); nonetheless, certain limitations persist. Two studies reported only descriptive analyses of burden trends (10, 11), whereas two focused solely on disease prevalence (12, 13). Nascimento et al. and Wu et al. examined periodontitis alongside edentulism and caries (14, 15), while other researchers focused on specific populations, countries, or regions (9, 16, 17). Notable research gaps exist in exploring the drivers of disease burden through decomposition analysis and the correlations between the disease burden and the socio-demographic index (SDI). A comprehensive understanding of these trends, encompassing all age groups and integrating incidence, prevalence, and disability-adjusted life years (DALYs), is warranted.

This study, therefore, aims to present a comprehensive analysis of the global burden and temporal trends of periodontal diseases from 1990 to 2021. Specifically, it encompasses descriptive epidemiology, temporal trend analysis using Joinpoint regression, decomposition analysis of driving factors, an evaluation of correlations between the SDI and disease burden, and projections of global trends through 2040.

## 2 Materials and methods

### 2.1 Data sources

The GBD 2021 encompassed 371 diseases and injuries and 88 risk factors across 21 regions and 204 countries/territories (18). To address gaps in incomplete datasets across age, time, and location, the data were modeled using spatiotemporal Gaussian process regression (ST-GPR), whereas the disease model, Bayesian meta-regression (DisMod-MR 2.1), ensured internal consistency of estimates. The comprehensive methodology and estimation processes are described elsewhere (18). This study analyzed data on the incidence, prevalence, and disability-adjusted life years (DALYs) in counts and their age-standardized rates (ASR) for periodontal diseases across 17 age groups (spanning 15–19 years to ≥95 years, in 5-year intervals) in 21 regions and 204 countries/territories. Data were extracted from the Global Health Data Exchange GBD Results Tool (https://vizhub.healthdata.org/gbd-results/). Additionally, the SDI, a composite indicator reflecting socioeconomic status and demographic characteristics, was incorporated. SDI values range from 0 to 1 and are categorized into five quintiles: high, high-middle, middle, low-middle, and low (18). This study adheres to the Guidelines for Accurate and Transparent Health Estimates Reporting (19). Ethical review and informed consent were not required because the study utilized publicly available data.

### 2.2 Definition of periodontal diseases

In GBD 2021, periodontal diseases were defined using a hierarchical approach, with Community Periodontal Index of Treatment Needs (CPITN) Class 4 as the primary criterion. An attachment loss (AL) >6 mm or pocket depth (PD) >5 mm served as equivalent reference definitions, which required no crosswalking. For cases using varied threshold definitions, alternative definitions (CPITN Class 3, AL >5 mm, or AL >4 mm) were utilized based on meta-regression, Bayesian, regularized, trimmed (MR-BRT) crosswalking, to address systematic biases across case definitions (18). This study analyzed pre-aggregated modeled estimates from the GBD 2021 database; therefore, no additional inclusion/exclusion criteria were implemented. The corresponding International Classification of Diseases (ICD) codes were K05-K06.9 for ICD-10, and 523–523.9 for ICD-9 (18).

### 2.3 Statistical analyses

**Figure 1:**
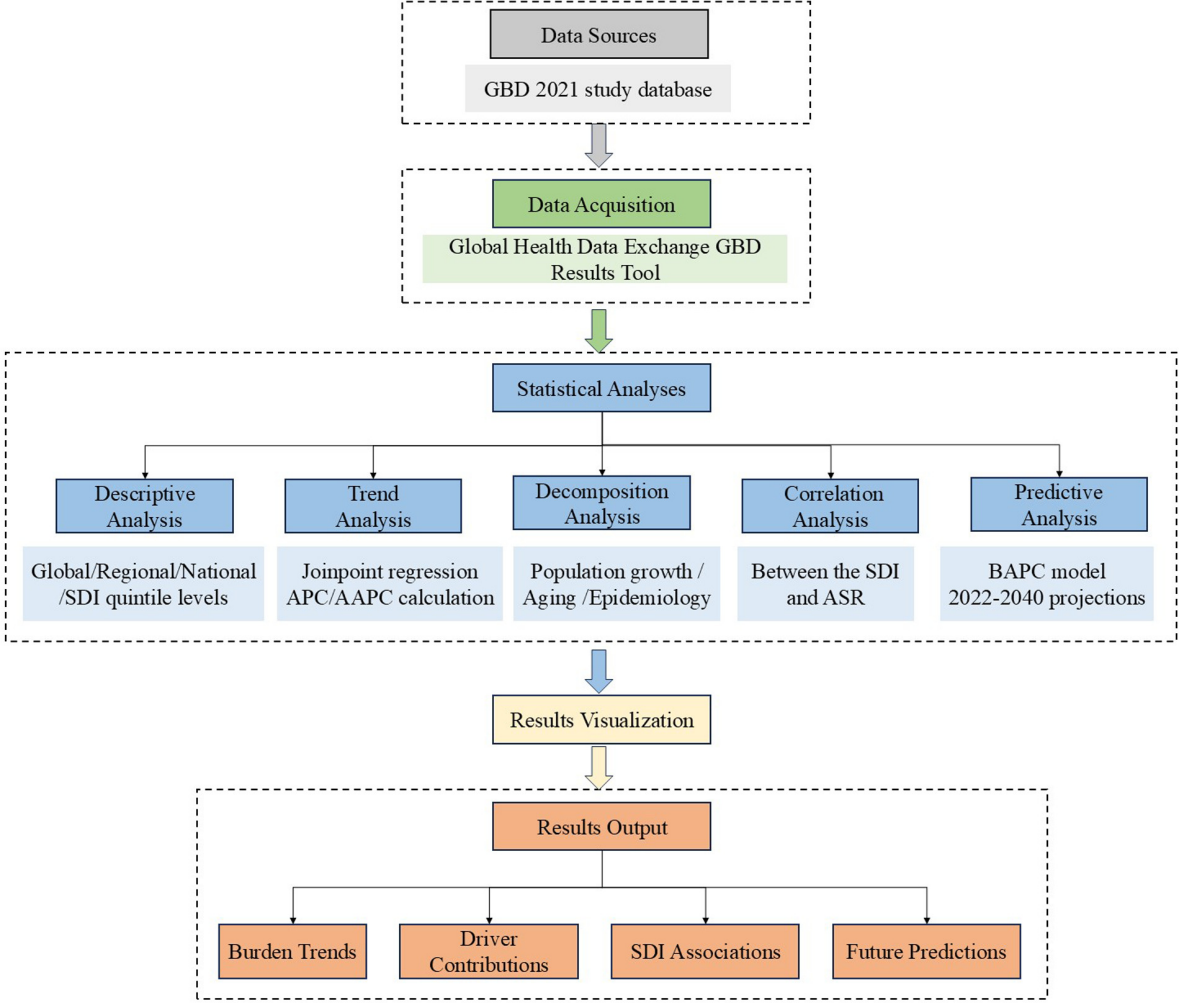
Methodological workflow diagram of the study.

Figure 1 presents a methodological workflow diagram, providing a comprehensive overview of the research process. This diagram illustrates the systematic progression from data collection to statistical analyses, emphasizing the interconnections among steps.

#### 2.3.1 Descriptive analysis

Descriptive analysis was conducted at global, regional, national, and SDI quintile levels to evaluate the burden of periodontal diseases. This analysis compared incidence, prevalence, and DALYs using case numbers and ASR based on GBD 2021 data. All rates are expressed per 100,000 population.

#### 2.3.2 Trend analysis

Temporal trends in periodontal diseases were evaluated using Joinpoint regression. The methodology is detailed on the official website (https://surveillance.cancer.gov/joinpoint/). This analysis comprised three steps: first, segmented regression was conducted using a log-linear model with the grid search method to identify potential joinpoints; second, mean squared errors (MSE) were calculated for each scenario, and the grid point showing the minimum MSE selected as the joinpoint; finally, the optimal joinpoint regression model was validated using the Monte Carlo permutation test. The analysis quantified trends in ASR of incidence, prevalence, and DALYs of periodontal diseases from 1990 to 2021 using the annual percent change (APC) and average annual percent change (AAPC), derived from the optimal model. The AAPC represents the overall epidemiological trend from 1990 to 2021. It is calculated as the weighted average of segment-specific APCs. An APC or AAPC >0 or <0 indicates an increasing or decreasing trend, respectively. In contrast, a value approaching zero suggests a stable trend. Analysis was conducted using the National Cancer Institute's Joinpoint regression program software (version 5.2.0).

#### 2.3.3 Decomposition analysis

To analyze factors driving the changes in DALYs from 1990 to 2021, a decomposition analysis was conducted based on population aging, population growth, and epidemiological change. This analysis utilized methods developed by Das Gupta (20), where the standardized impact of each multiplicative factor is isolated algebraically to determine the individual contributions to the observed changes. Regarding disease burden, the contribution of each factor represents its impact on total DALYs, assuming other drivers remain constant across years. A positive contribution indicates an increase in total DALYs, whereas a negative contribution indicates a decrease. The total contribution of all drivers equals the total change in DALYs.

#### 2.3.4 Correlation analysis of ASR with SDI

The Spearman correlation coefficient was utilized to examine the correlation between the SDI and ASR of periodontal diseases across 21 GBD regions. This nonparametric measure evaluates the strength and direction of monotonic association between two variables. The coefficient ranges from −1 to +1, with positive values indicating a positive correlation and negative values indicating a negative correlation. The correlation strength increases as the coefficient approaches 1, whereas values closer to 0 indicate weak correlations. Statistical significance was determined by a *p*-value <0.05.

#### 2.3.5 Predictive analysis

To facilitate improved public health policymaking, the global burden of periodontal diseases from 2022 to 2040 was predicted using the Bayesian Age-Period-Cohort (BAPC) model. This model incorporates the age-specific population data from 1990 to 2021, demographic standardization data (18), and population predictions from the 2024 revision of the World Population Prospects (21). The model's fundamental assumptions focus on decomposing and smoothing age, period, and cohort effects. Mathematically, the model can be expressed as:

y(a,p,c)=α(a)+β(p)+γ(c)+ε(a,p,c)

where *y*(*a*, *p*, *c*) denotes the observed outcome for a specific combination of age (*a*), time period (*p*), and birth cohort (*c*). *α*(*a*), *β*(*p*), and *γ*(*c*) represent age, period, and cohort effects, respectively, and *ε*(*a*, *p*, *c*) represents the residual error term (22, 23). The model assumes that adjacent time-point effects are similar. To smooth these effects, it incorporates a second-order random walk (RW2) with inverse-gamma priors. The RW2 assumes that the second differences of all time effects follow independent mean-zero normal distributions, a property that identifies second differences in APC models for natural smoothing (24). The BAPC model utilizes the Integrated Nested Laplace Approximation (INLA) to estimate posterior marginal distributions and conduct extrapolations based on age, period, and cohort effects (25). The combination of BAPC and INLA circumvents mixing and convergence issues commonly associated with Markov Chain Monte Carlo sampling techniques (26, 27). Notably, BAPC provides superior coverage and accuracy than alternative prediction methods (25, 28).

All analyses and visualizations were conducted using R program (version 4.4.1), utilizing the “maps” package to generate world maps, the “BAPC and INLA” package for BAPC modeling, and the “ggplot2” package for data visualization.

## 3 Results

### 3.1 Descriptive analysis of the burden of periodontal diseases

**Figure 2:**
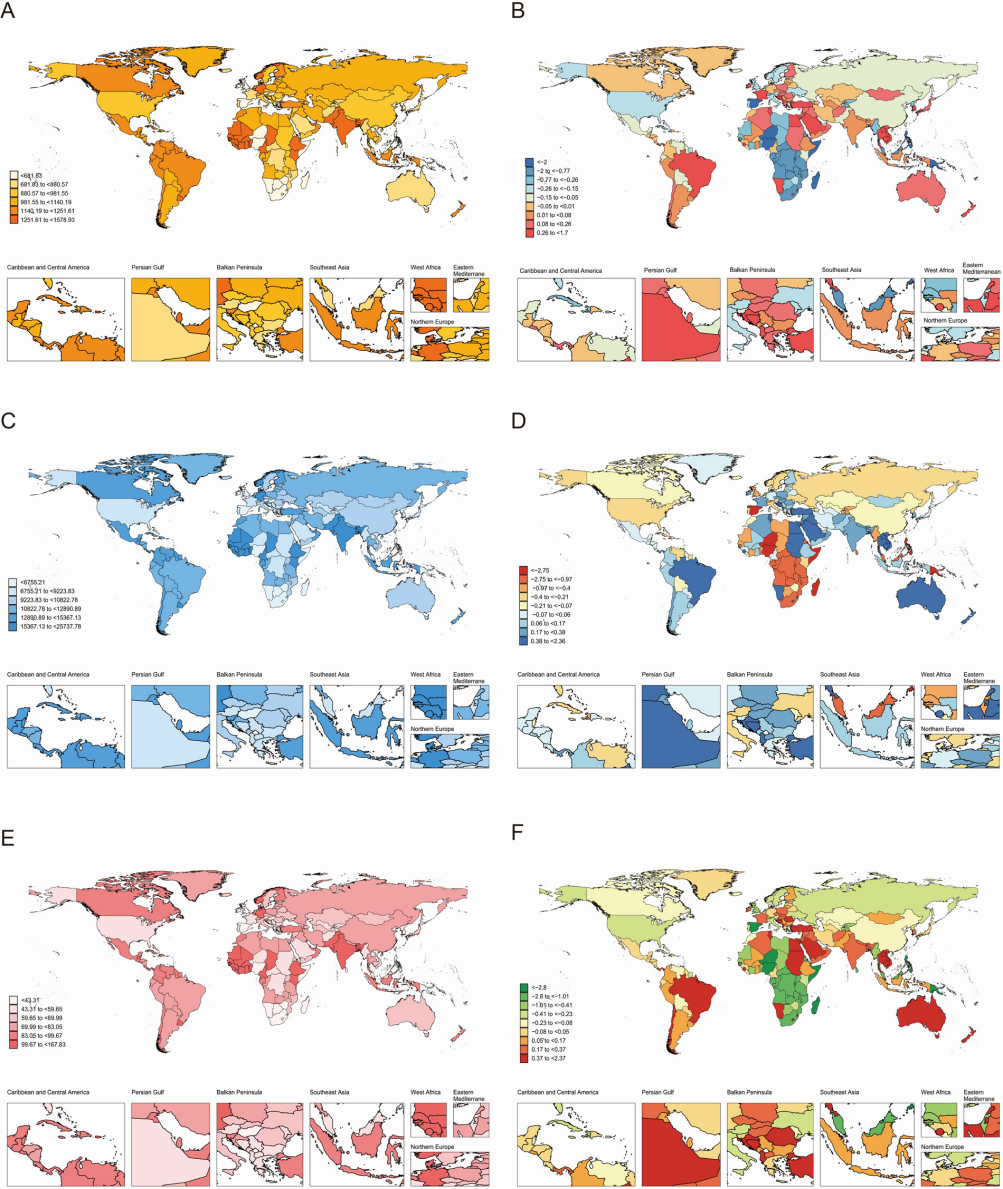
Global burden of periodontal diseases across 204 countries and territories. **(A)** ASR of incidence in 2021; **(B)** AAPC of incidence, 1990–2021; **(C)** ASR of prevalence in 2021; **(D)** AAPC of prevalence, 1990–2021; **(E)** ASR of DALYs in 2021; **(F)** AAPC of DALYs, 1990–2021. AAPC, average annual percentage change; ASR, age-standardized rate; DALYs, disability-adjusted life-years.

Globally, the case numbers of incidence, prevalence, and DALYs for periodontal diseases increased substantially from 1990 to 2021. Nonetheless, the corresponding ASR demonstrated minimal variations (Tables 1–3). Regionally, South Asia recorded the highest cases and ASR for all three metrics in 2021, but Oceania reported the lowest (Tables 1–3). Nationally, India had the highest incidence, prevalence, and DALYs, followed by China, and Tokelau reported the lowest. Sierra Leone reported the highest ASR across all measures, whereas Kiribati had the lowest (Figure 2; Supplementary Tables S1–S3). In terms of SDI quintiles, middle SDI regions demonstrated the highest cases in 2021, and low SDI regions showed the lowest. Conversely, low-middle SDI regions displayed the highest ASR, but high SDI regions showed the lowest (Tables 1–3).

**Table 1:** The case number and ASR of incidence of periodontal diseases in 1990 and 2021 at global, SDI, and GBD region levels, with AAPC from 1990 to 2021.

Location	1990		2021		AAPC (95%CI) 1990–2021	*P*
	Number (95%UI)	ASR (95%UI)	Number (95%UI)	ASR (95%UI)		
Global	50,823,934 (39,615,908–59,174,250)	1,069.38 (852.99–1,239.59)	89,613,534 (79,069,091–101,005,642)	1,069.44 (942.71–1,204.58)	0 (−0.03 to 0.02)	0.777
SDI quintiles						
High SDI	94,07,307 (75,22,992–11,034,311)	932.72 (744.76–1,092.57)	13,683,899 (11,977,317–15,679,772)	927.32 (793.31–1,084.81)	−0.02 (−0.04 to 0.01)	0.272
High-middle SDI	10,535,594 (82,68,653–12,506,988)	985.52 (771.35–1,170.18)	16,372,283 (14,237,282–18,707,352)	970.97 (832.75–1,128.41)	−0.05 (−0.1 to −0.01)	0.029
Middle SDI	15,240,268 (11,775,330–17,960,032)	1,064.46 (857.67–1,231.66)	29,049,440 (25,504,028–32,385,126)	1,064.11 (933.75–1,186.6)	0 (−0.02 to 0.03)	0.893
Low-middle SDI	10,856,413 (83,16,650–12,736,447)	1,193.01 (946.99–1,367.83)	21,624,748 (18,713,530–24,468,525)	1,174.58 (1,030.71–1,314.31)	−0.05 (−0.08 to −0.03)	<0.001
Low SDI	47,36,383 (36,10,010–55,60,975)	1,285.03 (1,013.31–1,477.37)	88,17,758 (74,66,036–10,258,848)	1,032.5 (884.68–1,174.38)	−0.7 (−0.76 to −0.64)	<0.001
GBD regions						
Andean Latin America	335,714 (280,692–389,115)	1,149.24 (1,000–1,286.59)	763,116 (634,616–918,340)	1,143.29 (960.68–1,349.21)	−0.01 (−0.02 to 0)	0.08
Australasia	191,773 (143,849–237,127)	856.66 (639.99–1,051)	359,593 (295,930–435,523)	931.83 (744.96–1,143.69)	0.27 (0.15 to 0.38)	<0.001
Caribbean	396,095 (310,705–467,206)	1,239.02 (996.6–1,424.35)	581,042 (483,068–678,815)	1,138.31 (943.48–1,335.36)	−0.27 (−0.29 to −0.24)	<0.001
Central Asia	540,130 (414,989–648,594)	981.56 (762.83–1,167.76)	915,222 (715,484–11,29,682)	951.75 (760.91–1,159.99)	−0.1 (−0.13 to −0.08)	<0.001
Central Europe	12,88,006 (10,14,058–15,25,609)	915.1 (711.94–1,086.89)	15,19,605 (12,88,462–17,87,330)	962.94 (786.58–1,160.5)	0.16 (0.15 to 0.17)	<0.001
Central Latin America	15,61,298 (12,04,972–18,40,805)	1,215.36 (984.68–1,385.95)	31,80,018 (27,37,643–36,33,620)	1,196.97 (1,033.36–1,365.93)	−0.05 (−0.06 to −0.04)	<0.001
Central Sub-Saharan Africa	525,993 (393,297–634,980)	1,318.33 (1,037.15–1,526.28)	785,630 (571,748–10,42,683)	845.51 (644.79–1,080.53)	−1.41 (−1.49 to −1.33)	<0.001
East Asia	10,910,643 (85,25,980–13,025,176)	991.88 (785.23–1,170.57)	18,834,272 (16,163,330–21,356,912)	960.22 (823.18–1,094.59)	−0.09 (−0.2 to 0.01)	0.085
Eastern Europe	27,99,238 (22,67,561–32,55,493)	1,083.31 (869.62–1,264.9)	28,41,483 (23,97,274–33,32,064)	1,031.36 (849.56–1,229.99)	−0.16 (−0.17 to −0.14)	<0.001
Eastern Sub-Saharan Africa	17,91,149 (13,41,064–21,57,745)	1,329.33 (1,043.69–1,529.79)	30,88,910 (25,49,108–37,11,334)	998.15 (846.71–1,157.03)	−0.91 (−1.01 to −0.81)	<0.001
High-income Asia Pacific	16,37,363 (12,34,715–19,89,052)	809.97 (613.64–983.61)	24,19,327 (20,06,157–28,83,589)	839.84 (665.34–1,023.86)	0.1 (0.05 to 0.15)	<0.001
High-income North America	31,20,188 (25,27,787–36,64,930)	979.86 (785.52–1,147.54)	44,51,645 (38,83,258–50,53,106)	922.28 (792.49–1,058.1)	−0.2 (−0.28 to −0.13)	<0.001
North Africa and Middle East	22,14,458 (17,05,166–26,69,482)	931.78 (735.48–1,109.36)	64,02,774 (53,34,264–75,93,001)	1,056.6 (902.98–1,238.97)	0.41 (0.38 to 0.43)	<0.001
Oceania	41,008 (30,696–50,831)	926.21 (705.96–1,116.84)	25,514 (18,485–35,150)	261.57 (196.46–359.87)	−3.98 (−4.18 to −3.78)	<0.001
South Asia	11,302,568 (86,63,618–13,369,169)	1,271.29 (1,011.83–1,467.01)	24,065,975 (20,949,359–26,844,234)	1,288.55 (1,138.93–1,428.39)	0.04 (0.03 to 0.05)	<0.001
Southeast Asia	35,65,915 (27,02,164–42,76,233)	981.06 (771.94–1,150.5)	66,56,564 (57,18,890–75,83,742)	898.08 (778.81–1,017.26)	−0.28 (−0.36 to −0.2)	<0.001
Southern Latin America	509,728 (393,994–607,290)	1,070.52 (832.59–1,272.74)	840,317 (683,560–10,02,225)	1,095.32 (884.91–1,317.01)	0.06 (0 to 0.13)	0.066
Southern Sub-Saharan Africa	263,908 (191,099–335,668)	733.87 (533.68–923.67)	414,975 (331,108–523,978)	580.46 (473.17–721.86)	−0.75 (−0.84 to −0.65)	<0.001
Tropical Latin America	13,48,151 (10,34,281–15,95,755)	1,077.65 (868.19–1,253.01)	29,85,415 (25,93,337–33,20,681)	1,161.97 (1,001.28–1,295.82)	0.26 (0.21 to 0.31)	<0.001
Western Europe	42,90,063 (35,23,774–49,63,588)	930.07 (754.44–1,073.31)	49,71,171 (41,80,250–58,87,407)	841.7 (694.51–1,008.33)	−0.32 (−0.38 to −0.25)	<0.001
Western Sub-Saharan Africa	21,90,546 (17,28,609–25,18,020)	1,445.77 (1,164.33–1,619.67)	35,10,966 (30,06,264–40,26,433)	942.95 (818.25–1,075.34)	−1.34 (−1.51 to −1.18)	<0.001

AAPC, average annual percent change; ASR, age-standardized rate; GBD, Global Burden of Disease; and SDI, socio-demographic index.

**Table 2:** The case number and ASR of the prevalence of periodontal diseases in 1990 and 2021 at global, SDI, and GBD region levels, with AAPC from 1990 to 2021.

Location	1990		2021		AAPC (95%CI) 1990–2021	*P*
	Number (95%UI)	ASR (95%UI)	Number (95%UI)	ASR (95%UI)		
Global	557,036,657 (427,553,223–683,752,829)	12,282.44 (9,494.51–15,063.91)	10,66,953,744 (896,546,186–12,34,839,287)	12,498.3 (10,526.8–14,493.37)	0.04 (−0.01 to 0.09)	0.078
SDI quintiles						
High SDI	104,166,404 (81,666,455–127,615,908)	10,232.99 (7,971.34–12,561.65)	159,217,668 (131,067,146–186,552,251)	10,139.68 (8,221–12,189.03)	−0.04 (−0.1 to 0.03)	0.258
High-middle SDI	112,477,527 (84,949,926–141,523,175)	10,693.06 (8,074.04–13,393.12)	190,563,436 (155,170,167–224,654,753)	10,613.49 (8,609.35–12,735)	−0.01 (−0.13 to 0.11)	0.904
Middle SDI	160,561,226 (120,848,014–199,383,146)	12,093.96 (9,332.18–14,918.62)	346,971,125 (294,753,121–402,356,717)	12,326.58 (10,492–14,258.36)	0.05 (−0.01 to 0.12)	0.103
Low-middle SDI	123,559,532 (94,516,572–151,011,946)	14,942.23 (11,651.33–17,901.74)	267,767,360 (222,364,186–311,663,136)	15,252.28 (12,777.21–17,553.49)	0.06 (0.03 to 0.09)	<0.001
Low SDI	55,761,027 (43,082,249–67,824,831)	17,260.05 (13,628.03–20,703.39)	101,686,261 (84,910,682–119,217,456)	13,488.11 (11,346.75–15,555.44)	−0.8 (−0.88 to −0.71)	<0.001
GBD regions						
Andean Latin America	31,51,937 (25,98,434–37,83,360)	11,527.39 (9,502.56–13,667.46)	76,35,406 (58,17,558–97,42,369)	11,561.93 (8,874.73–14,615.47)	0.02 (−0.03 to 0.07)	0.438
Australasia	18,20,176 (13,12,776–23,62,422)	8,189.67 (5,875.13–10,630.99)	39,79,930 (30,16,541–50,74,698)	9,967.23 (7,355.51–13,114.51)	0.64 (0.51 to 0.76)	<0.001
Caribbean	44,93,235 (34,32,770–55,28,154)	15,163.54 (11,723.35–18,400.55)	71,00,123 (55,46,934–87,46,369)	13,674.44 (10,613.75–16,870.06)	−0.33 (−0.37 to −0.29)	<0.001
Central Asia	53,16,806 (39,16,005–67,56,782)	10,125.58 (7,551.03–12,737.2)	90,05,491 (66,40,329–11,750,809)	9,321.12 (6,936.9–12,000.9)	−0.26 (−0.3 to −0.23)	<0.001
Central Europe	13,093,681 (98,45,084–165,515,82)	9,103.73 (6,836.34–11,527.98)	16,809,305 (13,162,273–20,827,563)	10,046.91 (7,649.52–12,924.1)	0.32 (0.26 to 0.38)	<0.001
Central Latin America	16,499,976 (12,363,772–20,453,150)	14,438.4 (11,109.39–17,648.66)	38,015,445 (31,266,040–44,833,316)	14,280.67 (11,767.34–16,795.82)	−0.03 (−0.04 to −0.02)	<0.001
Central Sub-Saharan Africa	58,79,734 (44,30,773–71,49,168)	17,304.98 (13,379.81–20,785.13)	79,89,260 (55,87,545–11,025,203)	9,567.48 (6,904.73–12,492.99)	−1.87 (−1.99 to −1.76)	<0.001
East Asia	113,942,172 (85,561,716–144,637,555)	10,976.83 (8,299.83–13,749.28)	226,131,458 (187,514,178–265,720,506)	10,707.99 (8,785.26–12,667.41)	−0.08 (−0.3 to 0.14)	0.483
Eastern Europe	31,249,552 (24,073,990–38,674,733)	11,734.45 (8,976.69–14,607.9)	31,682,217 (24,748,361–39,210,888)	10,795.79 (8,255.27–13,746.88)	−0.27 (−0.29 to −0.24)	<0.001
Eastern Sub-Saharan Africa	20,722,537 (15,680,439–25,173,613)	18,479.02 (14,492.18–22,175.78)	33,791,770 (27,088,783–41,126,307)	12,722.77 (10,423.37–15,068.77)	−1.2 (−1.26 to −1.14)	<0.001
High-income Asia Pacific	17,938,189 (13,082,223–22,710,007)	8,701.49 (6,367.78–11,044.36)	27,856,863 (21,561,598–33,670,443)	8,923.78 (6,751.26–11,355.43)	0.06 (−0.06 to 0.18)	0.309
High-income North America	32,632,790 (25,453,367–40,852,309)	10,263.74 (7,852.2–12,941.94)	49,830,524 (41,176,016–58,185,121)	9,672.4 (7,977.24–11,544.6)	−0.18 (−0.26 to −0.11)	<0.001
North Africa and Middle East	21,425,361 (15,774,378–27,548,273)	9,646.86 (7,263.77–12,250.9)	69,332,503 (54,652,562–86,046,285)	11,659.64 (9,400.12–14,095.49)	0.62 (0.6 to 0.64)	<0.001
Oceania	404,466 (288,624–525,719)	9,967.03 (7,328.55–12,683.1)	224,853 (159,647–320,105)	2,247.62 (1,641.52–3,060.13)	−4.68 (−5.01 to −4.34)	<0.001
South Asia	131,476,473 (100,032,780–161,641,953)	16,322.47 (12,685.85–19,701.2)	313,142,939 (260,920,583–362,379,652)	17,566.61 (14,730.91–20,136.04)	0.23 (0.18 to 0.27)	<0.001
Southeast Asia	36,813,701 (27,160,424–46,247,746)	10,987.16 (8,342.37–13,655.98)	74,747,686 (61,840,624–88,527,198)	10,039.15 (8,384.41–11,811.93)	−0.29 (−0.37 to −0.21)	<0.001
Southern Latin America	57,66,212 (43,01,658–72,29,116)	12,256.1 (9,152.73–15,371.48)	10,195,931 (78,26,311–12,687,198)	13,003.07 (9,877.71–16,290.93)	0.18 (0.1 to 0.27)	<0.001
Southern Sub-Saharan Africa	23,99,909 (17,02,824–31,30,116)	7,210.42 (5,169.86–9,431.49)	39,29,520 (30,29,724–49,99,651)	5,650.83 (4,446.02–7,121.39)	−0.78 (−0.88 to −0.67)	<0.001
Tropical Latin America	12,939,686 (95,44,867–16,405,768)	10,835.24 (8,138.89–13,618.21)	32,576,679 (26,186,797–38,858,744)	12,379.02 (9,951.57–14,734.12)	0.46 (0.34 to 0.58)	<0.001
Western Europe	50,936,559 (41,133,207–60,859,710)	10,774.18 (8,565.53–12,938.37)	59,818,461 (47,909,449–72,065,116)	9,467.68 (7,449.71–11,601.94)	−0.43 (−0.56 to −0.31)	<0.001
Western Sub-Saharan Africa	28,133,506 (22,525,708–33,075,106)	21,837.14 (18,016.48–25,373.03)	43,157,381 (36,619,999–49,756,993)	13,335.64 (11,504.74–15,189.1)	−1.58 (−1.7 to −1.46)	<0.001

AAPC, average annual percent change; ASR, age-standardized rate; GBD, Global Burden of Disease; and SDI, socio-demographic index.

**Table 3:** The case number and ASR of DALYs of periodontal diseases in 1990 and 2021 at global, SDI and GBD region levels, with AAPC from 1990 to 2021.

Location	1990		2021		AAPC (95%CI) 1990–2021	*P*
	Number (95%UI)	ASR (95%UI)	Number (95%UI)	ASR (95%UI)		
Global	36,20,518 (14,21,409–77,19,685)	79.62 (31.46–169.62)	69,03,284 (27,72,284–14,106,182)	80.89 (32.47–165.37)	0.04 (−0.01 to 0.09)	0.1
SDI quintiles						
High SDI	675,037 (267,679–14,36,739)	66.41 (26.42–141.98)	10,21,198 (406,501–20,47,768)	65.47 (26.3–132.99)	−0.05 (−0.11 to 0.01)	0.107
High-middle SDI	730,382 (288,347–15,76,847)	69.35 (27.67–149.66)	12,31,035 (492,704–24,92,760)	68.73 (27.55–139.24)	−0.06 (−0.15 to 0.03)	0.219
Middle SDI	10,47,099 (414,764–22,36,757)	78.52 (31.2–167.75)	22,49,557 (909,679–45,17,591)	79.88 (32.32–160.93)	0.05 (−0.01 to 0.1)	0.076
Low-middle SDI	802,431 (324,100–16,60,446)	96.49 (38.29–198.97)	17,35,974 (705,992–35,46,781)	98.5 (39.88–199.41)	0.06 (0.03 to 0.09)	<0.001
Low SDI	362,253 (143,525–746,670)	111.43 (44.04–227.49)	660,691 (268,309–13,56,342)	86.99 (35.4–176.97)	−0.79 (−0.88 to −0.7)	<0.001
GBD regions						
Andean Latin America	20,638 (8,526–43,145)	75.24 (30.6–156.62)	49,777 (19,357–104,947)	75.28 (29.58–158.55)	0.01 (−0.04 to 0.07)	0.654
Australasia	11,779 (4,540–26,131)	53.03 (20.48–117.45)	25,605 (10,182–54,013)	64.45 (25.05–136.61)	0.63 (0.45 to 0.81)	<0.001
Caribbean	29,330 (11,795–60,669)	98.79 (39.96–204.03)	45,964 (18,345–95,056)	88.59 (35.16–183.65)	−0.35 (−0.4 to −0.3)	<0.001
Central Asia	34,567 (13,704–74,906)	65.68 (26.1–142.1)	58,379 (23,002–122,809)	60.29 (23.69–125.58)	−0.28 (−0.32 to −0.23)	<0.001
Central Europe	84,525 (33,149–183,760)	58.83 (23.07–128.25)	107,876 (42,681–219,152)	64.88 (25.8–134.29)	0.31 (0.27 to 0.34)	<0.001
Central Latin America	107,627 (43,075–223,982)	93.72 (38–194.92)	246,586 (99,480–506,792)	92.56 (37.33–190.51)	−0.04 (−0.06 to −0.02)	<0.001
Central Sub-Saharan Africa	38,057 (15,189–77,260)	111.25 (44.62–228.84)	51,550 (19,947–112,511)	61.21 (23.79–132.14)	−1.93 (−2.05 to −1.8)	<0.001
East Asia	744,686 (295,676–16,09,368)	71.47 (28.52–154.7)	14,66,661 (578,102–29,97,147)	69.55 (27.77–143.33)	−0.08 (−0.27 to 0.1)	0.376
Eastern Europe	201,222 (81,002–424,926)	75.66 (30.36–159.41)	202,793 (80,698–410,203)	69.39 (27.84–142.56)	−0.27 (−0.3 to −0.23)	<0.001
Eastern Sub-Saharan Africa	134,871 (53,658–274,527)	119.47 (47.55–242.76)	219,739 (89,456–465,543)	82.03 (33.23–169)	−1.2 (−1.26 to −1.14)	<0.001
High-income Asia Pacific	116,842 (45,917–253,870)	56.65 (22.29–123.57)	178,914 (70,629–356,713)	57.8 3 (22.77–117.71)	0.08 (−0.04 to 0.19)	0.197
High-income North America	210,367 (83,442–448,355)	66.3 (26.35–142.02)	316,568 (126,390–636,544)	61.88 (24.94–126.08)	−0.21 (−0.29 to −0.14)	<0.001
North Africa and Middle East	139,422 (54,465–305,549)	62.44 (24.47–135.68)	448,887 (180,266–924,569)	75.15 (30.2–154.11)	0.61 (0.58 to 0.63)	<0.001
Oceania	2,631 (1,025–5,785)	64.39 (25.36–138.53)	1,450 (546–3,197)	14.37 (5.36–31.85)	−4.71 (−5.07 to −4.36)	<0.001
South Asia	852,784 (346,102–17,46,628)	105.2 (42.21–215.03)	20,28,688 (829,374–41,43,642)	113.39 (46.48–230.17)	0.23 (0.2 to 0.27)	<0.001
Southeast Asia	2,40,371 (95,319–5,21,969)	71.35 (28.1–154.11)	4,85,823 (1,95,241–9,71,331)	65.05 (26.09–130.61)	−0.3 (−0.38 to −0.22)	<0.001
Southern Latin America	37,493 (14,773–80,998)	79.65 (31.39–172.16)	65,887 (26,110–1,35,372)	84.17 (33.02–173.78)	0.17 (0.09 to 0.26)	<0.001
Southern Sub-Saharan Africa	15,503 (5,988–33,897)	46.32 (17.87–101.39)	25,046 (9,864–52,962)	35.83 (13.8–75.14)	−0.82 (−0.93 to −0.7)	<0.001
Tropical Latin America	83,891 (33,548–1,79,735)	69.96 (27.96–150.81)	2,10,538 (85,873–4,38,936)	80.01 (32.66–167.02)	0.47 (0.35 to 0.59)	<0.001
Western Europe	3,30,186 (1,29,981–6,79,807)	70.07 (27.86–145.68)	3,84,372 (1,50,461–7,87,247)	61.33 (24.15–127.33)	−0.44 (−0.56 to −0.32)	<0.001
Western Sub-Saharan Africa	1,83,728 (73,840–3,65,920)	141.78 (57.31–283.61)	2,82,179 (1,13,968–5,89,754)	86.54 (34.51–176.53)	−1.58 (−1.7 to −1.46)	<0.001

AAPC, average annual percent change; ASR, age-standardized rate; DALYs, disability-adjusted life-years; GBD, Global Burden of Disease; SDI, socio-demographic index.

### 3.2 Temporal trends in the burden of periodontal diseases

Globally, the ASR of prevalence and DALYs increased marginally from 1990 to 2021, whereas the ASR of incidence remained stable (Tables 1–3). Regionally, Australasia demonstrated the greatest increases in the ASR of prevalence and DALYs, whereas North Africa and Middle East showed the greatest increases in ASR of incidence (Tables 1–3). In contrast, Oceania recorded the most substantial declines in ASR across all three metrics. Nationally, Turkey demonstrated the highest AAPC of incidence, prevalence, and DALYs, and Kiribati demonstrated the lowest AAPC for prevalence and DALYs. Spain showed the lowest AAPC for incidence (Supplementary Tables S1–S3).

**Figure 3:**
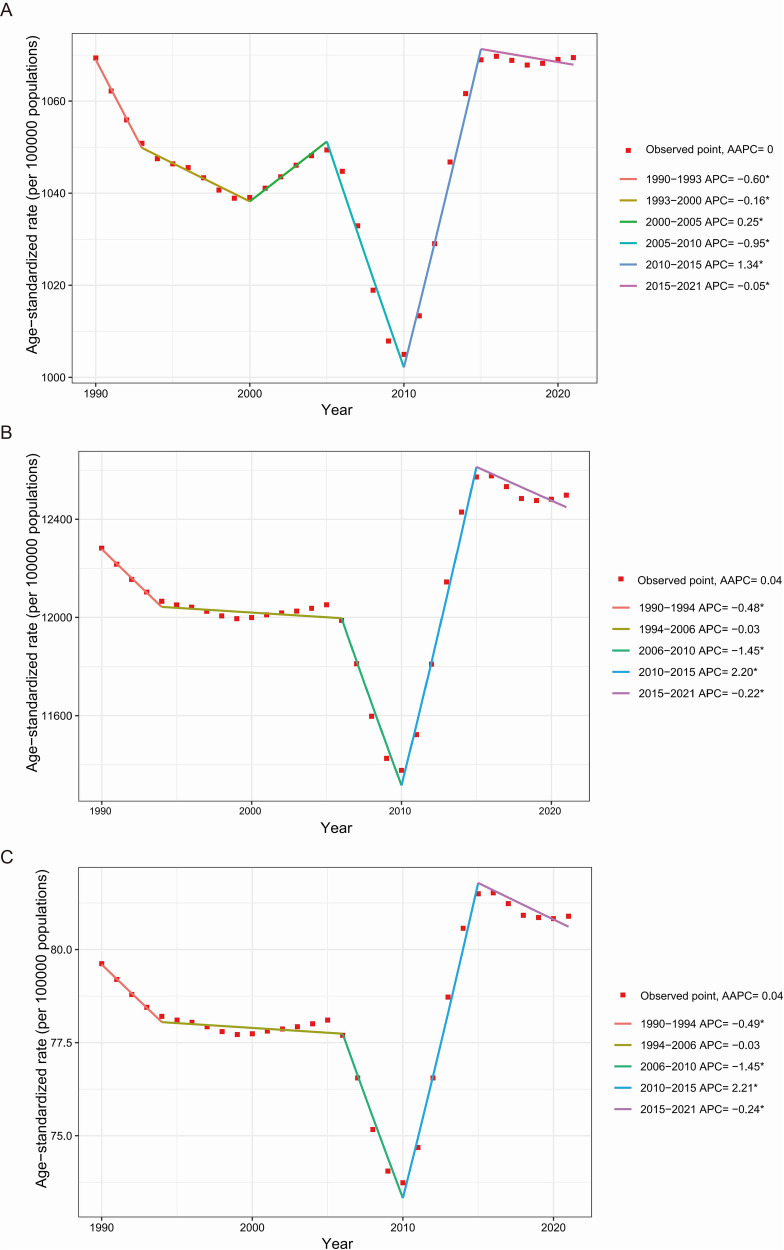
Global temporal trends of the ASR of incidence **(A)**, prevalence **(B)**, and DALYs **(C)** for periodontal diseases from 1990 to 2021. AAPC, average annual percentage change; APC, annual percent change; ASR, age-standardized rate; DALYs, disability-adjusted life-years. *p < 0.05.

The global ASR trends for periodontal diseases fluctuated significantly from 1990 to 2021, with modest overall increases and varied local trends (Figure 3). Specifically, the ASR of incidence had five joinpoints, with notable decreases from 1990 to 1993, 1993 to 2000, 2005 to 2010, and 2015 to 2021, and significant increases from 2000 to 2005 and 2010 to 2015. The ASR of prevalence and DALYs shared four joinpoints, with marked decreases from 1990 to 1994, 2006 to 2010, and 2015 to 2021, a moderate decline from 1994 to 2006, and a pronounced rise from 2010 to 2015.

### 3.3 Decomposition analysis on periodontal diseases DALYs

**Figure 4:**
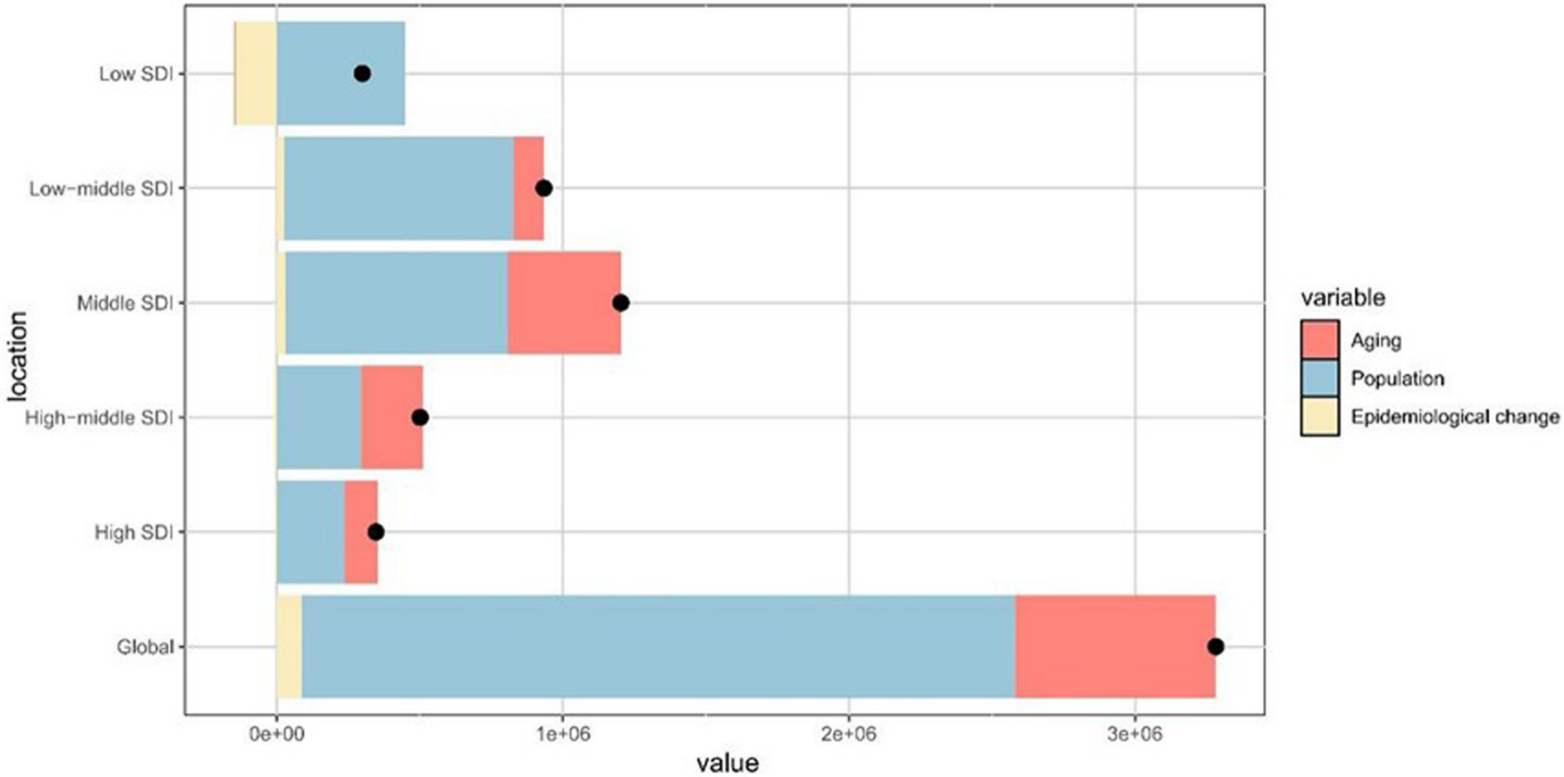
Contributions of aging, population growth, and epidemiological change to the variations in periodontal diseases DALYs from 1990 to 2021, stratified by SDI quintiles. The black dot denotes the overall effect of all three factors combined. DALYs, disability-adjusted life-years; SDI, sociodemographic index.

Globally, the DALYs related to periodontal diseases increased from 1990 to 2021, with the highest growth observed in the middle SDI regions (Figure 4). Population growth accounted for 75.98% of the global increase, followed by aging at 21.37% and epidemiological changes at 2.65%. The most substantial contributions from aging, population growth, and epidemiological change were observed in regions with high-middle, low, and low-middle SDI, respectively (Supplementary Table S4).

### 3.4 Correlation between the SDI and ASR for periodontal diseases

**Figure 5:**
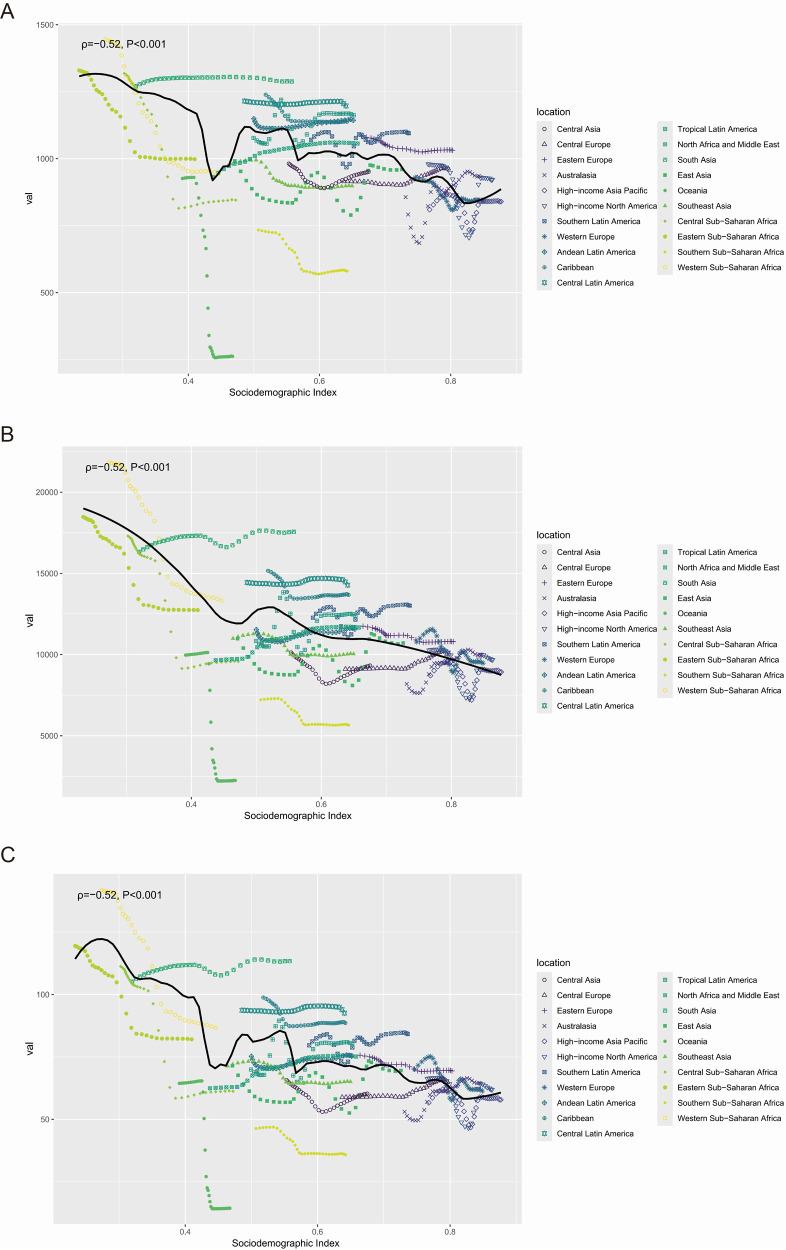
The correlation between the SDI and the ASR of incidence **(A)**, prevalence **(B)**, and DALYs **(C)** for periodontal diseases from 1990 to 2021, at the regional level. ASR, age-standardized rate; DALYs, disability-adjusted life-years; SDI, sociodemographic index.

Analysis of periodontal diseases indicators across 21 GBD regions demonstrated a negative correlation between the SDI and ASR for incidence (*ρ* = −0.52, *P* < 0.001), prevalence (*ρ* = −0.52, *P* < 0.001), and DALYs (*ρ* = −0.52, *P* < 0.001) (Figure 5).

### 3.5 Predictive analysis of the periodontal diseases burden to 2040

Globally, the case numbers of incidence, prevalence, and DALYs for periodontal diseases are projected to increase substantially by 2040, rising by 27.89%, 39.67%, and 38.06%, respectively. However, the global ASR for these metrics is anticipated to show minimal growth from 2022 to 2040, with increases of 9.37% for incidence, 10.25% for prevalence, and 9.37% for DALYs (Supplementary Figure S1, Supplementary Table S5).

## 4 Discussion

This study presents a comprehensive global assessment of the periodontal disease burden based on the latest data from GBD 2021. By integrating data encompassing all age groups across five SDI quintiles, 21 GBD regions, and 204 countries/territories from 1990 to 2021, multiple analytical methods were utilized to characterize the disease burden across different populations, periods, and geographic areas. Interestingly, periodontal diseases remain a significant global health challenge, with increasing incidence, prevalence, and DALYs. Population growth and aging are the primary drivers of this escalating burden. Notably, significant negative correlations were observed between the ASR for all metrics and the SDI across regions. Projections indicate continued growth in case numbers and a moderate annual increase in ASR through 2040. These findings provide novel insights into the epidemiology of global periodontal diseases and support evidence-based targeted public health interventions.

In 2021, periodontal diseases accounted for 89.6 million incident cases, 106.7 million prevalent cases, and 6.9 million DALYs globally, with South Asia experiencing the highest burden. South Asia encompasses numerous densely populated low- to middle-income countries. Tobacco consumption and diabetes represent the major risk factors for periodontal diseases (29, 30). The prevalence of diabetes has risen substantially in South Asia during recent decades (31). Furthermore, South Asia remains one of the largest tobacco producers worldwide (32). Results from the GBD 2015 indicated that South Asia exhibited the second-highest regional burden of smoking-related periodontal diseases (33). Its substantial population, in addition to these risk factors, may explain the elevated disease burden. Nationally, the incidence, prevalence, and DALYs of periodontal diseases demonstrated considerable variation. These differences necessitate adaptable public health policies tailored to each nation's circumstances. In 2021, Sierra Leone exhibited the highest disease burden, with the greatest ASR for incidence, prevalence, and DALYs, alongside an alarmingly high AAPC. Sierra Leone is a low-income nation in West Africa with a population exceeding 7.5 million; thus, it faces major oral healthcare challenges. Dental services remain limited, with the country reporting a critical shortage of dentists (34). These limitations likely contribute to the country's substantial periodontal disease burden. In 2021, India recorded the highest case numbers of incidence, prevalence, and DALYs related to periodontal diseases, followed by China, primarily because of their large populations (9). However, Kiribati recorded the lowest AAPC for prevalence and DALYs. Despite its low-to-middle-income status, Kiribati provides free healthcare to all residents (35). Spain demonstrated the lowest AAPC for incidence, with a rapid increase in the dental workforce over recent decades (36). Spain prioritizes early dental care and has implemented a pediatric dentistry program, which has considerably improved oral health in children. Moreover, its dental strategy presents a valuable model for other countries developing comprehensive oral healthcare systems.

From 1990 to 2021, the global ASR of prevalence and DALYs related to periodontal diseases increased, while the ASR of incidence remained stable, aligning with previous studies (13, 14). This stability in incidence suggests a consistent number of new annual cases. Nonetheless, the rise in prevalence likely reflects the chronic nature of periodontal diseases: longer life expectancy allows more individuals to live with the condition for extended periods, while cure rates and mortality remain low (37). Consequently, the prevalence continues to increase. Elevated DALYs may be attributed to increased prevalence and a growing awareness of the association between periodontal diseases and systemic conditions, such as diabetes, which lead to higher disability weight in the GBD dataset (38). While previous studies have merely described the divergent trends in incidence, prevalence, and DALYs (13, 14), to the best of our knowledge, this is the first research to provide an explanation for these differing trends. The stable ASR of incidence may mask the cumulative progression of inflammatory damage in aging populations. This cognitive bias may result in delayed clinical interventions. To address this subtle yet critical challenge, dentists should emphasize early screening and diagnosis of periodontal diseases in older adults, particularly in those with irreversible complications, such as tooth mobility and alveolar bone resorption.

When analyzing periodontal disease trends from 1990 to 2021, major turning points emerged, particularly in 2005 (or 2006), 2010, and 2015. After 2005, the ASR of incidence, prevalence, and DALYs for periodontal diseases substantially declined. This trend aligns with the application of the World Health Organization Framework Convention on Tobacco Control (WHO FCTC), the first international public health treaty on tobacco control, which came into effect in 2005 (39). The implementation of tobacco demand-reduction measures outlined in the WHO FCTC led to decreased tobacco consumption and a decline in associated oral health problems (40). Conversely, the ASR for incidence, prevalence, and DALYs of periodontal diseases increased significantly from 2010 to 2015. This increase potentially reflects the rising prevalence of risk factors for periodontal diseases. Tobacco smoking, diabetes, obesity, poor nutrition, and physical inactivity enhance the risk of periodontal diseases (41). The increasing prevalence of these factors, particularly obesity and diabetes in developing nations, exacerbates the disease burden. Notably, from 2015 to 2021, a moderate decrease was observed in ASR across all three measures. Several factors contributed to this trend, such as improved oral health awareness, government initiatives (such as Healthy China 2030) (42), and technological advancements in dental healthcare. Compared with studies focusing on individual countries or regions (9, 17), this research pioneers the identification of global turning points in periodontal diseases burden. These findings offer important practical implications. Policymakers may utilize burden changes reflected at these points to enhance intervention strategies, including integrated tobacco control and oral healthcare, and to strengthen the co-management of chronic diseases and oral health. For resource allocation, the precise detection of inflection points enables targeted investment during high-burden phases. Additionally, dynamic monitoring of these points facilitates predicting future trends and providing scientific support for evidence-based planning and global resource optimization to advance the WHO's Global Oral Health Action Plan 2023 to 2030 (43).

These findings demonstrated a significant negative correlation between the SDI and ASR for incidence, prevalence, and DALYs related to periodontal diseases, suggesting broader socioeconomic influences. However, as an ecological analysis, causal inferences could not be established because of unmeasured confounding and the aggregate nature of data. The observed gradient likely originates from disparities in oral healthcare access: populations with high SDI receive early diagnosis through regular dental visits, whereas their low SDI counterparts frequently present with advanced disease symptoms, such as bleeding gums, tooth mobility, or tooth loss, owing to limited access to early care (44, 45). These disparities necessitate policies addressing social and economic barriers to oral health (46). In low-SDI regions, health systems should emphasize expanding insurance coverage, incorporating dental services into primary healthcare, and training community health workers in oral disease prevention. Addressing technological disparities and enhancing oral hygiene education in underserved areas remains essential. A life-course approach and enhanced screening can facilitate earlier detection and minimize the long-term impacts of periodontal diseases. These interventions may improve periodontal diseases prevention and control, particularly in underserved regions, and advance global equity in oral health.

The decomposition analysis suggested that population growth and aging primarily contributed to the increased burden of periodontal diseases from 1990 to 2021. According to the United Nations, the global population will continue to expand, with the population aged ≥65 increasing at a faster rate than younger age groups (https://www.un.org/en/global-issues/population). Considering the well-established relationship between aging and periodontal diseases, healthcare systems are required to better serve the needs of older adults, including specialized geriatric oral health services (47, 48). Concurrently, oral disease prevention remains crucial. Essential strategies include promoting oral hygiene, managing risk factors, promoting regular plaque removal, and increasing access to comprehensive screening programs (49). Interestingly, in regions with low, high-middle, and high SDI, epidemiological changes exhibited a protective effect, partially offsetting the impact of population growth and aging. A comparable inverted U-shaped trend has been documented in the burden of cardiovascular disease, indicating a temporary protective effect in early stages (50). Moreover, this trend may indicate the delayed saturation of behavioral risks, such as high sugar consumption and tobacco use, which typically increase with socioeconomic development. However, this protective window is limited. Rapid dietary changes and inadequate oral health infrastructure may reverse this progress. To maintain positive trends, periodontal screening should be integrated into existing non-communicable disease frameworks to minimize diagnostic delays and improve access to care.

The anticipated increase in the global burden of periodontal disease by 2040—characterized by moderate rises in ASR but significant growth in case numbers—reflects the combined impact of population aging, the chronic nature of periodontal diseases, and disparate access to care. These findings correspond with the dental demand model proposed by Manski et al., which identifies the rapid expansion of the population aged ≥65 years as a crucial determinant of dental service utilization (51). This demographic shift explains the projected 25% increase in dental care seekers by 2040 (compared with 2015), despite stable ASR (51). Technological advances may reduce treatment needs in early-stage disease; however, they might increase the complexity of managing advanced disease, a factor often neglected in conventional models. The analysis using DALYs suggested that increasing disease severity may counteract these gains in treatment efficiency. Insurance coverage limitations, including the exclusion of adult dental benefits under the Affordable Care Act, continue to restrict access to care services (51). Compared with recent studies focusing on disease burden up to 2035 or 2050 (14, 15), these projections through 2040 address the critical period after the WHO's Global Oral Health Goals 2030 (43). This projection provides vital evidence for policymaking and fills a major knowledge gap, emphasizing policy reform. Specifically, insurance must support sustained periodontal maintenance, and not episodic care, to convert increased service utilization into meaningful reductions in disease burden.

This study has several limitations. First, although the GBD 2021 assessed the burden of periodontal diseases across 204 countries/territories, the raw data did not cover all locations. Limited raw data coverage may affect the precision of disease burden estimates, particularly in areas with limited surveillance. Furthermore, variations in diagnostic criteria and data quality across countries introduce measurement bias. Second, the definition of periodontal diseases in the GBD lacked standardization. Varying definitions may affect diagnosis and case estimation. Additionally, the exclusion of edentulism and periodontal disease sequelae from DALY calculations potentially underestimates the actual disease burden. Third, underreporting or misreporting in low-income regions may result in underascertainment of periodontal disease cases, thus underestimating disease burden and concealing the actual prevalence. Finally, this study quantified trends in the burden of periodontal diseases without analyzing specific risk factors. Future research should address these limitations by incorporating individual-level risk factors, implementing standardized diagnostic criteria, and enhancing data quality and geographic coverage to provide more robust evidence for prevention and control strategies.

## 5 Conclusion

Periodontal diseases continue to pose a substantial public health challenge globally. Decomposition analysis of data from 1990 to 2021 identified population growth and aging as the primary drivers of increasing disease burden, demonstrated by significant increases in incidence, prevalence, and DALYs, despite moderate ASR trends. Projections through 2040 suggest an accelerating trend—characterized by modest increases in ASR but substantially expanding case numbers—reflecting the combined effects of demographic changes, disease chronicity, and persistent healthcare disparities. Without systematic interventions, this growth may exceed existing healthcare capacities, exacerbating health inequalities. Comprehensive prevention strategies, particularly those targeting countries with low to middle SDI, are crucial to mitigate this burden. Future research should incorporate dynamic factors, including dietary transitions, climate impacts, and policy reforms, into GBD frameworks to improve predictive accuracy. Addressing these trends requires integrating oral health into universal health coverage to ensure equitable access to care and innovations.

## Data Availability

Publicly available datasets were analyzed in this study. This data can be found here: https://vizhub.healthdata.org/gbd-results/.
