# Supplementary material for: Global, regional, and national burden of periodontal diseases from 1990 to 2021 and predictions to 2040: an analysis of the global burden of disease study 2021
Source: Front Oral Health. 2025 Jul 24;6:1627746. doi: 10.3389/froh.2025.1627746 (PMC12332980; doi:10.3389/froh.2025.1627746)
Supplement: Supplementary file 1 [file Supplementaryfile1.docx]

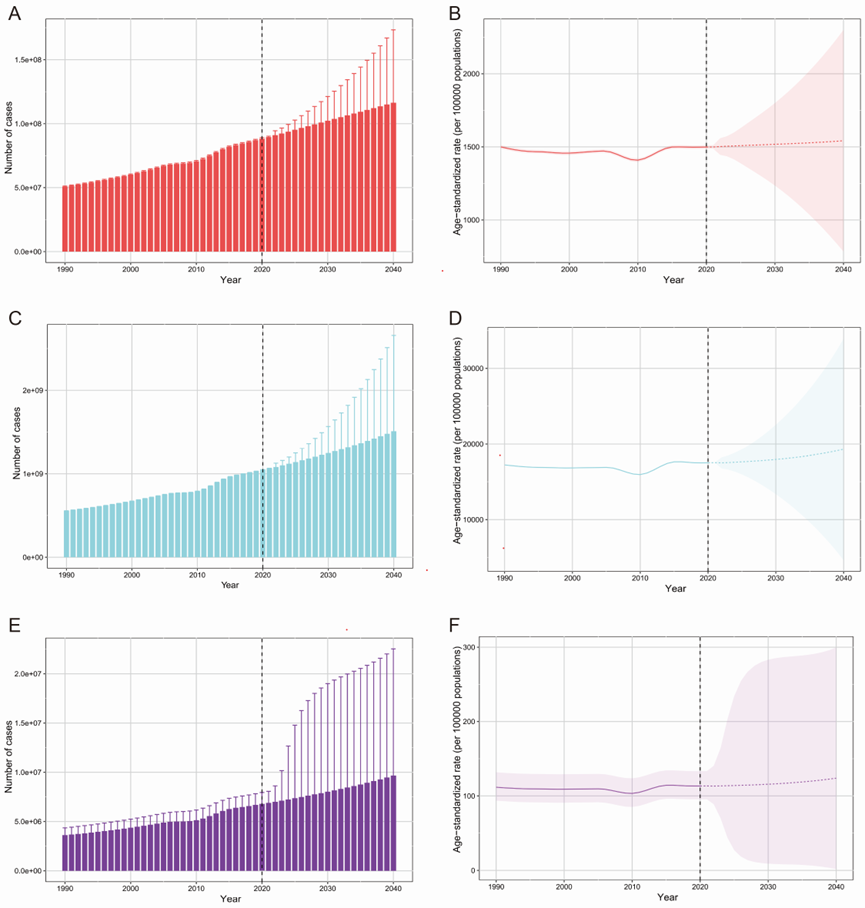


**Supplementary Figure S1** Projected global case numbers and ASR of periodontal diseases to 2040. (A) Projected case number of incidence; (B) Projected ASR of incidence; (C) Projected case number of prevalence; (D) Projected ASR of prevalence; (E) Projected case number of DALYs; (F) Projected ASR of DALYs. ASR, age-standardized rate; DALYs, disability-adjusted life-years.
