# Supplementary material for: Global, regional, and national burden of periodontal diseases from 1990 to 2021 and predictions to 2040: an analysis of the global burden of disease study 2021
Source: Front Oral Health. 2025 Jul 24;6:1627746. doi: 10.3389/froh.2025.1627746 (PMC12332980; doi:10.3389/froh.2025.1627746)
Supplement: Supplementary file 2 [file Table1.docx]

**Supplementary Table S1** The case number and ASR of incidence of periodontal diseases in 1990 and 2021 across 204 countries and territories, with AAPC from 1990 to 2021

| **Location** | **1990** | | **2021** | | **AAPC (95%CI)**  **1990–2021** | ***P*** |
| --- | --- | --- | --- | --- | --- | --- |
|  | **Number (95%UI)** | **ASR (95%UI)** | **Number (95%UI)** | **ASR (95%UI)** |  |  |
| Afghanistan | 58125  (42092-72310) | 803  (590.47-996.24) | 159107  (116546-213235) | 854.25  (654.22-1073.22) | 0.19 (0.17 to 0.22) | <0.001 |
| Albania | 21227  (15481-26713) | 807.63  (597.05-1003.55) | 30591  (24585-37058) | 884.05  (682.25-1080.32) | 0.3 (0.28 to 0.33) | <0.001 |
| Algeria | 17608  (148536-210103) | 1028.92  (895.31-1180.28) | 470687  (374942-577966) | 1069.69  (873.2-1285.69) | 0.12 (0.11 to 0.13) | <0.001 |
| American Samoa | 400  (308-485) | 1117.95  (908.31-1294.44) | 547  (436-632) | 1087.86  (868.36-1267.61) | -0.09 (-0.09 to -0.08) | <0.001 |
| Andorra | 625  (523-726) | 979.3  (826.39-1129.55) | 1127  (877-1375) | 911.29  (707.2-1135.15) | -0.24 (-0.28 to -0.19) | <0.001 |
| Angola | 103220  (77535-123427) | 1360.46  (1087.9-1558.35) | 213603  (161374-289885) | 1010.99  (795.61-1266.93) | -0.95 (-1.01 to -0.9) | <0.001 |
| Antigua and Barbuda | 771  (601-900) | 1323.74  (1068.69-1512.83) | 1294  (1100-1485) | 1242.75  (1057.49-1433.85) | -0.2 (-0.24 to -0.15) | <0.001 |
| Argentina | 332144  (252469-398306) | 1039.79  (796.34-1247.16) | 530937  (423142-646205) | 1047.89  (830.57-1285.21) | 0.05 (0 to 0.11) | 0.071 |
| Armenia | 30287  (23439-36393) | 965.59  (761.06-1147.25) | 35692  (29206-42782) | 955.08  (763.07-1159.14) | -0.03 (-0.06 to -0.01) | 0.004 |
| Australia | 157654  (117890-194804) | 843.18  (629.11-1035.07) | 286453  (232291-352824) | 879.69  (691.66-1105.01) | 0.12 (-0.06 to 0.3) | 0.175 |
| Austria | 86413  (65890-106544) | 914.66  (675.92-1133.81) | 108015  (86614-132041) | 862.85  (663.01-1073.05) | -0.19 (-0.22 to -0.16) | <0.001 |
| Azerbaijan | 61028  (47740-73891) | 1008.13  (798.28-1199.38) | 119102  (95371-144678) | 996.94  (805.87-1200.09) | -0.04 (-0.07 to -0.01) | 0.007 |
| Bahamas | 3291  (2597-3867) | 1356.43  (1117.31-1540.41) | 5442  (4627-6274) | 1258.35  (1073.74-1450.95) | -0.24 (-0.26 to -0.22) | <0.001 |
| Bahrain | 4846  (3337-6174) | 1107.79  (868.7-1297.79) | 20945  (17030-25540) | 1175.19  (991.05-1395.05) | 0.19 (0.19 to 0.2) | <0.001 |
| Bangladesh | 1073902  (794616-1296439) | 1314.21  (1028.65-1522.77) | 2188618  (1817932-2520902) | 1313.1  (1106.11-1499.24) | -0.01 (-0.03 to 0.02) | 0.589 |
| Barbados | 3516  (2819-4080) | 1313.69  (1062.05-1500.75) | 4437  (3784-5128) | 1205.84  (1000.22-1414.31) | -0.28 (-0.3 to -0.26) | <0.001 |
| Belarus | 119785  (95862-141023) | 1011.61  (790.02-1199.62) | 125138  (103939-148554) | 994.49  (809.48-1201.63) | -0.04 (-0.07 to -0.02) | 0.003 |
| Belgium | 151850  (125134-172508) | 1284.52  (1038.14-1478.84) | 178664  (158558-200991) | 1255.13  (1088.7-1450.52) | -0.07 (-0.1 to -0.04) | <0.001 |
| Belize | 1601  (1197-1934) | 1208.02  (957.49-1403.28) | 4688  (3799-5746) | 1147.05  (947.29-1366.1) | -0.17 (-0.18 to -0.15) | <0.001 |
| Benin | 51981  (41554-59355) | 1463.35  (1204.19-1631.18) | 132187  (103940-161086) | 1302.23  (1073.55-1504.69) | -0.37 (-0.41 to -0.34) | <0.001 |
| Bermuda | 944  (761-1065) | 1371.11  (1118.66-1544.45) | 1085  (936-1210) | 1388.55  (1133.95-1562.5) | 0.04 (0.03 to 0.05) | <0.001 |
| Bhutan | 6727  (4862-8227) | 1349.65  (1044.61-1558.99) | 11094  (9316-12778) | 1364.8  (1172.38-1549.02) | 0.04 (0.03 to 0.05) | <0.001 |
| Bolivia (Plurinational State of) | 52352  (40149-62959) | 1126.48  (902.43-1322.12) | 124456  (100524-153298) | 1099.9  (897.91-1326.46) | -0.08 (-0.09 to -0.07) | <0.001 |
| Bosnia and Herzegovina | 33231  (23855-42723) | 717.12  (519.24-908.62) | 41306  (33220-50110) | 887.4  (691.3-1088.36) | 0.7 (0.65 to 0.75) | <0.001 |
| Botswana | 4491  (3060-5915) | 567.3  (400.08-748.4) | 9378  (6603-12962) | 446.7  (332.94-608.9) | -0.73 (-0.85 to -0.6) | <0.001 |
| Brazil | 1315254  (1009489-1555726) | 1077.07  (868.22-1252.19) | 2909488  (2516865-3230573) | 1164.41  (998.65-1300.77) | 0.27 (0.21 to 0.32) | <0.001 |
| Brunei Darussalam | 1829  (1341-2278) | 947.46  (756.65-1122.8) | 4105  (3113-5218) | 840.54  (664.02-1026.44) | -0.38 (-0.42 to -0.35) | <0.001 |
| Bulgaria | 81150  (58773-103338) | 736.94  (530.8-933.73) | 77066  (60726-95489) | 749.56  (569.05-948.15) | 0.07 (0 to 0.14) | 0.05 |
| Burkina Faso | 99561  (78630-116168) | 1439.14  (1163.19-1627.85) | 259812  (206731-301694) | 1442.58  (1184.81-1622.71) | 0.01 (0 to 0.01) | 0.192 |
| Burundi | 53686  (39452-64889) | 1336.18  (1043.68-1542.58) | 64345  (45688-88315) | 757.5  (570.01-986.75) | -1.79 (-1.95 to -1.64) | <0.001 |
| Cabo Verde | 4248  (3448-4864) | 1476.78  (1212.31-1635.89) | 8960  (7453-9878) | 1510.84  (1283.37-1662.22) | 0.07 (0.06 to 0.08) | <0.001 |
| Cambodia | 50582  (36126-64549) | 796.71  (586.28-1003.95) | 144879  (107300-181370) | 922.45  (694.61-1128.26) | 0.48 (0.45 to 0.5) | <0.001 |
| Cameroon | 121729  (99592-136196) | 1494.07  (1238.99-1652.45) | 396745  (319578-449477) | 1476.92  (1213.79-1644.16) | -0.04 (-0.05 to -0.02) | <0.001 |
| Canada | 365541  (293593-422776) | 1171.38  (946.21-1348.43) | 562031  (496407-635995) | 1175.61  (1012.16-1386.48) | 0.01 (-0.01 to 0.03) | 0.238 |
| Central African Republic | 25266  (18936-30393) | 1255.49  (990.6-1460.51) | 28064  (20067-38265) | 750.7  (574.9-978.78) | -1.66 (-1.76 to -1.57) | <0.001 |
| Chad | 64590  (50706-75312) | 1441.69  (1156.13-1631.1) | 150186  (117372-186979) | 1280.53  (1054.67-1499.07) | -0.39 (-0.44 to -0.34) | <0.001 |
| Chile | 135469  (103115-162502) | 1091.93  (853.91-1290.56) | 258002  (214986-302300) | 1171.55  (967.2-1371.55) | 0.17 (0.04 to 0.3) | 0.009 |
| China | 10605758  (8290110-12656825) | 998.49  (791.22-1179.28) | 18295038  (15747998-20729598) | 967.03  (830.95-1100.24) | -0.09 (-0.2 to 0.02) | 0.101 |
| Colombia | 324042  (246396-386409) | 1194.36  (950.46-1383.41) | 650364  (538551-756704) | 1196.53  (991.6-1393.14) | 0 (-0.01 to 0.02) | 0.525 |
| Comoros | 4838  (3790-5754) | 1408.9  (1141.17-1595.05) | 5974  (4359-7774) | 909.94  (675.85-1156.49) | -1.39 (-1.51 to -1.26) | <0.001 |
| Congo | 25116  (18969-29911) | 1374.1  (1086.08-1562.68) | 43924  (32657-56529) | 987.22  (773.91-1234) | -1.04 (-1.16 to -0.92) | <0.001 |
| Cook Islands | 166  (130-197) | 1073.71  (867.29-1258.65) | 86  (63-118) | 378.75  (283.7-509.49) | -3.32 (-3.44 to -3.21) | <0.001 |
| Costa Rica | 30471  (23252-36315) | 1203.11  (959.21-1394.05) | 64576  (54168-75630) | 1207.31  (1014.53-1410.29) | 0.01 (0 to 0.02) | 0.034 |
| Coted'Ivoire | 144148  (116558-162633) | 1492.56  (1239.66-1654.11) | 303856  (242922-362521) | 1341.28  (1123.6-1537.05) | -0.35 (-0.39 to -0.31) | <0.001 |
| Croatia | 60689  (54491-66596) | 1034.85  (924.5-1138.61) | 64400  (56471-73118) | 1131.25  (959.06-1345.13) | 0.29 (0.24 to 0.33) | <0.001 |
| Cuba | 143866  (112531-169133) | 1265.62  (1015.22-1463.77) | 164199  (139451-190895) | 1164.32  (972.75-1384.76) | -0.26 (-0.3 to -0.22) | <0.001 |
| Cyprus | 7355  (5409-9146) | 898.61  (667.31-1111.48) | 14951  (11749-18725) | 833.87  (638.3-1052.95) | -0.25 (-0.32 to -0.18) | <0.001 |
| Czechia | 117224  (90971-140135) | 964.87  (742.93-1155.38) | 145499  (120925-169976) | 981.07  (785.15-1191.38) | 0.07 (0.01 to 0.12) | 0.02 |
| Democratic People's Republic of Korea | 141558  (104349-179298) | 741.54  (563.35-919.32) | 226481  (164577-292238) | 675.8  (494.7-863.58) | -0.3 (-0.32 to -0.27) | <0.001 |
| Democratic Republic of the Congo | 356656  (264049-432237) | 1304.08  (1017.02-1518.15) | 470272  (334741-638747) | 774.85  (584.06-1015.58) | -1.67 (-1.76 to -1.57) | <0.001 |
| Denmark | 91572  (80491-99921) | 1541.67  (1339.93-1698.34) | 102233  (92710-110966) | 1495.38  (1340.18-1638.65) | -0.1 (-0.13 to -0.07) | <0.001 |
| Djibouti | 4800  (3684-5774) | 1432.61  (1153.84-1654.61) | 10668  (7687-14258) | 965.67  (736.21-1216.97) | -1.26 (-1.3 to -1.21) | <0.001 |
| Dominica | 786  (608-933) | 1248.79  (987.85-1457.97) | 890  (741-1039) | 1177.1  (982.02-1384.78) | -0.19 (-0.22 to -0.16) | <0.001 |
| Dominican Republic | 69966  (52523-83892) | 1220.04  (965.65-1421.52) | 135974  (113788-159859) | 1207.27  (1018.14-1400.02) | -0.03 (-0.08 to 0.02) | 0.202 |
| Ecuador | 90601  (76334-104878) | 1170.09  (1024.5-1311.18) | 205728  (169785-246607) | 1145.48  (954.6-1358.65) | -0.07 (-0.08 to -0.05) | <0.001 |
| Egypt | 358008  (258739-442898) | 903.28  (676.7-1103.56) | 952238  (745001-1195066) | 1046.75  (854.55-1273.4) | 0.47 (0.44 to 0.51) | <0.001 |
| El Salvador | 43941  (37249-51090) | 1114.58  (967.68-1268.24) | 72722  (59691-88102) | 1142.34  (941.83-1368.24) | 0.08 (0.07 to 0.09) | <0.001 |
| Equa torial Guinea | 3934  (2969-4766) | 1303.65  (1029.45-1513.14) | 12908  (9809-16873) | 1139.11  (928.97-1370.67) | -0.41 (-0.58 to -0.23) | <0.001 |
| Eritrea | 32605  (24259-39900) | 1333.47  (1054.04-1540.54) | 42636  (30591-58122) | 861.92  (644.47-1104.19) | -1.47 (-1.63 to -1.3) | <0.001 |
| Es tonia | 18626  (14893-21795) | 1020.11  (800.43-1209.83) | 18394  (15869-21569) | 1039.52  (868.59-1248.05) | 0.06 (0.04 to 0.09) | <0.001 |
| Eswatini | 2421  (1658-3137) | 548.09  (383.29-715.93) | 5260  (3610-7033) | 618.56  (432.11-809.44) | 0.39 (0.37 to 0.4) | <0.001 |
| Ethiopia | 447955  (338106-535711) | 1293.02  (1024.69-1484.29) | 1123583  (850579-1346830) | 1308.36  (1040.27-1501.98) | 0.04 (0.03 to 0.05) | <0.001 |
| Fiji | 5645  (4234-6871) | 988.72  (766.86-1173.49) | 2562  (1851-3555) | 302.17  (222.62-412.33) | -3.75 (-3.88 to -3.62) | <0.001 |
| Finland | 66882  (52531-78159) | 1126.53  (878-1319.89) | 83612  (72797-95547) | 1164.21  (991.64-1374.13) | 0.12 (0.07 to 0.17) | <0.001 |
| France | 436407  (354316-530635) | 635.39  (508.58-776.34) | 632602  (486142-806137) | 687.94  (523.81-902.69) | 0.28 (0.16 to 0.4) | <0.001 |
| Gabon | 11800  (9530-13699) | 1459.7  (1212.68-1640.86) | 16859  (12953-21366) | 1101.8  (881.45-1323.29) | -0.91 (-0.99 to -0.82) | <0.001 |
| Gambia | 12065  (10163-13411) | 1538.32  (1316.59-1680.95) | 32070  (26975-35530) | 1529.52  (1324.17-1676.61) | -0.02 (-0.03 to -0.01) | 0.001 |
| Georgia | 60843  (48893-71218) | 1022.65  (811.39-1195.93) | 44285  (36759-52992) | 965.93  (778.09-1180.08) | -0.19 (-0.21 to -0.16) | <0.001 |
| Germany | 1253149  (1096370-1376066) | 1289.05  (1133.29-1418.27) | 1423951  (1267056-1591216) | 1306.38  (1136.52-1493.11) | -0.02 (-0.23 to 0.19) | 0.871 |
| Ghana | 178333  (141695-202319) | 1475.98  (1207.62-1641.58) | 466581  (381053-522767) | 1496.75  (1244.76-1659.71) | 0.04 (0.04 to 0.05) | <0.001 |
| Greece | 102543  (84092-121070) | 817.97  (660.75-980.37) | 124371  (98721-152714) | 837.47  (648.65-1057.26) | 0.09 (0.05 to 0.14) | <0.001 |
| Greenland | 547  (402-663) | 1012.8  (781.76-1200.85) | 677  (545-815) | 1013.47  (827.11-1221.41) | 0 (-0.02 to 0.03) | 0.795 |
| Grenada | 874  (677-1033) | 1230.97  (973.92-1421.14) | 1364  (1127-1588) | 1199.38  (994.86-1404.75) | -0.08 (-0.1 to -0.06) | <0.001 |
| Guam | 1431  (1085-1712) | 1161.57  (945.6-1339.86) | 786  (576-1073) | 407.99  (303.42-549.97) | -3.28 (-3.52 to -3.05) | <0.001 |
| Guatemala | 61050  (45911-73577) | 1133.01  (890.24-1334.48) | 161991  (130288-200028) | 1136.14  (937.73-1361.58) | 0.01 (-0.01 to 0.02) | 0.272 |
| Guinea | 68444  (55137-77452) | 1468.09  (1188.87-1639.8) | 156456  (122723-180355) | 1453.2  (1177.5-1626.24) | -0.03 (-0.05 to -0.02) | <0.001 |
| Guinea-Bissau | 11106  (8744-12865) | 1451.06  (1169.73-1633.5) | 20014  (15400-24979) | 1261.83  (1029.11-1482.02) | -0.45 (-0.49 to -0.4) | <0.001 |
| Guyana | 7362  (5575-8906) | 1184.59  (928.18-1378.09) | 8734  (6984-10582) | 1156.57  (944.97-1386.81) | -0.07 (-0.12 to -0.03) | 0.001 |
| Haiti | 53376  (39358-64500) | 1126.93  (879.66-1334.04) | 110077  (81255-144244) | 982.03  (753.41-1226.11) | -0.43 (-0.47 to -0.4) | <0.001 |
| Honduras | 34108  (25160-41534) | 1108.64  (857.23-1315.29) | 97616  (77683-125453) | 1095.87  (893.61-1323.62) | -0.04 (-0.05 to -0.03) | <0.001 |
| Hungary | 63257  (43318-84278) | 481.92  (332.61-639.72) | 76407  (55489-100914) | 515.26  (388.46-696.67) | 0.21 (0.14 to 0.28) | <0.001 |
| Iceland | 2394  (2002-2800) | 900.49  (749.85-1051.95) | 3776  (2985-4608) | 859.91  (660.8-1080.05) | -0.15 (-0.16 to -0.13) | <0.001 |
| India | 8814875  (6779093-10411275) | 1247.98  (990.44-1438.59) | 18527946  (16166600-20711595) | 1268.96  (1119.16-1409.94) | 0.05 (0.04 to 0.06) | <0.001 |
| Indonesia | 1686126  (1267400-2014477) | 1136.02  (899.42-1322.27) | 3442904  (2713509-4006775) | 1148.61  (912.49-1334.29) | 0.04 (0.03 to 0.05) | <0.001 |
| Iran (Islamic Republic of) | 418029  (315251-501272) | 1098.73  (873.89-1286.6) | 1059260  (873577-1216465) | 1097.07  (912.65-1249.49) | -0.01 (-0.03 to 0) | 0.132 |
| Iraq | 115509  (85025-142482) | 993.92  (775.6-1188.96) | 389248  (304792-498569) | 1076.54  (877.49-1316.77) | 0.26 (0.23 to 0.28) | <0.001 |
| Ireland | 18277  (12603-24003) | 499.86  (347.34-653.61) | 36166  (27160-47356) | 566.85  (424.21-758.54) | 0.37 (0.28 to 0.46) | <0.001 |
| Israel | 41854  (31023-52241) | 897.43  (665.05-1109.82) | 85369  (67804-106363) | 833.6  (644.6-1048.29) | -0.23 (-0.29 to -0.18) | <0.001 |
| Italy | 658504  (512245-790159) | 926.86  (708.58-1123.14) | 793110  (649947-958076) | 885.37  (695.81-1088.83) | -0.15 (-0.16 to -0.14) | <0.001 |
| Jamaica | 24859  (19172-29968) | 1238.02  (987.61-1444.12) | 36138  (29872-43376) | 1158.2  (960.34-1384.08) | -0.22 (-0.29 to -0.14) | <0.001 |
| Japan | 1327988  (1003304-1613177) | 842.96  (644.28-1021.07) | 1741942  (1446820-2046352) | 869.15  (690.91-1051.89) | 0.09 (0.02 to 0.16) | 0.016 |
| Jordan | 21833  (15867-27136) | 961.27  (740.51-1166.02) | 118474  (92184-148584) | 1043.01  (841.53-1248.27) | 0.26 (0.23 to 0.29) | <0.001 |
| Kazakhstan | 152288  (118288-180845) | 1029.37  (811.93-1215.32) | 202919  (162336-245662) | 1025.14  (833.28-1241.38) | -0.02 (-0.04 to 0.01) | 0.193 |
| Kenya | 222704  (166864-270111) | 1373.06  (1085.22-1567.51) | 550327  (452035-649551) | 1312.47  (1121.13-1484.77) | -0.14 (-0.15 to -0.13) | <0.001 |
| Kiribati | 455  (335-564) | 880.66  (662.7-1072.76) | 200  (144-277) | 224.21  (167.35-310.61) | -4.29 (-4.8 to -3.78) | <0.001 |
| Kuwait | 17605  (12674-21951) | 1157.59  (935.12-1354.02) | 68870  (55785-82896) | 1201.26  (1032.75-1407.44) | 0.12 (0.1 to 0.14) | <0.001 |
| Kyrgyzstan | 32677  (25102-39760) | 957.59  (747.73-1151.67) | 52534  (39265-68201) | 864.26  (674.02-1081.96) | -0.34 (-0.37 to -0.31) | <0.001 |
| Lao People's Democratic Republic | 10541  (7289-13843) | 404.92  (280.29-538.12) | 33987  (23602-45471) | 551.5  (389.7-730.02) | 1.03 (0.95 to 1.12) | <0.001 |
| Latvia | 32287  (25982-37861) | 1030.02  (810.16-1219.28) | 26465  (22547-30909) | 1028.41  (852.46-1240.45) | 0 (-0.02 to 0.02) | 0.729 |
| Lebanon | 25015  (19020-30144) | 984.42  (755.75-1179.53) | 66045  (54098-82189) | 1074.8  (881.11-1304.91) | 0.28 (0.27 to 0.3) | <0.001 |
| Lesotho | 4301  (2969-5607) | 423.69  (293.27-553.76) | 7392  (5015-9593) | 512.08  (355.93-669) | 0.61 (0.59 to 0.63) | <0.001 |
| Liberia | 28227  (22409-32342) | 1457.38  (1170.81-1635.74) | 66810  (51034-78228) | 1415.51  (1114.2-1615.19) | -0.1 (-0.11 to -0.08) | <0.001 |
| Libya | 32564  (27122-38473) | 1130.12  (978.7-1290.03) | 78145  (60821-97233) | 1033.35  (842.04-1245.69) | -0.29 (-0.31 to -0.28) | <0.001 |
| Lithuania | 42411  (33991-49793) | 1024.85  (811.34-1206.56) | 38870  (33403-45295) | 1039.65  (865.54-1257.67) | 0.05 (0.01 to 0.09) | 0.007 |
| Luxembourg | 4578  (3453-5580) | 973.71  (727.64-1189.93) | 7970  (6327-9654) | 931.42  (730.67-1141.2) | -0.13 (-0.18 to -0.08) | <0.001 |
| Madagascar | 120473  (90824-146249) | 1366.56  (1076.54-1586.08) | 69951  (49219-95717) | 374.07  (273.01-503.79) | -4.12 (-4.27 to -3.97) | <0.001 |
| Malawi | 94540  (69456-115292) | 1327.87  (1035.38-1535.85) | 103937  (75828-141717) | 810.78  (616.32-1050.17) | -1.53 (-1.58 to -1.49) | <0.001 |
| Malaysia | 143332  (104178-177773) | 1037.94  (809.36-1242.69) | 261710  (203345-322841) | 810.08  (640.83-982.29) | -0.81 (-0.92 to -0.7) | <0.001 |
| Maldives | 1320  (980-1625) | 980.72  (752.61-1175.8) | 4097  (2935-5370) | 770.79  (599.33-946.21) | -0.77 (-0.84 to -0.7) | <0.001 |
| Mali | 93267  (71658-108510) | 1431.57  (1123.31-1621.93) | 267084  (209622-307403) | 1454.2  (1180.43-1626.39) | 0.05 (0.04 to 0.06) | <0.001 |
| Malta | 3568  (2611-4449) | 858.62  (637.23-1065.05) | 5327  (4260-6462) | 846.44  (651.03-1051.46) | -0.04 (-0.07 to 0) | 0.046 |
| Marshall Islands | 232  (169-291) | 920.59  (700.48-1114.89) | 111  (79-151) | 249.39  (186.62-335.84) | -4.11 (-4.33 to -3.88) | <0.001 |
| Mauritania | 24487  (19739-27717) | 1483.5  (1226.66-1646.15) | 46934  (37983-56264) | 1339.19  (1132.56-1543.51) | -0.33 (-0.36 to -0.3) | <0.001 |
| Mauritius | 10119  (7600-12563) | 1024.47  (782.36-1237.34) | 13501  (10752-16511) | 792.8  (622.69-978.76) | -0.83 (-0.89 to -0.76) | <0.001 |
| Mexico | 824621  (631781-982299) | 1242.38  (992.01-1430.96) | 1668146  (1473029-1854971) | 1221.41  (1083.76-1359.09) | -0.05 (-0.06 to -0.05) | <0.001 |
| Micronesia (Federated States of) | 582  (428-723) | 903.87  (677.01-1089.02) | 210  (152-291) | 245.53  (183.02-336.5) | -4.12 (-4.28 to -3.97) | <0.001 |
| Monaco | 475  (384-547) | 1146.43  (905.54-1347.14) | 561  (472-657) | 1049.65  (850.17-1274.21) | -0.27 (-0.33 to -0.21) | <0.001 |
| Mongolia | 12650  (9416-15669) | 913.8  (697.49-1105.64) | 30211  (23105-37885) | 953.69  (759.9-1164.08) | 0.14 (0.12 to 0.16) | <0.001 |
| Montenegro | 5966  (4585-7161) | 918.7  (700.2-1104.14) | 7371  (5926-8808) | 921.09  (724.87-1135.11) | 0.01 (0 to 0.03) | 0.077 |
| Morocco | 254146  (192692-304803) | 1258.77  (1005.25-1451.93) | 475633  (406024-553067) | 1234.55  (1060.57-1435.63) | -0.05 (-0.06 to -0.04) | <0.001 |
| Mozambique | 120009  (89437-144696) | 1257.06  (964.37-1469.85) | 158538  (115494-215091) | 823.72  (627.38-1057.25) | -1.34 (-1.44 to -1.23) | <0.001 |
| Myanmar | 237621  (171912-305132) | 789.79  (577.1-1002.65) | 372689  (279451-472908) | 683.04  (521.88-860.09) | -0.43 (-0.54 to -0.31) | <0.001 |
| Namibia | 5026  (3483-6555) | 568.83  (397.92-749.83) | 12421  (8585-16204) | 643.31  (452.21-831.78) | 0.42 (0.34 to 0.5) | <0.001 |
| Nauru | 80  (60-97) | 1084.31  (857.84-1270.5) | 23  (17-33) | 310.75  (230.89-432.15) | -3.92 (-4.17 to -3.66) | <0.001 |
| Nepal | 135014  (93620-172740) | 938.26  (666.69-1192.67) | 281548  (212315-359487) | 959.42  (738.59-1211.78) | 0.07 (-0.03 to 0.16) | 0.162 |
| Netherlands | 166168  (124638-204002) | 939.74  (697.39-1150.9) | 202758  (162053-250988) | 863.42  (662.18-1097.65) | -0.26 (-0.32 to -0.19) | <0.001 |
| New Zealand | 34119  (25445-42286) | 926.02  (683.86-1143.19) | 73140  (63129-84142) | 1190.23  (1006.58-1388.55) | 0.73 (0.65 to 0.81) | <0.001 |
| Nicaragua | 28276  (23567-33442) | 1124.24  (971.39-1286.91) | 70677  (56582-87320) | 1108.25  (908.1-1330.78) | -0.04 (-0.06 to -0.02) | <0.001 |
| Niger | 81976  (62075-95771) | 1425.57  (1119.73-1617.46) | 95784  (67958-133006) | 650.99  (473.19-881.47) | -2.48 (-2.57 to -2.39) | <0.001 |
| Nigeria | 1038884  (821843-1203856) | 1437.53  (1158.53-1617.42) | 718748  (515836-985688) | 484  (354.4-655.65) | -3.33 (-3.76 to -2.9) | <0.001 |
| Niue | 21  (16-24) | 1014.35  (798.72-1204.95) | 6  (5-9) | 317  (238.9-429.73) | -3.64 (-3.8 to -3.49) | <0.001 |
| North Macedonia | 17816  (13232-21797) | 876.58  (656.07-1068.3) | 26498  (21026-32028) | 901.63  (693.42-1111.26) | 0.09 (0.08 to 0.1) | <0.001 |
| Northern Mariana Islands | 506  (383-617) | 1169.8  (961.92-1344.72) | 202  (143-279) | 367.48  (274.16-494.57) | -3.79 (-4.02 to -3.57) | <0.001 |
| Norway | 65290  (53795-73447) | 1333.41  (1069.62-1513.66) | 83211  (72700-94076) | 1274.2  (1092.81-1452.46) | -0.16 (-0.23 to -0.09) | <0.001 |
| Oman | 15147  (10748-19025) | 1070.66  (852.6-1260.01) | 58103  (44068-75685) | 1152.6  (961.83-1379.04) | 0.24 (0.22 to 0.27) | <0.001 |
| Pakistan | 1272050  (1034722-1463414) | 1457.82  (1206.17-1627.85) | 3056770  (2619588-3458237) | 1423.01  (1257.58-1589.96) | -0.08 (-0.1 to -0.07) | <0.001 |
| Palau | 138  (105-167) | 1039.56  (830.03-1221.8) | 75  (54-103) | 323.8  (245.42-443.55) | -3.7 (-3.82 to -3.59) | <0.001 |
| Palestine | 10000  (7332-12558) | 853.51  (630.54-1045.55) | 38761  (29487-50933) | 967.11  (759.68-1195.38) | 0.4 (0.36 to 0.45) | <0.001 |
| Panama | 24334  (18775-29184) | 1196.51  (956.96-1390.84) | 54607  (46341-63054) | 1242.73  (1055.32-1432.24) | 0.12 (0.11 to 0.13) | <0.001 |
| Papua New Guinea | 24495  (18068-30857) | 902.24  (683.84-1099.13) | 17316  (12274-23934) | 247.45  (184.08-342.87) | -4.05 (-4.28 to -3.82) | <0.001 |
| Paraguay | 32897  (24721-39605) | 1101.83  (861.19-1291.6) | 75927  (60330-95420) | 1081.69  (879.15-1308.09) | -0.06 (-0.1 to -0.01) | 0.02 |
| Peru | 192761  (163403-225012) | 1145.76  (998.73-1293.53) | 432932  (361718-515426) | 1156.05  (975.11-1354.83) | 0.04 (0.02 to 0.05) | <0.001 |
| Philippines | 470411  (352132-570174) | 1044.17  (822.62-1236.76) | 418410  (343422-508733) | 427.33  (349.73-520.01) | -2.82 (-3.14 to -2.5) | <0.001 |
| Poland | 456657  (365890-531169) | 1088.97  (875.99-1271.87) | 566714  (490754-653701) | 1115.26  (940.64-1328.54) | 0.08 (0.06 to 0.1) | <0.001 |
| Portugal | 100052  (74431-124210) | 857.39  (626.84-1067.24) | 127192  (100895-156849) | 812.27  (622.51-1022.21) | -0.17 (-0.2 to -0.14) | <0.001 |
| Puer to Rico | 48856  (39722-55676) | 1344.54  (1096.81-1526.42) | 52562  (45774-59456) | 1278.82  (1092.38-1465.54) | -0.16 (-0.19 to -0.14) | <0.001 |
| Qatar | 5295  (3755-6621) | 1201.84  (965.5-1393.28) | 47937  (37154-59213) | 1255.27  (1083.59-1450.39) | 0.14 (0.13 to 0.16) | <0.001 |
| Republic of Korea | 283312  (204663-355093) | 711.01  (530.08-884.75) | 604130  (482014-733542) | 775.99  (609.98-957.48) | 0.29 (0.26 to 0.31) | <0.001 |
| Republic of Moldova | 42977  (33437-51642) | 942.41  (729.28-1132.68) | 44764  (35482-54268) | 894.89  (696.24-1102.99) | -0.16 (-0.22 to -0.09) | <0.001 |
| Romania | 230516  (174119-280436) | 878.52  (665.32-1073.03) | 246702  (203277-293644) | 932.55  (735.17-1140.69) | 0.19 (0.16 to 0.23) | <0.001 |
| Russian Federation | 1880312  (1514813-2186584) | 1098.1  (882.52-1278.08) | 1990711  (1692298-2328403) | 1049.06  (869.8-1255.05) | -0.14 (-0.16 to -0.12) | <0.001 |
| Rwanda | 68784  (50871-83546) | 1334.53  (1047.21-1540.25) | 89798  (64603-120425) | 885.71  (670.26-1127.29) | -1.3 (-1.46 to -1.14) | <0.001 |
| Saint Kitts and Nevis | 477  (376-562) | 1293.97  (1046.08-1490.48) | 881  (747-1011) | 1250.9  (1058.78-1444.52) | -0.12 (-0.14 to -0.09) | <0.001 |
| Saint Lucia | 1403  (1075-1690) | 1262.8  (997.31-1467.49) | 2565  (2142-2958) | 1193.21  (998.09-1394.25) | -0.18 (-0.23 to -0.13) | <0.001 |
| Saint Vincent and the Grenadines | 1083  (823-1302) | 1229.49  (976.5-1427.27) | 1502  (1255-1744) | 1180.56  (982.99-1387.84) | -0.12 (-0.15 to -0.1) | <0.001 |
| Samoa | 1028  (787-1259) | 947.2  (732.99-1138.22) | 437  (317-603) | 272.06  (199.91-369.95) | -3.93 (-4.06 to -3.79) | <0.001 |
| San Marino | 284  (221-342) | 999.82  (780.1-1214.31) | 414  (333-505) | 881.51  (685.45-1095.73) | -0.41 (-0.46 to -0.36) | <0.001 |
| Sao tome and Principe | 1387  (1126-1594) | 1476.2  (1216.65-1643.45) | 2563  (2075-3056) | 1334.37  (1114.14-1539.77) | -0.33 (-0.36 to -0.31) | <0.001 |
| Saudi Arabia | 74010  (60692-88173) | 732.49  (595.87-880) | 339963  (233000-459412) | 830.6  (635.05-1061.23) | 0.41 (0.37 to 0.45) | <0.001 |
| Senegal | 86585  (70193-98189) | 1481.89  (1217.16-1646.17) | 174404  (140198-210379) | 1331.06  (1121.39-1529.51) | -0.35 (-0.4 to -0.3) | <0.001 |
| Serbia | 99522  (76540-120462) | 897.29  (679.93-1092.99) | 110914  (90522-132346) | 905.69  (704.95-1108.22) | 0.04 (0 to 0.08) | 0.052 |
| Seychelles | 675  (511-820) | 1073.78  (836.56-1272.97) | 1017  (789-1247) | 814.53  (647.04-996.26) | -0.88 (-0.97 to -0.79) | <0.001 |
| Sierra Leone | 39192  (28849-47613) | 1165.55  (867.36-1399.93) | 124421  (110782-137848) | 1578.93  (1439.14-1728.99) | 1 (0.95 to 1.05) | <0.001 |
| Singapore | 24234  (18044-30244) | 802.15  (609.62-981.47) | 69150  (54549-84735) | 844.12  (668.98-1034.3) | 0.16 (0.11 to 0.2) | <0.001 |
| Slovakia | 52663  (40124-63607) | 922.89  (705.48-1110.99) | 71447  (58766-84274) | 957.4  (761.25-1173.57) | 0.12 (0.11 to 0.14) | <0.001 |
| Slovenia | 27491  (24534-30378) | 1207.1  (1066.16-1335.29) | 32572  (29056-36643) | 1174.44  (1007.56-1387.42) | -0.09 (-0.11 to -0.07) | <0.001 |
| Solomon Islands | 1672  (1229-2077) | 850.58  (637.99-1049.46) | 1033  (736-1435) | 226.74  (168.75-312.03) | -4.13 (-4.33 to -3.92) | <0.001 |
| Somalia | 62741  (45341-76759) | 1188.98  (904.57-1412.24) | 70472  (49308-97382) | 587.1  (439.47-783.36) | -2.24 (-2.4 to -2.07) | <0.001 |
| South Africa | 217806  (159646-275991) | 805.55  (593.11-1010.38) | 330616  (259523-426360) | 600.5  (481.27-763.54) | -0.93 (-1.03 to -0.83) | <0.001 |
| South Sudan | 66062  (51566-77917) | 1448.18  (1187.39-1630.35) | 55145  (40099-72471) | 869.86  (660.08-1098.23) | -1.66 (-1.73 to -1.59) | <0.001 |
| Spain | 399089  (296664-491298) | 895.6  (661.14-1103.65) | 160821  (115337-222403) | 228.16  (166.81-312.89) | -4.38 (-4.67 to -4.09) | <0.001 |
| Sri Lanka | 213138  (167378-246647) | 1347.56  (1095.36-1524.81) | 196474  (153954-242980) | 752.64  (584.21-937.65) | -1.82 (-2 to -1.63) | <0.001 |
| Sudan | 102039  (72880-128948) | 813.31  (596.39-1011.67) | 272441  (198775-343292) | 881.01  (665.28-1077.56) | 0.26 (0.25 to 0.27) | <0.001 |
| Suriname | 4380  (3433-5184) | 1280.3  (1024.73-1475.09) | 7429  (6166-8661) | 1205.24  (997.39-1410.66) | -0.19 (-0.24 to -0.14) | <0.001 |
| Sweden | 112115  (89108-131215) | 1052.82  (828.84-1253.06) | 131195  (110216-156162) | 961.45  (768.73-1184.47) | -0.19 (-0.25 to -0.14) | <0.001 |
| Switzerland | 83142  (63912-100655) | 977.77  (744.55-1190.68) | 110339  (88552-133613) | 891.29  (688.25-1118.42) | -0.3 (-0.33 to -0.26) | <0.001 |
| Syrian Arab Republic | 67197  (49523-83699) | 894.39  (668.98-1093.15) | 136563  (103824-166057) | 958.19  (760.45-1180.56) | 0.22 (0.18 to 0.27) | <0.001 |
| Taiwan (Province of China) | 163327  (125664-199480) | 869.27  (681.31-1047.15) | 312753  (243545-370291) | 884.83  (690.09-1059.09) | 0.05 (0.02 to 0.08) | 0.001 |
| Tajikistan | 32624  (24662-39739) | 949.93  (731.47-1144.17) | 69339  (50841-91521) | 840.21  (645.92-1053.33) | -0.4 (-0.45 to -0.35) | <0.001 |
| Thailand | 465315  (367969-551916) | 946.59  (766.22-1110.52) | 966442  (833869-1104786) | 1057.59  (901.49-1268.18) | 0.37 (0.32 to 0.42) | <0.001 |
| Timor-Leste | 4510  (3184-5783) | 883.5  (658.19-1087.84) | 6700  (5091-8470) | 683.4  (523.31-846.6) | -0.85 (-1.01 to -0.68) | <0.001 |
| Togo | 40263  (31375-46473) | 1454.83  (1176.14-1622.37) | 87310  (68026-107309) | 1260.08  (1024.25-1481.33) | -0.46 (-0.48 to -0.44) | <0.001 |
| Tokelau | 12  (9-15) | 939.7  (726.38-1125.78) | 4  (3-5) | 284.6  (212.16-385.53) | -3.77 (-3.92 to -3.63) | <0.001 |
| Tonga | 623  (478-756) | 942.19  (740.24-1138.11) | 232  (171-313) | 272.69  (201.25-366.02) | -3.91 (-4.16 to -3.65) | <0.001 |
| Trinidad and Tobago | 13891  (11948-15615) | 1267.89  (1120.09-1404.24) | 20759  (18045-23737) | 1275.07  (1092.82-1462.29) | 0.02 (0 to 0.03) | 0.045 |
| Tunisia | 59398  (44128-73301) | 936.74  (709.22-1136.48) | 141663  (116163-171012) | 1056.72  (868.82-1284.3) | 0.39 (0.35 to 0.43) | <0.001 |
| Turkey | 305662  (218088-392975) | 696.63  (504.63-884.46) | 1110677  (953336-1296103) | 1182.8  (1011.76-1404.29) | 1.7 (1.63 to 1.78) | <0.001 |
| Turkmenistan | 25274  (19021-30824) | 996.11  (777.79-1180.59) | 49779  (39927-61514) | 1006.31  (828.73-1222.19) | 0.02 (-0.01 to 0.06) | 0.137 |
| Tuvalu | 68  (51-84) | 870.43  (657.57-1058.75) | 27  (20-37) | 250.37  (185.03-339.52) | -3.98 (-4.13 to -3.83) | <0.001 |
| Uganda | 156793  (114288-192479) | 1322.08  (1035.89-1541.64) | 235989  (171929-326148) | 882.95  (666.36-1133.16) | -1.27 (-1.41 to -1.13) | <0.001 |
| Ukraine | 662840  (543405-762712) | 1072.74  (863.41-1252.22) | 597141  (496354-699687) | 991.89  (803.97-1192.24) | -0.25 (-0.27 to -0.23) | <0.001 |
| United Arab Emirates | 21821  (15544-27282) | 1231.46  (998.36-1430.68) | 159298  (119899-193697) | 1202.43  (1023.71-1412.48) | -0.08 (-0.09 to -0.06) | <0.001 |
| United Kingdom | 433953  (313198-557556) | 611.83  (435.21-784.87) | 549059  (441586-687981) | 578.33  (466.7-733.35) | -0.18 (-0.25 to -0.11) | <0.001 |
| United Republic of Tanzania | 255035  (193021-308644) | 1366.05  (1079.92-1564.42) | 377499  (277649-494271) | 917.6  (703.67-1146.07) | -1.25 (-1.36 to -1.15) | <0.001 |
| United States of America | 2754029  (2207724-3254430) | 958.82  (762.72-1126.99) | 3888867  (3358269-4445248) | 894.23  (759.99-1031.8) | -0.11 (-0.16 to -0.07) | <0.001 |
| United States Virgin Islands | 1382  (1123-1568) | 1340.38  (1084.17-1526.16) | 1360  (1187-1532) | 1294.13  (1107.32-1470.37) | -0.24 (-0.35 to -0.14) | <0.001 |
| Uruguay | 42090  (34075-48135) | 1288.53  (1015.45-1486.66) | 51331  (45124-58123) | 1310.74  (1130.56-1504.11) | 0.06 (0.04 to 0.09) | <0.001 |
| Uzbekistan | 132457  (97957-163038) | 931.36  (699.37-1119.59) | 311361  (233532-389183) | 931.78  (728.42-1142.55) | 0 (-0.03 to 0.04) | 0.793 |
| Vanuatu | 838  (615-1050) | 889.26  (673.52-1081.55) | 510  (365-715) | 234.16  (175.25-321.75) | -4.16 (-4.38 to -3.93) | <0.001 |
| Venezuela (Bolivarian Republic of) | 190454  (159767-217923) | 1234.89  (1075.36-1366.04) | 339319  (281366-395690) | 1176.94  (971.69-1380.07) | -0.15 (-0.17 to -0.13) | <0.001 |
| Viet Nam | 267066  (191321-345024) | 559.01  (395.37-723.55) | 784468  (587669-988093) | 714.24  (550.68-891.89) | 0.79 (0.7 to 0.89) | <0.001 |
| Yemen | 70912  (60022-82824) | 948.73  (825.92-1081.04) | 232745  (174803-306667) | 972.42  (764.21-1195.61) | 0.07 (0.02 to 0.12) | 0.003 |
| Zambia | 78845  (60081-95510) | 1388.93  (1091.46-1594.87) | 127358  (91104-176223) | 953.58  (737.39-1203.69) | -1.18 (-1.3 to -1.05) | <0.001 |
| Zimbabwe | 29862  (20140-38846) | 511.42  (353.27-668.8) | 49907  (33509-64576) | 479.63  (331.77-630.93) | -0.2 (-0.26 to -0.14) | <0.001 |

AAPC: average annual percent change; ASR: age-standardized rate.
