# Supplementary material for: Global, regional, and national burden of periodontal diseases from 1990 to 2021 and predictions to 2040: an analysis of the global burden of disease study 2021
Source: Front Oral Health. 2025 Jul 24;6:1627746. doi: 10.3389/froh.2025.1627746 (PMC12332980; doi:10.3389/froh.2025.1627746)
Supplement: Supplementary file 3 [file Table2.docx]

**Supplementary Table S2**  The case number and ASR of prevalence of periodontal diseases in 1990 and 2021 across 204 countries and territories, with AAPC from 1990 to 2021.

| **Location** | **1990** | | **2021** | | **AAPC(95%CI)**  **1990–2021** | ***P*** |
| --- | --- | --- | --- | --- | --- | --- |
|  | **Number (95%UI)** | **ASR (95%UI)** | **Number (95%UI)** | **ASR (95%UI)** |  |  |
| Afghanistan | 551574  (386044-730300) | 7677.61  (5449.72-10070.44) | 1432632  (997497-2004968) | 8410.8  (6198.93-11222.16) | 0.31 (0.25 to 0.38) | <0.001 |
| Albania | 192002  (134900-252476) | 7564.32  (5310.2-9851.95) | 320795  (243474-411139) | 8876.36  (6565.41-11623.42) | 0.53 (0.49 to 0.56) | <0.001 |
| Algeria | 1655691  (1221072-2108405) | 10647.37  (8066.29-13648.56) | 5119712  (3817960-6708009) | 11708.1  (8938.51-14949.73) | 0.3 (0.28 to 0.32) | <0.001 |
| American Samoa | 4187  (3130-5323) | 13171.06  (10147.17-16230.49) | 6626  (5062-8167) | 12617.46  (9642.82-15478.66) | -0.14 (-0.15 to -0.13) | <0.001 |
| Andorra | 7389  (5379-9418) | 11817.89  (8653.39-15021.85) | 13470  (10077-17233) | 10003.01  (7381.42-12974.47) | -0.53 (-0.57 to -0.49) | <0.001 |
| Angola | 1171034  (895587-1431006) | 18164.8  (14386.04-21720.84) | 2222932  (1604818-3014744) | 11943.1  (8909.73-15438.92) | -1.32 (-1.46 to -1.18) | <0.001 |
| Antigua and Barbuda | 9023  (7084-10931) | 16880.24  (13406.23-20270.27) | 16880  (13342-20203) | 15449.28  (12132.43-18610.82) | -0.28 (-0.33 to -0.23) | <0.001 |
| Argentina | 3761568  (2777664-4764585) | 11764.76  (8667.83-14932.72) | 6260216  (4673548-7951424) | 12191.42  (9026.77-15649.23) | 0.16 (0.08 to 0.24) | <0.001 |
| Armenia | 301661  (222570-387116) | 9831.19  (7291.2-12514.24) | 356734  (268781-449693) | 9281.47  (6965.7-11902.93) | -0.17 (-0.22 to -0.12) | <0.001 |
| Australia | 1495433  (1073509-1932338) | 8050.24  (5754.29-10448.4) | 3136690  (2327567-4056205) | 9328.74  (6802.3-12536.83) | 0.45 (0.18 to 0.72) | 0.001 |
| Austria | 955244  (695521-1225658) | 9963.66  (7134.52-12853.02) | 1199000  (902425-1536564) | 9084.32  (6627.86-11918.85) | -0.31 (-0.44 to -0.19) | <0.001 |
| Azerbaijan | 605583  (449677-772143) | 10519.19  (7917.59-13203.63) | 1218878  (911908-1573231) | 9974.58  (7570.73-12752.6) | -0.17 (-0.23 to -0.11) | <0.001 |
| Bahamas | 37633  (29161-45939) | 17546.38  (13984.34-20931.61) | 70352  (56030-85246) | 15746.69  (12498.5-19109.7) | -0.35 (-0.4 to -0.29) | <0.001 |
| Bahrain | 47978  (33106-62286) | 12538.54  (9455.11-15697.32) | 239786  (178969-311863) | 13668.35  (10726.66-17108.9) | 0.28 (0.27 to 0.29) | <0.001 |
| Bangladesh | 12376464  (9341962-15110338) | 17947.96  (13787.06-21654.01) | 30518641  (24018938-36675110) | 19172.58  (15328.19-22820.82) | 0.21 (0.2 to 0.22) | <0.001 |
| Barbados | 41957  (32701-50799) | 16542.45  (13019.24-19957.96) | 58898  (47612-69999) | 14702.68  (11591.4-17941.27) | -0.39 (-0.5 to -0.28) | <0.001 |
| Belarus | 1341820  (1020481-1667557) | 10966.04  (8235.94-13651.39) | 1463365  (1122187-1810727) | 10783.01  (8164.89-13733.7) | -0.05 (-0.08 to -0.03) | <0.001 |
| Belgium | 1917610  (1531331-2321318) | 15715.07  (12289.22-19231.3) | 2309494  (1880743-2709725) | 15019.93  (12001.74-18132.51) | -0.15 (-0.23 to -0.07) | <0.001 |
| Belize | 16696  (12398-20962) | 14505.2  (11033.75-17933.4) | 53009  (40293-66922) | 13676.79  (10544.81-17046.08) | -0.19 (-0.27 to -0.11) | <0.001 |
| Benin | 671142  (533027-793985) | 22564.08  (18401.83-26171.16) | 1556703  (1200599-1938718) | 17999.85  (14199.75-21577.27) | -0.72 (-0.81 to -0.62) | <0.001 |
| Bermuda | 12367  (9748-14877) | 17973.25  (14270.4-21483.59) | 16835  (14033-19408) | 18586.86  (14920.39-22083.71) | 0.11 (0.09 to 0.13) | <0.001 |
| Bhutan | 74336  (54842-91132) | 18399.5  (14067.01-21972.69) | 152538  (122026-180846) | 20136.96  (16406.82-23558.37) | 0.29 (0.28 to 0.3) | <0.001 |
| Bolivia (Plurinational State of) | 491132  (358587-629048) | 11209.73  (8409.97-14239.13) | 1215095  (902351-1593127) | 10917.38  (8189.2-14085.98) | -0.09 (-0.16 to -0.02) | 0.015 |
| Bosnia and Herzegovina | 298780  (203113-402096) | 6355.26  (4418.65-8498.26) | 438061  (324672-556907) | 8890.37  (6538.09-11534.08) | 1.1 (1.01 to 1.19) | <0.001 |
| Botswana | 41487  (28906-56226) | 5699.14  (4012.23-7649.68) | 92048  (62616-130284) | 4517.68  (3200.47-6210.18) | -0.69 (-0.82 to -0.57) | <0.001 |
| Brazil | 12601676  (9301883-15979406) | 10799.58  (8113.44-13571.15) | 31757123  (25420508-38026130) | 12390.21  (9913.63-14796.27) | 0.48 (0.35 to 0.6) | <0.001 |
| Brunei Darussalam | 17576  (12717-22449) | 10803.47  (8282.1-13387.62) | 42461  (31484-56484) | 9077.38  (6920.76-11561.07) | -0.57 (-0.64 to -0.5) | <0.001 |
| Bulgaria | 794115  (552852-1056783) | 6976.16  (4886.24-9290.89) | 791513  (575356-1025194) | 7345.3  (5315.39-9664.45) | 0.17 (0.09 to 0.24) | <0.001 |
| Burkina Faso | 1313997  (1053291-1575768) | 22190.85  (18201.4-26346.31) | 3394559  (2708332-4050578) | 22601.68  (18482.57-26418.45) | 0.06 (0.04 to 0.07) | <0.001 |
| Burundi | 630490  (473453-773789) | 18692.38  (14483.32-22655.32) | 660631  (459206-934510) | 8728.04  (6257.55-11647.66) | -2.39 (-2.49 to -2.28) | <0.001 |
| Cabo Verde | 55065  (45467-64074) | 23080.9  (19562.66-26580.41) | 140191  (119246-160640) | 24515.84  (21076.04-27960.25) | 0.2 (0.19 to 0.2) | <0.001 |
| Cambodia | 480131  (330814-635254) | 8099.28  (5719.82-10637.19) | 1518824  (1081627-1995926) | 10014.83  (7241.54-12936.91) | 0.69 (0.65 to 0.72) | <0.001 |
| Cameroon | 1615788  (1331655-1894649) | 23538.17  (19742.68-26973.51) | 5235972  (4255674-6211402) | 23210.96  (19406.11-26730.23) | -0.04 (-0.05 to -0.04) | <0.001 |
| Canada | 4486564  (3592118-5422738) | 14355.06  (11446.88-17354.94) | 7387513  (6106135-8803879) | 14036.47  (11346.76-17276.54) | -0.07 (-0.09 to -0.05) | <0.001 |
| Central African Republic | 279224  (211167-349661) | 16071.44  (12356.34-19666.48) | 281304  (193535-402754) | 8224.98  (5947.44-10903.17) | -2.13 (-2.2 to -2.06) | <0.001 |
| Chad | 826103  (651765-992940) | 21679.84  (17467.91-25497.59) | 1699563  (1297315-2174717) | 17296.61  (13551.36-20994.34) | -0.76 (-0.86 to -0.66) | <0.001 |
| Chile | 1475469  (1088926-1842398) | 12569.3  (9418.37-15686.92) | 3253544  (2579964-3955133) | 14236.18  (11137.81-17415.54) | 0.33 (0.15 to 0.51) | <0.001 |
| China | 110887970  (83432920-140479922) | 11077.39  (8383.08-13872.18) | 220238287  (183074953-258996015) | 10799.46  (8883.18-12782.71) | -0.08 (-0.31 to 0.15) | 0.484 |
| Colombia | 3464079  (2564034-4325906) | 14278.18  (10880.37-17497.97) | 7959558  (6240568-9705113) | 14600.31  (11448.73-17858.22) | 0.07 (0.05 to 0.09) | <0.001 |
| Comoros | 57983  (45728-69650) | 20243.57  (16486.55-23760.96) | 67332  (48674-91747) | 10846.09  (8057.89-14324.33) | -1.96 (-1.98 to -1.94) | <0.001 |
| Congo | 282745  (216350-341334) | 18487.78  (14596.15-21874.87) | 476336  (336307-653091) | 11578.89  (8529.41-15030.44) | -1.52 (-1.63 to -1.42) | <0.001 |
| Cook Islands | 1824  (1372-2268) | 12371.32  (9499.9-15226.2) | 785  (552-1089) | 3378.73  (2396.25-4550.56) | -4.15 (-4.36 to -3.94) | <0.001 |
| Costa Rica | 324215  (244099-407902) | 14318.94  (11061.72-17586.13) | 791415  (628710-962589) | 14589.27  (11534.02-17770.49) | 0.06 (0.03 to 0.08) | <0.001 |
| Coted'Ivoire | 1842193  (1496561-2194773) | 23247.81  (19744.66-26658.46) | 3726630  (2890925-4599867) | 18565.43  (14746.06-22162.12) | -0.74 (-0.78 to -0.69) | <0.001 |
| Croatia | 654362  (574927-748651) | 10900.01  (9621.83-12474) | 788908  (641462-953029) | 12734.7  (10074.84-16205.5) | 0.5 (0.44 to 0.57) | <0.001 |
| Cuba | 1670877  (1292647-2040953) | 15533.96  (12033.58-18962.02) | 2149316  (1706330-2630178) | 13865.4  (10738.63-17372.56) | -0.37 (-0.41 to -0.32) | <0.001 |
| Cyprus | 80958  (57283-103359) | 9941.44  (7044.71-12677.96) | 162787  (118609-215534) | 8842.54  (6377.85-11733.67) | -0.39 (-0.49 to -0.3) | <0.001 |
| Czechia | 1218171  (904680-1557941) | 9780.74  (7220.42-12524.93) | 1637567  (1267226-2043346) | 10326.71  (7765.94-13370.44) | 0.19 (0.14 to 0.25) | <0.001 |
| Democratic People's Republic of Korea | 1377429  (962244-1833168) | 7367.46  (5310.68-9650.88) | 2234849  (1539660-3078492) | 6550.08  (4584.23-8915.05) | -0.38 (-0.41 to -0.35) | <0.001 |
| Democratic Republic of the Congo | 3957284  (2950338-4851352) | 16986.29  (13056.92-20456.2) | 4687391  (3220154-6591349) | 8542.82  (6050.81-11373) | -2.19 (-2.33 to -2.05) | <0.001 |
| Denmark | 1368486  (1185603-1547031) | 21994.07  (18856.95-24995.63) | 1564150  (1364941-1768363) | 20822.45  (17818.3-23941.18) | -0.18 (-0.22 to -0.14) | <0.001 |
| Djibouti | 55937  (43347-68041) | 20973.39  (17051.03-24596.74) | 119959  (82677-167721) | 11742.85  (8590.84-15582.22) | -1.81 (-1.85 to -1.78) | <0.001 |
| Dominica | 8826  (6770-10847) | 15190.07  (11612.63-18551.22) | 11172  (8823-13713) | 14206.47  (11095.24-17609.25) | -0.22 (-0.26 to -0.18) | <0.001 |
| Dominican Republic | 731424  (542223-914559) | 14441.67  (10993.9-17654.53) | 1591604  (1245522-1960891) | 14430.58  (11339.27-17629.97) | 0 (-0.05 to 0.05) | 0.937 |
| Ecuador | 874438  (717112-1039341) | 12241.41  (10213.98-14458.29) | 2128515  (1617152-2681557) | 12041.43  (9171.2-15104.12) | -0.03 (-0.09 to 0.03) | 0.263 |
| Egypt | 3432823  (2409591-4573221) | 9133.29  (6577.07-11860.56) | 9941894  (7255243-13260647) | 11311.42  (8553.28-14691.38) | 0.69 (0.63 to 0.76) | <0.001 |
| El Salvador | 455220  (336923-579353) | 12737  (9570.25-16138.37) | 823631  (633110-1027698) | 13330.13  (10291.34-16654.74) | 0.15 (0.12 to 0.18) | <0.001 |
| Equatorial Guinea | 45238  (34414-55143) | 16986.18  (13129.98-20348.5) | 130974  (95952-174534) | 14099  (10876.88-17705.61) | -0.57 (-0.81 to -0.34) | <0.001 |
| Eritrea | 377775  (282239-466172) | 18638.68  (14432.03-22454.79) | 452056  (315086-649699) | 10190.06  (7396.46-13466.64) | -1.97 (-2.15 to -1.79) | <0.001 |
| Estonia | 209999  (159318-260040) | 11054.94  (8262.72-13786.64) | 219424  (174809-267023) | 11482.9  (8899.36-14616.94) | 0.13 (0.11 to 0.16) | <0.001 |
| Eswatini | 22454  (15457-30160) | 5619.41  (3928.55-7458.95) | 50785  (35377-69524) | 6494.78  (4562.52-8771.63) | 0.46 (0.42 to 0.5) | <0.001 |
| Ethiopia | 5137354  (3880675-6262666) | 17445.67  (13613.95-20905.22) | 12587645  (9588925-15455462) | 17836.13  (14116.41-21413.57) | 0.08 (0.07 to 0.09) | <0.001 |
| Fiji | 56504  (40949-72635) | 10908.27  (8179.78-13693.12) | 22929  (16119-31775) | 2603.69  (1879.22-3582.89) | -4.48 (-4.73 to -4.22) | <0.001 |
| Finland | 804829  (605825-992345) | 13112.89  (9851.18-16273.16) | 1043320  (847688-1253182) | 13563.08  (10645.72-17135.6) | 0.14 (0.03 to 0.25) | 0.011 |
| France | 4589309  (3630782-5649948) | 6602.57  (5193.77-8204.87) | 6879081  (5123976-8954566) | 7138.11  (5076.86-9478.09) | 0.23 (-0.01 to 0.47) | 0.056 |
| Gabon | 144210  (117662-169517) | 20600.49  (16962.14-23965.65) | 190323  (137804-248649) | 13472.71  (10063.07-16995.23) | -1.38 (-1.44 to -1.32) | <0.001 |
| Gambia | 160950  (133869-187193) | 25315.57  (21838.11-28579.66) | 431120  (359149-499940) | 25113.82  (21593.04-28327.1) | -0.02 (-0.06 to 0.01) | 0.129 |
| Georgia | 653199  (494046-813964) | 10718.18  (8034.73-13470.3) | 463109  (356755-579377) | 9621.48  (7333.36-12431.81) | -0.35 (-0.4 to -0.3) | <0.001 |
| Germany | 16658684  (14917137-18519328) | 16332.35  (14544.94-18240.76) | 19878957  (16669883-23129257) | 16458.22  (13310.52-19650.66) | -0.01 (-0.18 to 0.16) | 0.876 |
| Ghana | 2344356  (1922575-2772210) | 23025.6  (19403.56-26506.67) | 6550038  (5391887-7684182) | 23882.45  (20287.6-27427.99) | 0.12 (0.11 to 0.13) | <0.001 |
| Greece | 1144917  (815004-1478739) | 8854.29  (6281.82-11531.17) | 1391412  (1051524-1816223) | 8829.49  (6479.64-11682.05) | 0.05 (-0.07 to 0.16) | 0.435 |
| Greenland | 5576  (3979-7236) | 11310.08  (8487.37-14047.82) | 7921  (6032-10083) | 11287.96  (8598.05-14461.35) | -0.01 (-0.04 to 0.03) | 0.654 |
| Grenada | 9633  (7335-11928) | 14867.06  (11233.11-18261.7) | 17083  (13440-20886) | 14619.59  (11427.47-18099.59) | -0.07 (-0.16 to 0.03) | 0.173 |
| Guam | 15394  (11656-19187) | 14012.11  (11096.48-17136.52) | 7222  (5115-9656) | 3679.38  (2618.52-4965.1) | -4.22 (-4.38 to -4.07) | <0.001 |
| Guatemala | 633360  (464187-807211) | 12989.86  (9797.37-16362.95) | 1740576  (1318298-2198936) | 13084.09  (10088.69-16352.2) | 0.02 (0 to 0.04) | 0.059 |
| Guinea | 952056  (771155-1111556) | 22731.94  (18649.05-26239.37) | 2029047  (1608793-2423992) | 22514.99  (18498.26-26355.08) | -0.03 (-0.04 to -0.01) | <0.001 |
| Guinea-Bissau | 140937  (111182-168095) | 22264.08  (18278.58-25896.98) | 232221  (175467-296042) | 17147.35  (13162.09-20918.19) | -0.84 (-0.99 to -0.69) | <0.001 |
| Guyana | 76239  (55885-96933) | 13962.98  (10639.95-17305.14) | 102094  (77967-128120) | 13779.82  (10605.04-17239.57) | -0.04 (-0.13 to 0.04) | 0.351 |
| Haiti | 567515  (413051-720531) | 12972.37  (9678.77-16159.42) | 1157290  (821741-1609318) | 10915.51  (8028.96-14476.38) | -0.54 (-0.62 to -0.47) | <0.001 |
| Honduras | 348305  (252713-445854) | 12601.77  (9338.02-15983.8) | 1034410  (772535-1363782) | 12524.9  (9471.27-16068.13) | -0.03 (-0.05 to 0) | 0.028 |
| Hungary | 558361  (379270-776921) | 4199.01  (2849.36-5727.92) | 702307  (506777-948709) | 4660.88  (3333.71-6298.42) | 0.36 (0.27 to 0.46) | <0.001 |
| Iceland | 25661  (20775-30795) | 9822.47  (7885.16-11745.41) | 42367  (31887-53715) | 9262.14  (6779.7-12008.03) | -0.19 (-0.22 to -0.16) | <0.001 |
| India | 101651450  (76544925-125150617) | 15701.48  (12074.91-19051.39) | 238128487  (198522214-274680070) | 16920.14  (14249.78-19402.41) | 0.23 (0.18 to 0.28) | <0.001 |
| Indonesia | 17894239  (13214233-22393604) | 13220.99  (10059.75-16313.77) | 41014679  (30942526-50701865) | 13659.79  (10473.05-16815.61) | 0.11 (0.09 to 0.13) | <0.001 |
| Iran (Islamic Republic of) | 4257624  (3135245-5366187) | 12313.17  (9298.35-15281.19) | 11963222  (9411959-14461389) | 12453.29  (9984.02-14855.87) | 0.03 (-0.01 to 0.07) | 0.166 |
| Iraq | 1107948  (799608-1424128) | 10522.21  (7838.35-13507.51) | 4058386  (2976364-5421998) | 11844.33  (9055.15-15294.16) | 0.39 (0.34 to 0.45) | <0.001 |
| Ireland | 181333  (125711-244115) | 5003.48  (3441.39-6767.64) | 366285  (263944-491134) | 5572.73  (3995.82-7632.5) | 0.35 (0.27 to 0.43) | <0.001 |
| Israel | 452767  (327242-581341) | 9912.91  (7120.83-12701.56) | 900798  (673678-1165769) | 8690.01  (6375.94-11398.3) | -0.41 (-0.51 to -0.3) | <0.001 |
| Italy | 7542947  (5626098-9465326) | 10186.62  (7500.41-12978.76) | 9146807  (7017499-11464359) | 9401.12  (7017.55-12061.04) | -0.25 (-0.31 to -0.2) | <0.001 |
| Jamaica | 271503  (206873-339426) | 15037.77  (11565.23-18436.85) | 427610  (328887-534359) | 13836.51  (10621.12-17297.44) | -0.27 (-0.38 to -0.17) | <0.001 |
| Japan | 14859067  (10936839-18725440) | 9038.06  (6600.65-11426.9) | 20072088  (15783257-24356032) | 9211.73  (6990.71-11658.37) | 0.02 (-0.12 to 0.17) | 0.732 |
| Jordan | 203382  (146110-265243) | 10004.61  (7459.58-12821.76) | 1226574  (890255-1628196) | 11231.57  (8490.96-14488.92) | 0.38 (0.31 to 0.45) | <0.001 |
| Kazakhstan | 1514563  (1120400-1898024) | 10564  (7903.49-13188.94) | 2066326  (1538304-2674207) | 10259.21  (7730.59-13140.57) | -0.1 (-0.12 to -0.08) | <0.001 |
| Kenya | 2519052  (1922826-3065236) | 19549.03  (15624.37-23278.62) | 6444966  (5052434-7846619) | 17924.98  (14518.47-21122.1) | -0.28 (-0.31 to -0.24) | <0.001 |
| Kiribati | 4419  (3160-5789) | 9255.9  (6780.74-11898.33) | 1728  (1189-2482) | 1852.61  (1311.99-2595.58) | -5.03 (-5.57 to -4.49) | <0.001 |
| Kuwait | 179008  (128735-233557) | 13424.85  (10423.91-16688.19) | 808330  (617318-1042828) | 14191.16  (11427.03-17510.29) | 0.19 (0.16 to 0.21) | <0.001 |
| Kyrgyzstan | 315904  (233908-404898) | 9702.98  (7222.64-12380.9) | 482442  (349760-645597) | 7987.93  (5858.9-10517.76) | -0.64 (-0.69 to -0.58) | <0.001 |
| Lao People's Democratic Republic | 99513  (68949-133648) | 3971.91  (2787.23-5308.07) | 338619  (232034-459101) | 5725.25  (3992.56-7882.92) | 1.18 (1.04 to 1.32) | <0.001 |
| Latvia | 374299  (285468-460138) | 11387.4  (8578.07-14101.06) | 325777  (254100-395586) | 11514.81  (8877.53-14745.54) | 0.04 (0.01 to 0.07) | 0.004 |
| Lebanon | 258757  (189039-332715) | 10419.23  (7714.01-13311.75) | 710802  (533485-932773) | 11747.69  (8862.15-15236.77) | 0.38 (0.29 to 0.47) | <0.001 |
| Lesotho | 39680  (27284-53052) | 4064.26  (2831.84-5406.45) | 68995  (47379-92696) | 5077.2  (3522.27-6710.59) | 0.72 (0.69 to 0.75) | <0.001 |
| Liberia | 369788  (293232-437678) | 22390.07  (18341.75-26140.98) | 871728  (678240-1055949) | 21452.82  (17238.16-25640.83) | -0.13 (-0.15 to -0.12) | <0.001 |
| Libya | 322828  (237490-407612) | 12596.98  (9560.63-15721.07) | 834968  (609499-1121239) | 11117.81  (8485.87-14430.48) | -0.41 (-0.44 to -0.38) | <0.001 |
| Lithuania | 485316  (371108-600775) | 11379.46  (8592.97-14091.96) | 481974  (383993-582910) | 11667.69  (8992.31-14859.89) | 0.09 (0.03 to 0.14) | 0.004 |
| Luxembourg | 52812  (38713-67254) | 10960.69  (7998.77-13978.61) | 90680  (67604-114976) | 10069.06  (7442.34-13082.67) | -0.3 (-0.38 to -0.22) | <0.001 |
| Madagascar | 1428371  (1088887-1750188) | 19409.4  (15242.13-23352.72) | 711061  (494250-1011469) | 4114.98  (2961.49-5563.75) | -4.9 (-5.14 to -4.67) | <0.001 |
| Malawi | 1087189  (813383-1336034) | 18535.06  (14484.61-22232.17) | 1057443  (749779-1506386) | 9434.8  (6871.46-12569.72) | -2.1 (-2.16 to -2.04) | <0.001 |
| Malaysia | 1428887  (1016278-1859244) | 11157.04  (8231.96-14169.62) | 2488547  (1849045-3271224) | 7822.4  (5895.31-10193.77) | -1.16 (-1.27 to -1.05) | <0.001 |
| Maldives | 13386  (9619-17331) | 10965.26  (8044.34-13972.97) | 38396  (27295-53524) | 7736.85  (5765.69-10135.77) | -1.1 (-1.21 to -0.99) | <0.001 |
| Mali | 1246437  (983682-1490873) | 21935.93  (17518.68-25871.62) | 3371385  (2637762-4022981) | 22789.19  (18425.69-26380.69) | 0.12 (0.1 to 0.14) | <0.001 |
| Malta | 40710  (29285-52249) | 9646.6  (6918.33-12356.52) | 61176  (46915-77774) | 9173.83  (6825.99-11875.82) | -0.17 (-0.34 to -0.01) | 0.036 |
| Marshall Islands | 2183  (1559-2862) | 9836.6  (7194.17-12528.41) | 975  (672-1366) | 2097.55  (1518.57-2875.72) | -4.85 (-5.1 to -4.61) | <0.001 |
| Mauritania | 321952  (263554-379022) | 22908.08  (19074.1-26366.73) | 569770  (448060-699364) | 18475.1  (14663.03-22135.88) | -0.73 (-0.8 to -0.67) | <0.001 |
| Mauritius | 101496  (73458-131158) | 10920.55  (8102.63-13859.52) | 132063  (96636-170977) | 7503.25  (5539.32-9751.06) | -1.19 (-1.28 to -1.1) | <0.001 |
| Mexico | 8677622  (6521643-10737409) | 14753.12  (11366.88-17953.31) | 19952694  (16658934-23108434) | 14488.05  (12140.27-16738.47) | -0.05 (-0.06 to -0.05) | <0.001 |
| Micronesia (Federated States of) | 5651  (4128-7371) | 9605.37  (6989.31-12404.43) | 1835  (1272-2613) | 2050.38  (1462.86-2850.73) | -4.86 (-5.1 to -4.63) | <0.001 |
| Monaco | 6259  (4887-7547) | 13861.94  (10517.4-17071.38) | 7319  (5824-8831) | 12175.54  (9222.2-15725.37) | -0.41 (-0.46 to -0.35) | <0.001 |
| Mongolia | 114935  (82407-148852) | 9025.56  (6510.78-11544.72) | 294869  (212749-397811) | 9294.96  (6905-12031.3) | 0.1 (0.06 to 0.14) | <0.001 |
| Montenegro | 59309  (43593-75668) | 9091.17  (6693.59-11562.28) | 78581  (59506-99766) | 9343.11  (6998.12-12130.96) | 0.09 (0.06 to 0.13) | <0.001 |
| Morocco | 2679507  (2020952-3391520) | 14564.97  (11279.31-17961.65) | 5470005  (4294194-6716681) | 14090.57  (11111.16-17199.93) | -0.09 (-0.16 to -0.02) | 0.009 |
| Mozambique | 1447272  (1083946-1792917) | 17095.46  (13024.25-20794.54) | 1628725  (1172516-2260738) | 9664.63  (7102.96-12822.7) | -1.77 (-1.86 to -1.68) | <0.001 |
| Myanmar | 2362372  (1655496-3125152) | 8397.65  (5960.24-11055.21) | 3646236  (2617287-4849842) | 6796.24  (5007.63-8901.86) | -0.64 (-0.81 to -0.47) | <0.001 |
| Namibia | 45388  (31561-61470) | 5536.18  (3862.57-7402.15) | 117595  (80420-158840) | 6479.18  (4566.04-8739.46) | 0.55 (0.46 to 0.64) | <0.001 |
| Nauru | 831  (606-1046) | 12549.91  (9497.76-15367.95) | 203  (137-291) | 2670.96  (1907.81-3732.11) | -4.86 (-5.29 to -4.43) | <0.001 |
| Nepal | 1541527  (1078209-2013395) | 11828.48  (8420.83-15399.15) | 3504886  (2532662-4633497) | 12794.29  (9304.06-16684.77) | 0.23 (0.17 to 0.29) | <0.001 |
| Netherlands | 1764653  (1267891-2285558) | 9977.43  (7101.06-13003.99) | 2179196  (1631693-2819983) | 8875.58  (6526.53-11683.74) | -0.33 (-0.47 to -0.2) | <0.001 |
| New Zealand | 324743  (232402-428356) | 8897.09  (6297.04-11781.43) | 843241  (669930-1021498) | 13215.76  (10381.64-16263.35) | 1.26 (1.13 to 1.4) | <0.001 |
| Nicaragua | 280406  (205459-358970) | 12849.67  (9730.87-16098.17) | 775270  (585604-994726) | 12759.43  (9691.08-16096.94) | -0.01 (-0.04 to 0.01) | 0.196 |
| Niger | 1035759  (810266-1255142) | 21576.44  (17365.48-25665.33) | 1008498  (717977-1422844) | 7877.84  (5631.26-10894) | -3.18 (-3.57 to -2.78) | <0.001 |
| Nigeria | 13111777  (10549660-15623482) | 21200.75  (17404.83-24791.87) | 7420694  (5185844-10490209) | 5477.24  (3934.71-7447.35) | -4.09 (-4.39 to -3.79) | <0.001 |
| Niue | 232  (175-288) | 11330.38  (8529.42-14119.92) | 57  (40-79) | 2757.22  (1962.85-3761.21) | -4.42 (-4.65 to -4.19) | <0.001 |
| North Macedonia | 171969  (122602-224977) | 8411.76  (6031.2-10916.25) | 277591  (206270-355661) | 9029.94  (6712.2-11727.12) | 0.23 (0.21 to 0.25) | <0.001 |
| Northern Mariana Islands | 5154  (3760-6593) | 14172.64  (11194.6-17318) | 1922  (1310-2686) | 3275.42  (2339.05-4490.96) | -4.7 (-5.06 to -4.34) | <0.001 |
| Norway | 854889  (689294-1016551) | 17159.46  (13617.9-20580.72) | 1117576  (912863-1313953) | 15892.36  (12754.44-19007.1) | -0.24 (-0.39 to -0.09) | 0.002 |
| Oman | 149197  (104542-194274) | 11871.72  (8956.08-14879.08) | 615681  (435481-838583) | 13277.47  (10341.64-16664.3) | 0.37 (0.33 to 0.4) | <0.001 |
| Pakistan | 15832696  (12970438-18675285) | 20943.58  (17496.01-24324.8) | 40838387  (33813785-47244425) | 21518.1  (17984.43-24469.51) | 0.08 (0.07 to 0.1) | <0.001 |
| Palau | 1428  (1059-1810) | 11763.39  (8915.64-14606.22) | 699  (485-975) | 2818.51  (2037.02-3884.8) | -4.51 (-4.7 to -4.31) | <0.001 |
| Palestine | 91166  (65923-119061) | 8429.04  (6011.21-10977.69) | 375812  (271231-510139) | 10054.43  (7525.33-13079.91) | 0.57 (0.51 to 0.64) | <0.001 |
| Panama | 264246  (198258-330866) | 14265.19  (10925.49-17586.87) | 674373  (539002-820863) | 15318.19  (12241.1-18665.07) | 0.23 (0.21 to 0.25) | <0.001 |
| Papua New Guinea | 239009  (169006-312470) | 9574.57  (6934.15-12269.35) | 150396  (104033-217156) | 2084.86  (1493.01-2894.35) | -4.8 (-5.04 to -4.55) | <0.001 |
| Paraguay | 338010  (249309-429696) | 12275.68  (9349.23-15408.05) | 819556  (611709-1063181) | 12063.32  (9135.15-15309.06) | -0.05 (-0.08 to -0.02) | 0.001 |
| Peru | 1786366  (1462276-2150293) | 11296.35  (9266.44-13443.2) | 4291796  (3315285-5484064) | 11524.05  (8910.63-14632.44) | 0.08 (0.04 to 0.12) | <0.001 |
| Philippines | 4589974  (3313682-5883198) | 11147.53  (8329.17-14035.75) | 3805439  (3031750-4688831) | 3861.98  (3137.75-4734.66) | -3.35 (-3.64 to -3.05) | <0.001 |
| Poland | 4738548  (3584710-5974676) | 11179.01  (8450.34-14110.7) | 6413850  (5057860-7872762) | 11915.65  (9297.5-15064.3) | 0.2 (0.12 to 0.27) | <0.001 |
| Portugal | 1123597  (810563-1452016) | 9355.86  (6711.49-12146) | 1413273  (1062822-1829113) | 8510.96  (6168.35-11169.41) | -0.32 (-0.5 to -0.15) | <0.001 |
| Puerto Rico | 619250  (496476-742252) | 17252.61  (13844.27-20682.25) | 729777  (603641-853169) | 16239.66  (12995.22-19595.53) | -0.21 (-0.26 to -0.16) | <0.001 |
| Qatar | 55787  (39849-73104) | 14804.3  (11543.63-18037.49) | 543947  (399867-706825) | 15350.7  (12368.83-18705.71) | 0.12 (0.09 to 0.15) | <0.001 |
| Republic of Korea | 2816770  (2005598-3672442) | 7503.01  (5413.41-9733.98) | 6953477  (5220189-8693852) | 8288.53  (6261.58-10626.59) | 0.32 (0.25 to 0.4) | <0.001 |
| Republic of Moldova | 450767  (329150-575455) | 9830.81  (7153.59-12529.63) | 485262  (355226-619602) | 9229.87  (6738.3-11892.63) | -0.19 (-0.29 to -0.1) | <0.001 |
| Romania | 2356169  (1728668-3047474) | 8736.69  (6366.62-11328.25) | 2766675  (2119248-3458630) | 9771.63  (7345.24-12628.46) | 0.38 (0.3 to 0.46) | <0.001 |
| Russian Federation | 21001159  (16201932-26074903) | 11982.24  (9182.28-14923.28) | 22296855  (17493528-27588890) | 11033.49  (8500.07-14068.51) | -0.27 (-0.29 to -0.25) | <0.001 |
| Rwanda | 800248  (597631-986601) | 18688.06  (14593.87-22552.3) | 961840  (671185-1337343) | 10523.34  (7609.66-13895.71) | -1.79 (-1.89 to -1.68) | <0.001 |
| Saint Kitts and Nevis | 5341  (4202-6522) | 16133.27  (12595.28-19699.8) | 11685  (9288-14230) | 15580.96  (12350.46-19060.01) | -0.11 (-0.18 to -0.05) | 0.001 |
| Saint Lucia | 15258  (11448-18787) | 15504.71  (11923.21-18884.03) | 33010  (25887-40367) | 14470.8  (11205.6-17743.32) | -0.22 (-0.28 to -0.15) | <0.001 |
| Saint Vincent and the Grenadines | 11512  (8669-14188) | 14829.56  (11233.97-18039.22) | 19071  (14942-23269) | 14275.12  (11184.81-17725.34) | -0.13 (-0.23 to -0.04) | 0.007 |
| Samoa | 10340  (7528-13235) | 10239.37  (7560.76-12972.68) | 3781  (2646-5162) | 2312.24  (1637.23-3130.93) | -4.67 (-4.9 to -4.45) | <0.001 |
| San Marino | 3318  (2493-4157) | 11454.55  (8489.69-14533.74) | 4871  (3705-6184) | 9549.86  (7171.28-12326.5) | -0.59 (-0.68 to -0.51) | <0.001 |
| Sao Tome and Principe | 17989  (14719-21179) | 22912.74  (18981.2-26361.48) | 32362  (25498-40013) | 18659.3  (14874.65-22468.42) | -0.66 (-0.76 to -0.55) | <0.001 |
| Saudi Arabia | 698123  (579640-829078) | 7335.14  (6104.42-8816.2) | 3389776  (2286392-4872665) | 8648.01  (6304.82-11388.03) | 0.54 (0.49 to 0.6) | <0.001 |
| Senegal | 1113635  (914369-1318900) | 22948.74  (19307.23-26342.73) | 2103347  (1650642-2617244) | 18307.65  (14584.9-21781.17) | -0.73 (-0.81 to -0.65) | <0.001 |
| Serbia | 1012909  (727968-1317756) | 8821.59  (6401.61-11489.06) | 1166041  (880902-1477557) | 9124.86  (6812.2-11955.05) | 0.12 (0.09 to 0.15) | <0.001 |
| Seychelles | 7256  (5403-9173) | 12389.65  (9197.96-15602.59) | 10535  (7758-13747) | 8332.15  (6298.8-10781.42) | -1.26 (-1.35 to -1.17) | <0.001 |
| Sierra Leone | 494669  (378104-612910) | 17044.51  (12999.62-20812.49) | 1715067  (1496566-1966810) | 25737.78  (22710.32-28915.71) | 1.4 (1.35 to 1.45) | <0.001 |
| Singapore | 244777  (177594-316426) | 8725.23  (6419.81-11186.5) | 788837  (594423-1008014) | 9233.64  (7023.47-11807.32) | 0.17 (0.15 to 0.2) | <0.001 |
| Slovakia | 516471  (376042-662222) | 9007.95  (6487.86-11568.66) | 779838  (594307-981512) | 9897.37  (7416.67-12814.5) | 0.31 (0.28 to 0.34) | <0.001 |
| Slovenia | 313124  (270967-358655) | 13440.16  (11557.22-15446.51) | 402911  (323900-487532) | 13241  (10343.14-16594.07) | -0.04 (-0.08 to -0.01) | 0.025 |
| Solomon Islands | 16145  (11392-21040) | 8847.19  (6333.18-11404.72) | 8940  (6148-12919) | 1904.3  (1364.93-2644.76) | -4.79 (-5.01 to -4.57) | <0.001 |
| Somalia | 707084  (508769-902097) | 15793.3  (11861.11-19530.8) | 687293  (471761-988086) | 6488.3  (4675.16-8793.91) | -2.81 (-3 to -2.62) | <0.001 |
| South Africa | 1981086  (1420454-2585269) | 7900.98  (5663.39-10314.11) | 3142214  (2339729-4064807) | 5813.61  (4409.05-7435.73) | -0.99 (-1.14 to -0.83) | <0.001 |
| South Sudan | 787716  (628728-944331) | 21366  (17502-24952.79) | 596854  (421789-826297) | 10325.43  (7483.65-13521.57) | -2.3 (-2.45 to -2.15) | <0.001 |
| Spain | 4472828  (3229558-5788089) | 9828.45  (7082.98-12829.35) | 1689391  (1217257-2332685) | 2313.87  (1637.21-3183.57) | -4.64 (-5.05 to -4.23) | <0.001 |
| Sri Lanka | 2525411  (1977790-3048689) | 17405.43  (14014.74-20645.34) | 2022140  (1481668-2658731) | 7578.52  (5580.01-9984.05) | -2.71 (-2.86 to -2.55) | <0.001 |
| Sudan | 926022  (648730-1218616) | 7837.14  (5481.21-10244.68) | 2555376  (1823065-3380797) | 8883.47  (6328.31-11500.9) | 0.41 (0.39 to 0.43) | <0.001 |
| Suriname | 49650  (37927-61074) | 15859.59  (12292.04-19314.92) | 93896  (73253-113455) | 14671.7  (11445.29-17833.24) | -0.25 (-0.33 to -0.17) | <0.001 |
| Sweden | 1387896  (1063964-1715601) | 12343.42  (9296.54-15504.21) | 1551095  (1201077-1877130) | 10635.83  (7919.23-13492.33) | -0.35 (-0.55 to -0.14) | 0.001 |
| Switzerland | 965548  (725290-1203205) | 11095.02  (8207.31-13929.01) | 1305039  (995939-1644448) | 9849.69  (7322.47-12773.34) | -0.4 (-0.54 to -0.25) | <0.001 |
| Syrian Arab Republic | 623545  (449043-814009) | 8970.04  (6523.56-11562.39) | 1478696  (1102692-1915019) | 9933.43  (7480.08-12925.67) | 0.33 (0.31 to 0.34) | <0.001 |
| Taiwan (Province of China) | 1676772  (1251015-2132917) | 9303.23  (6981.52-11819.87) | 3658322  (2748684-4587873) | 9624.81  (7098.34-12166.69) | 0.1 (0.06 to 0.14) | <0.001 |
| Tajikistan | 305586  (225009-390617) | 9580.64  (7099.67-12087.11) | 622468  (441109-846846) | 7684.29  (5566.16-10337.44) | -0.71 (-0.79 to -0.63) | <0.001 |
| Thailand | 4726488  (3708218-5867492) | 10253.88  (8197.76-12740.04) | 11829054  (9466032-14514573) | 11947.79  (9457.2-15050.11) | 0.51 (0.43 to 0.6) | <0.001 |
| Timor-Leste | 43377  (29835-58418) | 9408.12  (6785-12176.59) | 61250  (45003-82018) | 6497.28  (4783.24-8623.18) | -1.17 (-1.39 to -0.95) | <0.001 |
| Togo | 497983  (387146-592152) | 22318.27  (18233.66-26074.11) | 1067985  (810238-1333777) | 17090.58  (13244.39-20729.39) | -0.86 (-1 to -0.71) | <0.001 |
| Tokelau | 130  (97-164) | 10115.9  (7460.91-12889.58) | 35  (25-47) | 2438.26  (1730.41-3322.42) | -4.47 (-4.7 to -4.24) | <0.001 |
| Tonga | 6451  (4717-8232) | 10175.4  (7502.18-12868.28) | 1985  (1418-2722) | 2323.81  (1657.13-3178.98) | -4.67 (-4.89 to -4.45) | <0.001 |
| Trinidad and Tobago | 168072  (130470-204318) | 16519.52  (13073.36-19900.22) | 279988  (224730-333050) | 16067  (12836.31-19281.83) | -0.08 (-0.12 to -0.04) | <0.001 |
| Tunisia | 582437  (423443-745816) | 9693.27  (7099.32-12272.3) | 1611847  (1215482-2073892) | 11656.93  (8841.24-14967.96) | 0.6 (0.53 to 0.68) | <0.001 |
| Turkey | 2720408  (1911731-3659199) | 6358.9  (4459.25-8553.13) | 12670145  (9968430-15822489) | 13118.6  (10411.36-16397.61) | 2.36 (2.22 to 2.51) | <0.001 |
| Turkmenistan | 240174  (176345-309069) | 10330.91  (7719.7-13044.43) | 498871  (371763-665197) | 10143.33  (7699.12-13158.62) | -0.06 (-0.09 to -0.03) | <0.001 |
| Tuvalu | 698  (502-900) | 9097.44  (6577.45-11663.22) | 236  (166-327) | 2107.24  (1511.35-2893.35) | -4.56 (-5.13 to -3.99) | <0.001 |
| Uganda | 1773268  (1328192-2174758) | 18452.29  (14318.08-22237.92) | 2407142  (1712740-3379812) | 10505.35  (7667.25-13979.01) | -1.77 (-1.95 to -1.59) | <0.001 |
| Ukraine | 7386192  (5717539-9123949) | 11387.32  (8708.97-14118.15) | 6409560  (4889200-7913930) | 10036.23  (7486.18-12841.24) | -0.4 (-0.45 to -0.34) | <0.001 |
| United Arab Emirates | 224218  (158601-294947) | 14206.35  (10955.61-17475.18) | 2016734  (1475007-2647909) | 14163.19  (11239.65-17446.01) | 0 (-0.04 to 0.03) | 0.825 |
| United Kingdom | 4492050  (3162636-6006575) | 6229.32  (4328.48-8320.13) | 5448250  (4267900-6875270) | 5599.78  (4287.09-7118.05) | -0.41 (-0.59 to -0.24) | <0.001 |
| United Republic of Tanzania | 2998836  (2313393-3637700) | 19365.45  (15404.2-23125.49) | 4050069  (2897251-5520821) | 11032.19  (8041.32-14334.78) | -1.75 (-1.82 to -1.69) | <0.001 |
| United States of America | 28139903  (21768269-35459119) | 9816.52  (7461.93-12405.45) | 42434308  (34984165-50221307) | 9165.58  (7467.83-10986.67) | -0.15 (-0.2 to -0.09) | <0.001 |
| United States Virgin Islands | 18312  (14346-21876) | 17512.69  (13887.89-20821.2) | 20276  (16893-23564) | 16795.38  (13522.7-20203.85) | -0.22 (-0.33 to -0.12) | <0.001 |
| Uruguay | 528901  (417427-634576) | 15893.6  (12299.63-19212.38) | 681609  (555561-806843) | 16589.02  (13280.38-19929.3) | 0.14 (0.1 to 0.17) | <0.001 |
| Uzbekistan | 1265200  (902678-1624373) | 9604.65  (6927.18-12201.3) | 3001793  (2151243-3979809) | 9002.06  (6619.7-11724.37) | -0.19 (-0.22 to -0.15) | <0.001 |
| Vanuatu | 8083  (5705-10752) | 9399.07  (6880.04-12159.36) | 4407  (3147-6319) | 1966  (1413.7-2740.03) | -4.92 (-5.15 to -4.69) | <0.001 |
| Venezuela (Bolivarian Republic of) | 2052524  (1551787-2560151) | 15044.51  (11646.22-18348.54) | 4263516  (3325336-5221138) | 14022.86  (10887.48-17333.2) | -0.21 (-0.24 to -0.18) | <0.001 |
| Viet Nam | 2487918  (1754882-3308620) | 5496.64  (3873.33-7341.48) | 7737647  (5575960-10253345) | 7059.92  (5162.05-9184.69) | 0.78 (0.68 to 0.88) | <0.001 |
| Yemen | 645618  (489370-803571) | 9395.77  (7326.89-11621.33) | 2203512  (1569855-3050862) | 9971.73  (7463.84-13136.5) | 0.17 (0.11 to 0.24) | <0.001 |
| Zambia | 899152  (689309-1092157) | 19912.46  (15846.06-23749.44) | 1329335  (918538-1859577) | 11540.77  (8316.2-15102.02) | -1.69 (-2.05 to -1.32) | <0.001 |
| Zimbabwe | 269814  (187229-363408) | 5044.59  (3508.99-6688.77) | 457883  (309754-613584) | 4723.06  (3258.07-6316.86) | -0.21 (-0.25 to -0.17) | <0.001 |

AAPC: average annual percent change; ASR: age-standardized rate.
