# Supplementary material for: Global, regional, and national burden of periodontal diseases from 1990 to 2021 and predictions to 2040: an analysis of the global burden of disease study 2021
Source: Front Oral Health. 2025 Jul 24;6:1627746. doi: 10.3389/froh.2025.1627746 (PMC12332980; doi:10.3389/froh.2025.1627746)
Supplement: Supplementary file 4 [file Table3.docx]

**Supplementary Table S3** The case number and ASR of DALYs of periodontal diseases in 1990 and 2021 across 204 countries and territories, with AAPC from 1990 to 2021

| **Location** | **1990** | | **2021** | | **AAPC(95%CI)**  **1990–2021** | ***P*** |
| --- | --- | --- | --- | --- | --- | --- |
|  | **Number (95%UI)** | **ASR (95%UI)** | **Number (95%UI)** | **ASR (95%UI)** |  |  |
| Afghanistan | 3546  (1367-7863) | 49.25  (18.93-108.88) | 9179  (3607-20041) | 53.24  (21.17-112.67) | 0.27 (0.2 to 0.34) | <0.001 |
| Albania | 1249  (481-2755) | 49.01  (18.98-107.46) | 2063  (803-4243) | 57.31  (22.61-118.78) | 0.52 (0.48 to 0.56) | <0.001 |
| Algeria | 10780  (4247-23833) | 68.98  (27.5-151.9) | 33154  (13125-68525) | 75.57  (30.02-155.41) | 0.29 (0.27 to 0.31) | <0.001 |
| American Samoa | 27  (11-57) | 85.5  (34.24-179.7) | 43  (17-91) | 81.18  (32.66-171.44) | -0.16 (-0.17 to -0.16) | <0.001 |
| Andorra | 48  (19-104) | 77.03  (30.37-165.76) | 87  (34-181) | 64.67  (25.16-137.49) | -0.55 (-0.62 to -0.49) | <0.001 |
| Angola | 7631  (3055-15441) | 117.53  (47.29-239.77) | 14377  (5719-30087) | 76.58  (30.73-159.14) | -1.33 (-1.55 to -1.11) | <0.001 |
| Antigua and Barbuda | 59  (23-119) | 110.1  (44.15-223.91) | 109  (43-226) | 100.18  (39.74-209.83) | -0.3 (-0.36 to -0.24) | <0.001 |
| Argentina | 24443  (9607-53237) | 76.44  (30.09-166.45) | 40447  (15964-83189) | 78.87  (30.95-162.88) | 0.11 (0.05 to 0.17) | <0.001 |
| Armenia | 1961  (788-4289) | 63.75  (25.5-139.89) | 2305  (924-4706) | 60.11  (24.05-123.84) | -0.19 (-0.22 to -0.15) | <0.001 |
| Australia | 9688  (3731-21349) | 52.18  (20.18-114.82) | 20156  (7980-42740) | 60.26  (23.18-129.19) | 0.45 (0.23 to 0.67) | <0.001 |
| Austria | 6187  (2422-13639) | 64.75  (25.48-143.27) | 7706  (2982-16154) | 58.74  (22.98-125.13) | -0.33 (-0.43 to -0.23) | <0.001 |
| Azerbaijan | 3950  (1580-8584) | 68.45  (27.38-148.91) | 7922  (3151-16611) | 64.71  (25.5-135.11) | -0.18 (-0.24 to -0.12) | <0.001 |
| Bahamas | 247  (98-498) | 114.54  (46.04-233.39) | 457  (181-956) | 102.32  (40.29-213.91) | -0.36 (-0.4 to -0.32) | <0.001 |
| Bahrain | 315  (125-678) | 81.3  (33.04-174.53) | 1562  (614-3310) | 88.17  (34.88-183.07) | 0.27 (0.25 to 0.28) | <0.001 |
| Bangladesh | 80779  (32103-166473) | 116.37  (45.93-236.71) | 198444  (77177-403078) | 124.34  (48.72-252.2) | 0.21 (0.19 to 0.23) | <0.001 |
| Barbados | 274  (110-558) | 108.11  (43.35-220.77) | 381  (150-769) | 95.49  (37.42-196.5) | -0.41 (-0.44 to -0.38) | <0.001 |
| Belarus | 8656  (3462-18863) | 70.85  (28.19-154.25) | 9379  (3759-18933) | 69.39  (27.7-143.1) | -0.06 (-0.09 to -0.03) | <0.001 |
| Belgium | 12469  (5006-25699) | 102.5  (40.95-212.75) | 14910  (5956-30390) | 97.67  (38.91-204.08) | -0.15 (-0.26 to -0.04) | 0.007 |
| Belize | 109  (43-230) | 94.77  (37.55-197.35) | 345  (135-737) | 88.68  (35.49-187.16) | -0.21 (-0.3 to -0.12) | <0.001 |
| Benin | 4383  (1742-8740) | 146.44  (58.74-292.64) | 10164  (4002-21509) | 116.67  (46.61-243.11) | -0.72 (-0.82 to -0.62) | <0.001 |
| Bermuda | 81  (32-164) | 117.63  (46.73-238.72) | 109  (44-218) | 121.43  (48.49-243.03) | 0.1 (0.08 to 0.11) | <0.001 |
| Bhutan | 487  (192-984) | 119.66  (47.66-244.17) | 997  (387-1999) | 131.13  (50.95-259.11) | 0.3 (0.28 to 0.31) | <0.001 |
| Bolivia (Plurinational State of) | 3210  (1296-6836) | 73.01  (29.44-155.14) | 7901  (3079-16774) | 70.83  (27.43-148.07) | -0.1 (-0.17 to -0.03) | 0.005 |
| Bosnia and Herzegovina | 1935  (749-4332) | 41.06  (15.9-90.24) | 2797  (1094-5837) | 57.1  (22.56-119.78) | 1.08 (0.98 to 1.19) | <0.001 |
| Botswana | 270  (104-591) | 36.75  (14.03-79.75) | 589  (219-1336) | 28.69  (10.85-63.32) | -0.74 (-0.86 to -0.62) | <0.001 |
| Brazil | 81688  (32664-174989) | 69.72  (27.88-150.31) | 205232  (83738-428792) | 80.08  (32.71-167.56) | 0.49 (0.36 to 0.61) | <0.001 |
| Brunei Darussalam | 115  (46-253) | 70.04  (27.88-147.64) | 275  (109-565) | 58.38  (22.99-117.38) | -0.59 (-0.67 to -0.52) | <0.001 |
| Bulgaria | 5116  (1942-11011) | 45.04  (17.27-97.35) | 5042  (1927-10671) | 47.13  (18.13-99.68) | 0.15 (0.05 to 0.24) | 0.002 |
| Burkina Faso | 8581  (3393-17090) | 144.18  (57.61-288.2) | 22324  (8835-44720) | 147.69  (59.41-293.79) | 0.08 (0.06 to 0.09) | <0.001 |
| Burundi | 4117  (1665-8443) | 121.35  (48.75-247.1) | 4282  (1632-9619) | 56.09  (21.44-121.2) | -2.45 (-2.52 to -2.38) | <0.001 |
| Cabo Verde | 361  (144-726) | 151.2  (60.88-301.19) | 920  (360-1853) | 160.37  (63.21-320.55) | 0.19 (0.18 to 0.2) | <0.001 |
| Cambodia | 3118  (1215-6899) | 52.25  (20.4-115.05) | 9882  (3822-21569) | 64.82  (24.98-141.4) | 0.7 (0.67 to 0.74) | <0.001 |
| Cameroon | 10569  (4219-21173) | 153.02  (61.7-302.7) | 34408  (13630-69892) | 151.4  (60.73-305.26) | -0.03 (-0.04 to -0.03) | <0.001 |
| Canada | 29189  (11845-60986) | 93.46  (37.99-195.82) | 47562  (18670-95608) | 90.99  (36.06-185.13) | -0.08 (-0.11 to -0.06) | <0.001 |
| Central African Republic | 1805  (720-3782) | 103.13  (40.7-212.58) | 1808  (689-3996) | 52.36  (20.35-113.3) | -2.16 (-2.24 to -2.09) | <0.001 |
| Chad | 5399  (2132-10719) | 141.02  (56.69-281.35) | 11087  (4306-23664) | 112  (43.97-235.56) | -0.77 (-0.89 to -0.65) | <0.001 |
| Chile | 9608  (3766-20594) | 81.63  (31.78-174.47) | 21023  (8289-43309) | 92.2  (36.25-191.05) | 0.38 (0.24 to 0.51) | <0.001 |
| China | 724760  (287789-1563001) | 72.13  (28.83-156.29) | 1428453  (563791-2917317) | 70.15  (27.97-144.15) | -0.09 (-0.28 to 0.11) | 0.377 |
| Colombia | 22635  (8981-47330) | 92.85  (37.17-193.93) | 51677  (20551-107661) | 94.82  (37.75-197.99) | 0.06 (0.04 to 0.08) | <0.001 |
| Comoros | 380  (147-760) | 131.76  (52.36-259.75) | 436  (170-924) | 69.88  (27.29-147.1) | -1.99 (-2.03 to -1.95) | <0.001 |
| Congo | 1840  (731-3680) | 119.56  (48.03-241.08) | 3074  (1202-6520) | 74.19  (29.21-154.8) | -1.54 (-1.7 to -1.38) | <0.001 |
| Cook Islands | 12  (5-25) | 80.29  (32.59-171.32) | 5  (2-11) | 21.56  (8.07-47.45) | -4.19 (-4.37 to -4.01) | <0.001 |
| Costa Rica | 2120  (841-4412) | 93.28  (37.53-195.16) | 5131  (2032-10584) | 94.63  (37.44-195.44) | 0.04 (0.01 to 0.07) | 0.003 |
| Coted'Ivoire | 12036  (4871-24290) | 150.45  (61.28-304.01) | 24383  (9530-51277) | 120.57  (47.42-246.54) | -0.71 (-0.77 to -0.65) | <0.001 |
| Croatia | 4234  (1703-8689) | 70.56  (28.47-145.85) | 5064  (2007-10331) | 82.45  (32.89-170.66) | 0.49 (0.43 to 0.55) | <0.001 |
| Cuba | 10918  (4373-22420) | 101.42  (40.75-209.44) | 13907  (5614-28526) | 90.12  (36.39-187.38) | -0.38 (-0.43 to -0.33) | <0.001 |
| Cyprus | 527  (203-1181) | 64.69  (25.09-144.69) | 1050  (403-2206) | 57.13  (21.89-120.35) | -0.41 (-0.5 to -0.32) | <0.001 |
| Czechia | 7851  (3068-16895) | 63.18  (25.01-135.6) | 10459  (4170-20968) | 66.46  (26.11-135.74) | 0.18 (0.1 to 0.26) | <0.001 |
| Democratic People's Republic of Korea | 8990  (3413-19247) | 47.88  (18.25-101.14) | 14543  (5579-31264) | 42.59  (16.61-90.73) | -0.38 (-0.42 to -0.34) | <0.001 |
| Democratic Republic of the Congo | 25550  (10206-52494) | 108.93  (43.8-223.45) | 30215  (11578-67458) | 54.6  (20.81-119.03) | -2.2 (-2.33 to -2.06) | <0.001 |
| Denmark | 8922  (3618-17981) | 143.88  (58.2-290.89) | 10161  (3975-19996) | 136.17  (53.79-272.21) | -0.18 (-0.22 to -0.14) | <0.001 |
| Djibouti | 368  (144-749) | 136.87  (54.89-272.91) | 781  (301-1640) | 75.78  (29.25-159.02) | -1.88 (-1.91 to -1.84) | <0.001 |
| Dominica | 58  (23-119) | 99.06  (40.14-205.95) | 72  (29-150) | 92.12  (36.53-192.98) | -0.24 (-0.28 to -0.2) | <0.001 |
| Dominican Republic | 4789  (1911-10137) | 94.21  (37.65-198.04) | 10350  (4110-21569) | 93.71  (37.26-194.79) | -0.01 (-0.06 to 0.03) | 0.575 |
| Ecuador | 5724  (2341-11762) | 79.85  (32.05-162.29) | 13859  (5494-29550) | 78.32  (30.95-166.53) | -0.04 (-0.11 to 0.03) | 0.229 |
| Egypt | 22375  (8617-49903) | 59.16  (22.64-129.36) | 64505  (25272-136431) | 72.98  (28.62-152.51) | 0.68 (0.62 to 0.74) | <0.001 |
| El Salvador | 2963  (1168-6386) | 82.68  (32.44-180.06) | 5325  (2104-11091) | 86.17  (34.13-179.33) | 0.14 (0.12 to 0.16) | <0.001 |
| Equatorial Guinea | 292  (116-604) | 108.98  (43.34-223.11) | 849  (335-1846) | 90.43  (35.68-187.85) | -0.57 (-0.91 to -0.23) | 0.001 |
| Eritrea | 2463  (981-5078) | 120.58  (48.24-244.78) | 2924  (1140-6411) | 65.22  (25.91-138.45) | -1.98 (-2.14 to -1.81) | <0.001 |
| Estonia | 1353  (541-2883) | 71.36  (28.61-153.13) | 1407  (563-2814) | 74.16  (29.51-150.57) | 0.14 (0.1 to 0.18) | <0.001 |
| Eswatini | 146  (57-326) | 36.3  (13.96-80.06) | 326  (128-723) | 41.38  (16.04-89.56) | 0.42 (0.4 to 0.45) | <0.001 |
| Ethiopia | 33377  (13447-68382) | 112.48  (45.35-228.84) | 82157  (33052-167433) | 115.52  (46.7-233.38) | 0.09 (0.08 to 0.1) | <0.001 |
| Fiji | 368  (146-793) | 70.55  (27.76-152.11) | 147  (54-322) | 16.6  (6.21-36.41) | -4.52 (-4.74 to -4.3) | <0.001 |
| Finland | 5235  (2075-11156) | 85.45  (34.19-180.83) | 6706  (2644-13622) | 88.01  (34.56-180.29) | 0.12 (0.02 to 0.22) | 0.018 |
| France | 29549  (11584-60816) | 42.67  (16.81-87.63) | 43886  (16627-95000) | 45.95  (17.49-97.26) | 0.23 (-0.01 to 0.46) | 0.056 |
| Gabon | 938  (369-1863) | 133.49  (53.02-265.82) | 1227  (480-2625) | 86.35  (33.98-180.64) | -1.41 (-1.47 to -1.35) | <0.001 |
| Gambia | 1059  (405-2153) | 165.43  (65.91-330.28) | 2830  (1095-5751) | 163.66  (65.18-327.93) | -0.03 (-0.06 to 0) | 0.044 |
| Georgia | 4243  (1700-9163) | 69.67  (27.81-151.21) | 2975  (1194-6025) | 62.09  (24.9-127.42) | -0.37 (-0.41 to -0.33) | <0.001 |
| Germany | 108374  (43400-220173) | 106.6  (42.82-215.78) | 128271  (51476-257839) | 107.16  (42.5-222.34) | -0.02 (-0.18 to 0.13) | 0.78 |
| Ghana | 15370  (6199-30862) | 150.04  (61.2-300.92) | 43042  (17089-86417) | 156.01  (63.09-310.36) | 0.13 (0.12 to 0.14) | <0.001 |
| Greece | 7433  (2874-16342) | 57.63  (22.23-128.29) | 8932  (3499-18388) | 57.12  (22.35-120.27) | -0.01 (-0.09 to 0.08) | 0.898 |
| Greenland | 36  (14-81) | 73.01  (29.1-158.25) | 51  (20-105) | 72.77  (28.85-153.52) | -0.01 (-0.04 to 0.01) | 0.32 |
| Grenada | 63  (25-130) | 96.76  (39.39-201.69) | 111  (43-232) | 94.68  (37.08-199.25) | -0.08 (-0.11 to -0.04) | <0.001 |
| Guam | 101  (40-209) | 91.56  (36.7-189.92) | 46  (17-102) | 23.68  (8.9-51.38) | -4.26 (-4.4 to -4.13) | <0.001 |
| Guatemala | 4113  (1641-8815) | 83.97  (33.46-179.57) | 11248  (4360-23813) | 84.25  (33.34-176.56) | 0.01 (-0.01 to 0.03) | 0.485 |
| Guinea | 6222  (2514-12356) | 148.04  (59.76-295.52) | 13312  (5272-26796) | 146.79  (58.97-292.45) | -0.03 (-0.04 to -0.01) | 0.001 |
| Guinea-Bissau | 922  (370-1845) | 144.63  (58.18-284.63) | 1516  (596-3335) | 110.99  (43.79-234.16) | -0.86 (-0.97 to -0.74) | <0.001 |
| Guyana | 495  (196-1031) | 90.18  (36.02-187.76) | 657  (257-1385) | 88.44  (34.81-185.61) | -0.06 (-0.11 to -0.02) | 0.009 |
| Haiti | 3679  (1473-7819) | 83.77  (33.12-180.01) | 7474  (2887-15968) | 70.1  (27.36-146.93) | -0.57 (-0.63 to -0.51) | <0.001 |
| Honduras | 2274  (905-4821) | 81.91  (32.45-174.23) | 6708  (2600-14050) | 80.86  (31.54-166.87) | -0.05 (-0.07 to -0.03) | <0.001 |
| Hungary | 3578  (1366-7837) | 26.99  (10.27-59.09) | 4473  (1651-10136) | 29.9  (11.28-65.87) | 0.35 (0.19 to 0.52) | <0.001 |
| Iceland | 167  (67-342) | 63.88  (25.57-131.88) | 273  (105-578) | 60.02  (23.3-128.34) | -0.19 (-0.24 to -0.13) | <0.001 |
| India | 658055  (266415-1349121) | 100.98  (40.72-207.93) | 1540187  (632990-3120732) | 109.07  (44.92-219.72) | 0.23 (0.18 to 0.28) | <0.001 |
| Indonesia | 117028  (46704-245615) | 85.96  (34.12-180.89) | 268117  (106780-566039) | 88.94  (35.44-187.06) | 0.12 (0.1 to 0.14) | <0.001 |
| Iran (Islamic Republic of) | 27658  (10934-58313) | 79.55  (31.71-168.27) | 77418  (31278-162817) | 80.29  (32.35-167.97) | 0.02 (-0.02 to 0.07) | 0.281 |
| Iraq | 7179  (2822-15727) | 67.83  (27.14-146.78) | 26113  (10298-55172) | 75.7  (29.91-157.9) | 0.37 (0.3 to 0.44) | <0.001 |
| Ireland | 1176  (451-2603) | 32.49  (12.59-72.26) | 2353  (883-5075) | 35.92  (13.71-77.1) | 0.3 (0.2 to 0.39) | <0.001 |
| Israel | 2948  (1146-6478) | 64.57  (25.33-141.66) | 5820  (2278-12483) | 56.31  (21.92-121.45) | -0.42 (-0.55 to -0.3) | <0.001 |
| Italy | 48616  (18908-104393) | 65.89  (25.79-142.98) | 58507  (23128-117756) | 60.67  (23.9-125.7) | -0.28 (-0.32 to -0.24) | <0.001 |
| Jamaica | 1776  (708-3747) | 98.29  (39.51-206.08) | 2778  (1109-5855) | 89.9  (35.89-189.28) | -0.29 (-0.4 to -0.18) | <0.001 |
| Japan | 96782  (38181-208944) | 58.91  (23.25-128.69) | 128797  (50996-254615) | 59.78  (23.5-121.91) | 0.04 (-0.11 to 0.2) | 0.567 |
| Jordan | 1327  (523-2913) | 64.75  (25.62-140.35) | 7944  (3086-16441) | 72.3  (28.22-149.02) | 0.36 (0.33 to 0.4) | <0.001 |
| Kazakhstan | 9833  (3899-21153) | 68.41  (27.15-148.58) | 13367  (5322-27667) | 66.26  (26.43-136.49) | -0.11 (-0.13 to -0.08) | <0.001 |
| Kenya | 16471  (6486-32935) | 126.88  (50.3-253.35) | 42084  (16508-88646) | 116.14  (45.59-236.06) | -0.28 (-0.34 to -0.23) | <0.001 |
| Kiribati | 29  (11-63) | 59.56  (23.21-130.89) | 11  (4-24) | 11.83  (4.45-25.64) | -5.06 (-5.54 to -4.57) | <0.001 |
| Kuwait | 1177  (461-2483) | 87.4  (34.51-182.89) | 5264  (2065-11225) | 91.65  (36.7-188.68) | 0.16 (0.14 to 0.18) | <0.001 |
| Kyrgyzstan | 2050  (816-4455) | 62.84  (24.62-135.53) | 3136  (1235-6691) | 51.76  (20.46-110.88) | -0.63 (-0.67 to -0.59) | <0.001 |
| Lao People's Democratic Republic | 648  (249-1414) | 25.68  (9.56-56.6) | 2211  (845-4889) | 37.11  (14.03-79.71) | 1.19 (1 to 1.37) | <0.001 |
| Latvia | 2404  (972-5114) | 73.32  (29.69-156.3) | 2082  (822-4141) | 74.18  (29.43-151.96) | 0.04 (0.01 to 0.07) | 0.004 |
| Lebanon | 1676  (671-3617) | 67.26  (26.79-145.07) | 4560  (1817-9466) | 75.32  (29.86-155.39) | 0.37 (0.32 to 0.41) | <0.001 |
| Lesotho | 257  (98-568) | 26.23  (9.91-57.88) | 441  (171-961) | 32.26  (12.34-70.14) | 0.67 (0.64 to 0.7) | <0.001 |
| Liberia | 2396  (957-4820) | 144.26  (58.23-288.46) | 5669  (2222-11498) | 138.37  (55.69-276.77) | -0.13 (-0.15 to -0.11) | <0.001 |
| Libya | 2108  (831-4483) | 81.82  (32.38-174.65) | 5392  (2152-11300) | 71.41  (28.33-147.61) | -0.44 (-0.48 to -0.41) | <0.001 |
| Lithuania | 3124  (1255-6653) | 73.37  (29.58-156.41) | 3076  (1229-6147) | 75.06  (29.73-153.76) | 0.08 (0.01 to 0.16) | 0.032 |
| Luxembourg | 342  (134-745) | 71.19  (27.96-155.78) | 585  (230-1221) | 65.16  (25.62-138.42) | -0.31 (-0.39 to -0.22) | <0.001 |
| Madagascar | 9317  (3690-18818) | 125.89  (50.3-254.72) | 4614  (1749-9995) | 26.44  (9.87-56.82) | -4.91 (-5.12 to -4.7) | <0.001 |
| Malawi | 7077  (2783-14178) | 119.91  (47.81-244.32) | 6855  (2606-15367) | 60.61  (23.67-131.77) | -2.15 (-2.2 to -2.1) | <0.001 |
| Malaysia | 9349  (3735-20434) | 72.59  (28.79-156.24) | 16075  (6240-34736) | 50.36  (19.58-107.79) | -1.19 (-1.3 to -1.08) | <0.001 |
| Maldives | 87  (34-191) | 71.19  (27.3-152.5) | 250  (96-543) | 49.86  (19.1-105.12) | -1.08 (-1.17 to -0.99) | <0.001 |
| Mali | 8137  (3194-16445) | 142.4  (56.55-287.91) | 22104  (8739-43584) | 148.28  (59.65-295.27) | 0.13 (0.11 to 0.16) | <0.001 |
| Malta | 265  (103-582) | 62.8  (24.57-137.54) | 393  (153-809) | 59.34  (23.22-124.47) | -0.21 (-0.29 to -0.13) | <0.001 |
| Marshall Islands | 14  (6-31) | 63.62  (25.1-137.41) | 6  (2-13) | 13.36  (4.94-29.43) | -4.89 (-5.12 to -4.67) | <0.001 |
| Mauritania | 2113  (846-4275) | 149.59  (60.88-300.34) | 3730  (1462-7684) | 120.38  (47.41-246.03) | -0.73 (-0.81 to -0.66) | <0.001 |
| Mauritius | 661  (257-1434) | 70.82  (27.99-151.98) | 843  (322-1812) | 48  (18.44-103.21) | -1.24 (-1.32 to -1.15) | <0.001 |
| Mexico | 56539  (22673-116879) | 95.61  (38.76-198.43) | 129452  (52809-261897) | 93.9  (38.21-189.96) | -0.05 (-0.06 to -0.05) | <0.001 |
| Micronesia (Federated States of) | 37  (14-81) | 62.11  (24.16-134.55) | 12  (4-25) | 13.12  (4.92-28.17) | -4.89 (-5.1 to -4.68) | <0.001 |
| Monaco | 41  (16-86) | 90.58  (36.07-190) | 47  (19-94) | 78.95  (31.31-163.74) | -0.42 (-0.45 to -0.39) | <0.001 |
| Mongolia | 748  (294-1641) | 58.52  (22.81-127.5) | 1911  (754-4061) | 59.99  (23.8-125.25) | 0.09 (0.03 to 0.15) | 0.004 |
| Montenegro | 385  (150-842) | 59.03  (22.83-128.38) | 504  (197-1028) | 60.2  (23.53-123.64) | 0.07 (0.03 to 0.1) | <0.001 |
| Morocco | 17474  (6955-36374) | 94.6  (37.49-195.7) | 35335  (14290-74295) | 90.86  (36.72-190.48) | -0.12 (-0.19 to -0.06) | <0.001 |
| Mozambique | 9370  (3714-19702) | 110.01  (43.35-227.38) | 10467  (3969-23165) | 61.53  (23.96-131.6) | -1.81 (-1.91 to -1.7) | <0.001 |
| Myanmar | 15351  (5852-33979) | 54.22  (20.77-117.47) | 23417  (8835-50770) | 43.45  (16.58-94.57) | -0.68 (-0.78 to -0.58) | <0.001 |
| Namibia | 295  (112-648) | 35.76  (13.42-78.25) | 760  (291-1649) | 41.62  (15.93-89.53) | 0.53 (0.44 to 0.63) | <0.001 |
| Nauru | 5  (2-12) | 81.35  (32.71-173.33) | 1  (0-3) | 17.04  (6.4-37.88) | -4.89 (-5.18 to -4.59) | <0.001 |
| Nepal | 9977  (3904-21969) | 76.03  (29.49-166.71) | 22540  (8622-47991) | 81.96  (31.44-172.09) | 0.24 (0.15 to 0.33) | <0.001 |
| Netherlands | 11515  (4478-25610) | 65.2  (25.42-145.61) | 14040  (5493-29316) | 57.54  (22.54-121.32) | -0.36 (-0.48 to -0.25) | <0.001 |
| New Zealand | 2091  (802-4695) | 57.35  (22.11-129.05) | 5449  (2173-11301) | 85.77  (34.01-180.8) | 1.29 (1.15 to 1.42) | <0.001 |
| Nicaragua | 1830  (727-3806) | 83.48  (32.95-175.4) | 5028  (1999-10763) | 82.43  (32.81-174.18) | -0.04 (-0.05 to -0.02) | <0.001 |
| Niger | 6789  (2647-13674) | 140.43  (56.16-281.85) | 6556  (2516-14088) | 50.8  (19.45-107.46) | -3.21 (-3.49 to -2.94) | <0.001 |
| Nigeria | 85484  (33806-172261) | 137.47  (54.94-273.35) | 47956  (18307-105272) | 35.11  (13.27-75.76) | -4.3 (-4.55 to -4.05) | <0.001 |
| Niue | 1  (1-3) | 73.47  (29.37-158.18) | 0  (0-1) | 17.6  (6.59-38.41) | -4.46 (-4.7 to -4.21) | <0.001 |
| North Macedonia | 1116  (428-2433) | 54.48  (20.89-118.77) | 1784  (697-3706) | 58.15  (22.87-122.13) | 0.21 (0.2 to 0.23) | <0.001 |
| Northern Mariana Islands | 34  (13-71) | 92.41  (37.48-195.86) | 12  (5-27) | 21.03  (7.84-45.46) | -4.63 (-4.86 to -4.39) | <0.001 |
| Norway | 5545  (2239-11180) | 111.78  (44.76-225.88) | 7255  (2888-14895) | 103.76  (40.87-215.5) | -0.24 (-0.42 to -0.05) | 0.012 |
| Oman | 977  (376-2152) | 76.91  (30.08-166.85) | 4023  (1598-8550) | 85.78  (34.05-175.01) | 0.36 (0.32 to 0.4) | <0.001 |
| Pakistan | 103485  (41650-206783) | 136.23  (55.07-272.3) | 266521  (104382-534096) | 139.47  (55.19-277.83) | 0.07 (0.05 to 0.09) | <0.001 |
| Palau | 9  (4-20) | 76.27  (30.71-162.92) | 4  (2-10) | 17.98  (6.61-39.11) | -4.55 (-4.75 to -4.36) | <0.001 |
| Palestine | 592  (231-1324) | 54.48  (21.52-120.76) | 2427  (973-5083) | 64.49  (25.67-133.85) | 0.55 (0.47 to 0.63) | <0.001 |
| Panama | 1728  (692-3578) | 92.98  (37.69-192.87) | 4382  (1732-9066) | 99.57  (39.25-206.31) | 0.22 (0.2 to 0.25) | <0.001 |
| Papua New Guinea | 1552  (605-3415) | 61.73  (24.11-132.46) | 972  (365-2154) | 13.34  (4.96-29.54) | -4.81 (-5.03 to -4.58) | <0.001 |
| Paraguay | 2203  (864-4689) | 79.73  (31.38-168.35) | 5306  (2108-11069) | 77.92  (31.14-161.54) | -0.07 (-0.1 to -0.03) | <0.001 |
| Peru | 11704  (4810-24571) | 73.8  (29.93-153.94) | 28017  (10953-59608) | 75.15  (29.43-159.74) | 0.07 (0.03 to 0.11) | <0.001 |
| Philippines | 29916  (11754-64662) | 72.21  (28.64-156.89) | 24637  (9711-51486) | 24.88  (9.74-51.05) | -3.36 (-3.67 to -3.06) | <0.001 |
| Poland | 30585  (12071-65296) | 72.21  (28.6-154.13) | 41305  (16450-84524) | 77.17  (30.8-159.89) | 0.2 (0.13 to 0.28) | <0.001 |
| Portugal | 7264  (2794-15679) | 60.63  (23.2-133.21) | 9047  (3491-18587) | 54.88  (21.07-114.66) | -0.34 (-0.51 to -0.16) | <0.001 |
| Puerto Rico | 4039  (1611-8160) | 112.53  (44.88-227.83) | 4700  (1856-9586) | 105.53  (41.4-220.98) | -0.22 (-0.27 to -0.16) | <0.001 |
| Qatar | 368  (143-775) | 96.15  (38.59-202.13) | 3575  (1410-7661) | 99.29  (38.83-204.8) | 0.11 (0.07 to 0.14) | <0.001 |
| Republic of Korea | 18343  (7007-41050) | 48.56  (18.68-105.75) | 44720  (17560-91907) | 53.48  (21.12-111.01) | 0.32 (0.24 to 0.39) | <0.001 |
| Republic of Moldova | 2911  (1178-6335) | 63.44  (25.74-137.68) | 3113  (1214-6463) | 59.39  (23.17-123.98) | -0.2 (-0.31 to -0.08) | 0.001 |
| Romania | 15197  (5780-33178) | 56.4  (21.77-123.41) | 17749  (6995-35919) | 63.08  (24.84-131.64) | 0.38 (0.29 to 0.47) | <0.001 |
| Russian Federation | 135230  (54324-284208) | 77.22  (31.02-161.99) | 142732  (56787-290219) | 70.92  (28.37-146.14) | -0.27 (-0.31 to -0.24) | <0.001 |
| Rwanda | 5229  (2066-10467) | 121.33  (48.4-244.37) | 6228  (2378-13352) | 67.62  (26.2-144.3) | -1.82 (-1.95 to -1.69) | <0.001 |
| Saint Kitts and Nevis | 35  (14-71) | 104.87  (42.01-211.88) | 76  (30-158) | 101.02  (40.11-211.96) | -0.12 (-0.19 to -0.05) | <0.001 |
| Saint Lucia | 99  (40-206) | 100.79  (40.79-209.3) | 213  (84-439) | 93.66  (36.54-194.27) | -0.23 (-0.3 to -0.15) | <0.001 |
| Saint Vincent and the Grenadines | 75  (30-155) | 96.6  (38.97-199.01) | 123  (49-252) | 92.37  (36.75-190.86) | -0.15 (-0.26 to -0.05) | 0.005 |
| Samoa | 67  (26-146) | 66.34  (26.35-143.45) | 24  (9-52) | 14.78  (5.51-32.1) | -4.71 (-4.92 to -4.5) | <0.001 |
| San Marino | 22  (8-47) | 74.63  (29.46-161.66) | 31  (12-64) | 61.74  (24.43-131.46) | -0.62 (-0.66 to -0.57) | <0.001 |
| Sao Tome and Principe | 118  (47-239) | 150.08  (60.68-300.1) | 212  (82-445) | 121.59  (47.73-251.24) | -0.67 (-0.76 to -0.59) | <0.001 |
| Saudi Arabia | 4558  (1833-9619) | 47.31  (18.78-101.36) | 21932  (8429-48243) | 55.28  (21.76-116.47) | 0.51 (0.44 to 0.59) | <0.001 |
| Senegal | 7288  (2939-14762) | 149.34  (60.73-298.84) | 13729  (5421-28412) | 118.8  (47.11-246.28) | -0.73 (-0.8 to -0.67) | <0.001 |
| Serbia | 6567  (2539-14343) | 57.2  (22.21-125.28) | 7471  (2896-15586) | 58.81  (22.83-122.56) | 0.1 (0.07 to 0.12) | <0.001 |
| Seychelles | 47  (19-101) | 80.72  (32.19-173.29) | 68  (26-144) | 53.44  (20.49-112.43) | -1.31 (-1.41 to -1.22) | <0.001 |
| Sierra Leone | 3229  (1259-6674) | 110.69  (42.69-227.53) | 11263  (4322-23082) | 167.83  (64.81-339.06) | 1.41 (1.36 to 1.47) | <0.001 |
| Singapore | 1602  (629-3502) | 56.81  (22.07-124.17) | 5122  (1985-10314) | 59.99  (23.28-120.73) | 0.17 (0.14 to 0.19) | <0.001 |
| Slovakia | 3335  (1296-7292) | 58.21  (22.66-127.97) | 5005  (1965-10196) | 63.8  (25.11-132.13) | 0.3 (0.27 to 0.33) | <0.001 |
| Slovenia | 2026  (820-4048) | 87.08  (35.24-173.57) | 2589  (1015-5368) | 85.79  (33.64-178.73) | -0.04 (-0.09 to 0) | 0.061 |
| Solomon Islands | 105  (42-237) | 57.33  (22.42-126.21) | 58  (22-124) | 12.2  (4.52-26.63) | -4.82 (-5.04 to -4.61) | <0.001 |
| Somalia | 4610  (1826-9697) | 102.14  (39.88-215.04) | 4449  (1684-9980) | 41.48  (15.77-91.04) | -2.84 (-3 to -2.67) | <0.001 |
| South Africa | 12783  (4940-28106) | 50.72  (19.55-110.81) | 19965  (7854-43884) | 36.78  (14.2-80.9) | -1.03 (-1.19 to -0.87) | <0.001 |
| South Sudan | 5130  (1999-10303) | 138.16  (55.79-274.33) | 3845  (1484-8293) | 65.89  (25.87-139.97) | -2.34 (-2.48 to -2.21) | <0.001 |
| Spain | 29051  (11102-63991) | 64.02  (24.37-143.04) | 10791  (4067-23543) | 14.9  (5.6-31.82) | -4.67 (-5.13 to -4.2) | <0.001 |
| Sri Lanka | 16523  (6530-33497) | 113.32  (45.16-232.04) | 12899  (4874-27795) | 48.37  (18.47-104.3) | -2.76 (-2.91 to -2.62) | <0.001 |
| Sudan | 6005  (2345-13152) | 50.56  (19.54-110.32) | 16622  (6436-36474) | 57.37  (22.33-124.6) | 0.41 (0.39 to 0.43) | <0.001 |
| Suriname | 324  (130-669) | 103.24  (41.45-211.35) | 606  (238-1258) | 94.8  (37.19-200.23) | -0.28 (-0.34 to -0.22) | <0.001 |
| Sweden | 8979  (3582-19234) | 80.28  (31.66-174.17) | 9975  (3972-20068) | 68.92  (27.44-140.28) | -0.38 (-0.57 to -0.19) | <0.001 |
| Switzerland | 6242  (2478-13383) | 71.94  (28.37-154.43) | 8362  (3280-17184) | 63.55  (25.17-132.67) | -0.41 (-0.57 to -0.26) | <0.001 |
| Syrian Arab Republic | 4062  (1606-8913) | 58.1  (22.85-125.52) | 9519  (3705-19842) | 63.85  (25.15-133.63) | 0.3 (0.28 to 0.33) | <0.001 |
| Taiwan (Province of China) | 10936  (4253-22982) | 60.46  (23.25-127.65) | 23664  (9140-49182) | 62.47  (24.22-131.82) | 0.09 (0.05 to 0.14) | <0.001 |
| Tajikistan | 1992  (796-4280) | 62.27  (24.84-133.14) | 4048  (1586-8816) | 49.75  (19.63-105.63) | -0.73 (-0.78 to -0.67) | <0.001 |
| Thailand | 30798  (12154-64872) | 66.42  (26.59-138.88) | 76313  (30305-156341) | 77.3  (30.51-159.31) | 0.51 (0.42 to 0.6) | <0.001 |
| Timor-Leste | 283  (109-628) | 60.83  (23.67-132.1) | 393  (150-875) | 41.52  (15.82-92.41) | -1.24 (-1.38 to -1.11) | <0.001 |
| Togo | 3266  (1279-6484) | 145.27  (58.38-287.86) | 6972  (2717-14937) | 110.87  (44.39-231.92) | -0.87 (-0.98 to -0.75) | <0.001 |
| Tokelau | 1  (0-2) | 65.57  (25.9-141.4) | 0  (0-0) | 15.58  (5.78-34.15) | -4.5 (-4.72 to -4.29) | <0.001 |
| Tonga | 42  (17-92) | 66.08  (26.2-145.12) | 13  (5-27) | 14.9  (5.53-32.15) | -4.7 (-4.9 to -4.5) | <0.001 |
| Trinidad and Tobago | 1097  (437-2247) | 107.53  (42.76-218.58) | 1810  (718-3724) | 104.11  (41.05-215.28) | -0.09 (-0.14 to -0.05) | <0.001 |
| Tunisia | 3789  (1495-8335) | 62.8  (25.04-136.85) | 10371  (4104-21504) | 74.98  (29.59-154.96) | 0.58 (0.55 to 0.61) | <0.001 |
| Turkey | 17715  (6826-39279) | 41.22  (15.93-90.65) | 82121  (32791-170518) | 85.02  (33.96-177.42) | 2.37 (2.23 to 2.51) | <0.001 |
| Turkmenistan | 1566  (614-3420) | 67.08  (26.48-145.62) | 3245  (1273-6743) | 65.82  (25.78-135.37) | -0.06 (-0.1 to -0.03) | <0.001 |
| Tuvalu | 5  (2-10) | 58.98  (22.9-127.19) | 2  (1-3) | 13.53  (4.98-28.98) | -4.67 (-5.15 to -4.2) | <0.001 |
| Uganda | 11519  (4490-23278) | 119.11  (47.33-243.4) | 15603  (6016-34141) | 67.47  (26.35-142.94) | -1.82 (-1.99 to -1.65) | <0.001 |
| Ukraine | 47544  (19268-100540) | 73.48  (29.64-154.71) | 41005  (16295-83721) | 64.48  (25.89-134.66) | -0.41 (-0.49 to -0.33) | <0.001 |
| United Arab Emirates | 1477  (581-3089) | 92.4  (37.06-190.51) | 13196  (5243-27917) | 91.68  (36.36-189.1) | -0.02 (-0.05 to 0.02) | 0.326 |
| United Kingdom | 28998  (11068-62481) | 40.37  (15.55-89.14) | 34845  (13163-73219) | 36.04  (13.79-77.91) | -0.43 (-0.59 to -0.27) | <0.001 |
| United Republic of Tanzania | 19475  (7755-39356) | 125.12  (50.37-253.06) | 26228  (10140-56607) | 70.94  (27.65-148.75) | -1.77 (-1.84 to -1.7) | <0.001 |
| United States of America | 181137  (71445-387960) | 63.33  (25.1-136.35) | 268951  (105736-536121) | 58.5  (23.31-119.15) | -0.26 (-0.37 to -0.15) | <0.001 |
| United States Virgin Islands | 120  (48-244) | 114.25  (45.95-231.41) | 130  (52-266) | 109  (42.64-229.48) | -0.16 (-0.23 to -0.1) | <0.001 |
| Uruguay | 3440  (1395-7057) | 103.57  (41.97-212.34) | 4413  (1760-8995) | 107.9  (42.59-223.8) | 0.14 (0.1 to 0.17) | <0.001 |
| Uzbekistan | 8224  (3205-17789) | 62.25  (24.56-134.2) | 19471  (7571-41320) | 58.2  (22.73-122.27) | -0.2 (-0.23 to -0.16) | <0.001 |
| Vanuatu | 53  (20-117) | 60.99  (23.37-132.99) | 29  (11-63) | 12.63  (4.67-27.8) | -4.94 (-5.15 to -4.73) | <0.001 |
| Venezuela (Bolivarian Republic of) | 13425  (5367-28013) | 97.94  (39.46-202.52) | 27634  (11086-57529) | 90.91  (36.27-188.79) | -0.22 (-0.26 to -0.19) | <0.001 |
| Viet Nam | 16213  (6265-35301) | 35.69  (13.5-77.13) | 50042  (19277-106343) | 45.5  (17.51-96.72) | 0.76 (0.63 to 0.88) | <0.001 |
| Yemen | 4187  (1685-8834) | 60.51  (23.93-125.72) | 14254  (5574-31296) | 64.06  (24.91-134.94) | 0.17 (0.1 to 0.23) | <0.001 |
| Zambia | 5872  (2309-11701) | 129.14  (51.43-259.57) | 8595  (3297-18649) | 73.91  (28.29-156.28) | -1.71 (-1.88 to -1.55) | <0.001 |
| Zimbabwe | 1752  (675-3776) | 32.54  (12.33-69.34) | 2964  (1132-6559) | 30.31  (11.6-66.8) | -0.23 (-0.3 to -0.15) | <0.001 |

AAPC: average annual percent change; ASR: age-standardized rate; DALYs, disability-adjusted life-years.
