# Supplementary material for: Global, regional, and national burden of periodontal diseases from 1990 to 2021 and predictions to 2040: an analysis of the global burden of disease study 2021
Source: Front Oral Health. 2025 Jul 24;6:1627746. doi: 10.3389/froh.2025.1627746 (PMC12332980; doi:10.3389/froh.2025.1627746)
Supplement: Supplementary file 5 [file Table4.docx]

**Supplementary Table S4** Contributions of aging, population growth, and epidemiological change to the variations in periodontal diseases DALYs from 1990 to 2021, stratified by SDI quintiles.

| **location** | **Overall difference** | **Aging** | **Population** | **Epidemiological change** | **percent change of Aging** | **percent change of Population** | **percent change of Epidemiological change** |
| --- | --- | --- | --- | --- | --- | --- | --- |
| Global | 3282765.28 | 701455.89 | 2494178.15 | 87131.24 | 21.37 | 75.98 | 2.65 |
| High SDI | 346160.65 | 116574.84 | 236905.37 | -7319.57 | 33.68 | 68.44 | -2.11 |
| High-middle SDI | 500653.01 | 217313.15 | 293809.86 | -10469.99 | 43.41 | 58.69 | -2.09 |
| Middle SDI | 1202457.94 | 394885.10 | 777316.52 | 30256.31 | 32.84 | 64.64 | 2.52 |
| Low-middle SDI | 933543.84 | 106885.09 | 801185.76 | 25472.99 | 11.45 | 85.82 | 2.73 |
| Low SDI | 298438.29 | -4931.19 | 449218.04 | -145848.57 | -1.65 | 150.52 | -48.87 |

DALYs, disability-adjusted life-years; SDI, socio-demographic index.
