# Supplementary material for: Global, regional, and national burden of periodontal diseases from 1990 to 2021 and predictions to 2040: an analysis of the global burden of disease study 2021
Source: Front Oral Health. 2025 Jul 24;6:1627746. doi: 10.3389/froh.2025.1627746 (PMC12332980; doi:10.3389/froh.2025.1627746)
Supplement: Supplementary file 6 [file Table5.docx]

**Supplementary Table S5** Projected global case numbers and ASR for incidence, prevalence, and DALYs of periodontal diseases to 2040.

| **Year** | **Incidence** | | **Prevalence** | | **DALYs** | |
| --- | --- | --- | --- | --- | --- | --- |
|  | **Number**  **(95%UI)** | **ASR**  **(95%UI)** | **Number**  **(95%UI)** | **ASR**  **(95%UI)** | **Number**  **(95%UI)** | **ASR**  **(95%UI)** |
| 1990 | 50806511  (50345407-51267615) | 111.66  (93.7-131.76) | 556849215  (556383512-557314918) | 17223.58  (17209.79-17237.38) | 3619452  (2977074-4357002) | 111.66  (93.7-131.76) |
| 1991 | 51553439  (51084454-52022423) | 111.06  (93.07-131.18) | 566203666  (565733419-566673913) | 17130.83  (17117.17-17144.49) | 3680334  (3024055-4431266) | 111.06  (93.07-131.18) |
| 1992 | 52320411  (51843773-52797049) | 110.5  (92.47-130.64) | 575834267  (575359656-576308877) | 17044.24  (17030.72-17057.77) | 3742821  (3072933-4506795) | 110.5  (92.47-130.64) |
| 1993 | 53126238  (52642095-53610380) | 110.01  (91.96-130.17) | 585793885  (585314965-586272804) | 16970.74  (16957.34-16984.14) | 3807353  (3124285-4584065) | 110.01  (91.96-130.17) |
| 1994 | 53997844  (53506336-54489352) | 109.67  (91.6-129.84) | 596457598  (595974259-596940936) | 16918.56  (16905.28-16931.85) | 3876441  (3180853-4665558) | 109.67  (91.6-129.84) |
| 1995 | 54977592  (54478720-55476463) | 109.53  (91.44-129.7) | 608014154  (607526136-608502171) | 16896.72  (16883.55-16909.89) | 3951376  (3243650-4752958) | 109.53  (91.44-129.7) |
| 1996 | 55994425  (55488009-56500840) | 109.44  (91.35-129.61) | 620404273  (619911416-620897130) | 16884.89  (16871.83-16897.95) | 4031440  (3311754-4845706) | 109.44  (91.35-129.61) |
| 1997 | 56955655  (56441357-57469952) | 109.28  (91.18-129.45) | 632861032  (632363307-633358757) | 16861.27  (16848.33-16874.21) | 4111911  (3380259-4939221) | 109.28  (91.18-129.45) |
| 1998 | 57913238  (57390650-58435826) | 109.1  (91-129.28) | 645527210  (645024412-646030008) | 16834.96  (16822.14-16847.78) | 4193644  (3449875-5034682) | 109.1  (91-129.28) |
| 1999 | 58936929  (58405674-59468185) | 109  (90.9-129.17) | 658929379  (658421070-659437688) | 16819.85  (16807.14-16832.56) | 4280121  (3523924-5135549) | 109  (90.9-129.17) |
| 2000 | 60097930  (59557683-60638177) | 109.03  (90.93-129.2) | 673260248  (672745876-673774621) | 16825.55  (16812.94-16838.16) | 4372564  (3603557-5243045) | 109.03  (90.93-129.2) |
| 2001 | 61396090  (60846422-61945758) | 109.13  (91.05-129.3) | 688342924  (687821787-688864060) | 16841.61  (16829.09-16854.12) | 4470384  (3688407-5356407) | 109.13  (91.05-129.3) |
| 2002 | 62755013  (62195508-63314519) | 109.21  (91.14-129.37) | 703624227  (703095866-704152587) | 16852.47  (16840.05-16864.9) | 4569750  (3774620-5471645) | 109.21  (91.14-129.37) |
| 2003 | 64154471  (63584933-64724009) | 109.3  (91.24-129.45) | 719341906  (718806175-719877637) | 16862.99  (16850.65-16875.33) | 4672308  (3863582-5590272) | 109.3  (91.24-129.45) |
| 2004 | 65564588  (64985001-66144175) | 109.41  (91.36-129.55) | 735761276  (735218239-736304313) | 16878.59  (16866.34-16890.84) | 4779057  (3956040-5713178) | 109.41  (91.36-129.55) |
| 2005 | 66959736  (66370325-67549147) | 109.54  (91.5-129.69) | 752838049  (752288110-753387987) | 16898.84  (16886.68-16911) | 4889792  (4051565-5840258) | 109.54  (91.5-129.69) |
| 2006 | 67978643  (67379067-68578220) | 108.96  (90.9-129.13) | 765199200  (764643269-765755132) | 16809.39  (16797.34-16821.45) | 4969651  (4115097-5936901) | 108.96  (90.9-129.13) |
| 2007 | 68500803  (67890282-69111324) | 107.36  (89.26-127.56) | 770273242  (769712226-770834258) | 16561.7  (16549.77-16573.63) | 5002160  (4130275-5986911) | 107.36  (89.26-127.56) |
| 2008 | 68866027  (68244301-69487752) | 105.41  (87.27-125.66) | 772732125  (772166442-773297809) | 16261.38  (16249.58-16273.19) | 5017655  (4127786-6020266) | 105.41  (87.27-125.66) |
| 2009 | 69423088  (68790650-70055525) | 103.84  (85.66-124.13) | 777749309  (777178891-778319727) | 16019.81  (16008.13-16031.49) | 5049584  (4141857-6069439) | 103.84  (85.66-124.13) |
| 2010 | 70530987  (69889459-71172514) | 103.4  (85.21-123.71) | 791028122  (790452658-791603585) | 15951.64  (15940.06-15963.22) | 5135440  (4211276-6170370) | 103.4  (85.21-123.71) |
| 2011 | 72438483  (71789791-73087175) | 104.73  (86.54-125) | 817819971  (817238217-818401724) | 16155.37  (16143.86-16166.89) | 5308873  (4370365-6356018) | 104.73  (86.54-125) |
| 2012 | 74906005  (74251125-75560884) | 107.35  (89.19-127.56) | 855468366  (854878897-856057835) | 16559.53  (16548.06-16571.01) | 5552599  (4601183-6610011) | 107.35  (89.19-127.56) |
| 2013 | 77556589  (76895822-78217356) | 110.4  (92.26-130.53) | 897549131  (896951441-898146821) | 17028.4  (17016.95-17039.85) | 5824965  (4861365-6891910) | 110.4  (92.26-130.53) |
| 2014 | 80009506  (79342558-80676453) | 112.99  (94.88-133.05) | 936957871  (936352394-937563348) | 17429.09  (17417.68-17440.5) | 6079455  (5103893-7156292) | 112.99  (94.88-133.05) |
| 2015 | 81898520  (81224437-82572602) | 114.29  (96.2-134.33) | 966316813  (965704927-966928699) | 17630  (17618.64-17641.35) | 6268963  (5280969-7357289) | 114.29  (96.2-134.33) |
| 2016 | 83270327  (82588059-83952595) | 114.33  (96.26-134.36) | 984970103  (984352631-985587575) | 17635.59  (17624.3-17646.87) | 6388935  (5388891-7490101) | 114.33  (96.26-134.36) |
| 2017 | 84504539  (83813541-85195537) | 113.93  (95.89-133.95) | 999726688  (999103398-1000349978) | 17574.19  (17562.98-17585.41) | 6483153  (5471939-7597455) | 113.93  (95.89-133.95) |
| 2018 | 85722027  (85022146-86421907) | 113.49  (95.48-133.51) | 1013963221  (1013334034-1014592407) | 17506.74  (17495.58-17517.89) | 6574070  (5551724-7701582) | 113.49  (95.48-133.51) |
| 2019 | 87038441  (86329996-87746887) | 113.4  (95.41-133.43) | 1031115680  (1030480746-1031750614) | 17494.46  (17483.37-17505.55) | 6683458  (5649244-7824708) | 113.4  (95.41-133.43) |
| 2020 | 88334307  (87617740-89050875) | 113.36  (95.37-133.39) | 1048714646  (1048074691-1049354600) | 17502.65  (17491.63-17513.67) | 6790608  (5743959-7945487) | 113.36  (95.37-133.39) |
| 2021 | 89512354  (88787852-90236856) | 113.45  (95.47-133.47) | 1065897400  (1065252166-1066542634) | 17525.57  (17514.6-17536.54) | 6897199  (5839153-8064560) | 113.45  (95.47-133.47) |
| 2022 | 91008048  (87571763-94444333) | 113.27  (88.73-140.6) | 1081551857  (1035958330-1127145384) | 17494.26  (16756.4-18232.13) | 6999699  (5531359-8619992) | 113.27  (88.73-140.6) |
| 2023 | 92360954  (88143017-96578892) | 113.48  (70.06-164.23) | 1100466996  (1041312469-1159621524) | 17536.72  (16593.53-18479.92) | 7117096  (4466234-10170843) | 113.48  (70.06-164.23) |
| 2024 | 93776636  (88110433-99442838) | 113.71  (44.07-202.65) | 1120471173  (1040174540-1200767806) | 17581.99  (16321.29-18842.7) | 7241051  (2889829-12665441) | 113.71  (44.07-202.65) |
| 2025 | 95202436  (87615198-102789674) | 113.96  (27.18-234) | 1140903735  (1033193889-1248613580) | 17630.4  (15965-19295.8) | 7367561  (1826142-14777993) | 113.96  (27.18-234) |
| 2026 | 96626420  (86965221-106287619) | 114.24  (18.47-254.56) | 1161662476  (1021160942-1302164009) | 17683.6  (15543.56-19823.64) | 7496101  (1261917-16261474) | 114.24  (18.47-254.56) |
| 2027 | 98047499  (86252088-109842910) | 114.56  (13.69-267.29) | 1182606240  (1004392974-1360819506) | 17741.46  (15066.34-20416.58) | 7625739  (949480-17281252) | 114.56  (13.69-267.29) |
| 2028 | 99464540  (85439979-113489101) | 114.91  (11.25-275.2) | 1203698409  (983146098-1424250720) | 17804.65  (14540.35-21068.95) | 7756126  (790225-18011588) | 114.91  (11.25-275.2) |
| 2029 | 100876494  (84479700-117273287) | 115.3  (9.85-280.15) | 1225150925  (957707324-1492594527) | 17874.12  (13969.82-21778.42) | 7888709  (699213-18561803) | 115.3  (9.85-280.15) |
| 2030 | 102291126  (83343034-121239218) | 115.73  (9.01-283.27) | 1247252970  (928279449-1566226491) | 17950.56  (13356.77-22544.35) | 8025413  (647413-18999253) | 115.73  (9.01-283.27) |
| 2031 | 103704852  (82003155-125406549) | 116.22  (8.55-285.23) | 1269990266  (894711576-1645268956) | 18035.25  (12702.04-23368.46) | 8166191  (621750-19364442) | 116.22  (8.55-285.23) |
| 2032 | 105119609  (80444899-129794318) | 116.77  (8.26-286.46) | 1293261137  (856719663-1729802610) | 18127.94  (12004.09-24251.78) | 8310364  (609547-19684046) | 116.77  (8.26-286.46) |
| 2033 | 106529901  (78658418-134401384) | 117.37  (8.03-287.32) | 1317004452  (814095295-1819913610) | 18229.43  (11262.53-25196.32) | 8457447  (600445-19977196) | 117.37  (8.03-287.32) |
| 2034 | 107932358  (76638039-139226677) | 118.04  (7.76-288.06) | 1341454281  (766755806-1916152757) | 18341.52  (10476.61-26206.42) | 8609017  (586643-20260701) | 118.04  (7.76-288.06) |
| 2035 | 109331969  (74383851-144280088) | 118.79  (7.36-288.9) | 1366923817  (714554133-2019293501) | 18465.3  (9643.99-27286.61) | 8767148  (563388-20550335) | 118.79  (7.36-288.9) |
| 2036 | 110730806  (71891097-149570515) | 119.62  (6.76-289.99) | 1393437517  (657065674-2129809360) | 18601.65  (8760.99-28442.31) | 8932039  (524786-20858453) | 119.62  (6.76-289.99) |
| 2037 | 112137561  (69156590-155118532) | 120.53  (5.91-291.46) | 1420986997  (593763575-2248210418) | 18750.38  (7822.3-29678.47) | 9103636  (464449-21198028) | 120.53  (5.91-291.46) |
| 2038 | 113549354  (66173938-160924771) | 121.53  (4.78-293.47) | 1449526239  (524119926-2374932552) | 18912.7  (6823.38-31002.02) | 9281598  (380388-21580695) | 121.53  (4.78-293.47) |
| 2039 | 114965153  (62936928-166993378) | 122.65  (3.46-296.15) | 1479295591  (447628523-2510962659) | 19091.38  (5759.03-32423.73) | 9467533  (278306-22019040) | 122.65  (3.46-296.15) |
| 2040 | 116389865  (59440458-173339273) | 123.88  (1.85-299.65) | 1510627625  (363652820-2657602430) | 19288.13  (4621.97-33954.3) | 9663598  (151471-22526760) | 123.88  (1.85-299.65) |

ASR: age-standardized rate; DALYs: disability-adjusted life-years.
